# Supplementary material for: A multicenter, single-arm study using a modified faricimab treat-and-extend regimen in patients with macular edema due to central retinal vein occlusion: RVOSTAR study design protocol
Source: PLoS One. 2025 Oct 30;20(10):e0335015. doi: 10.1371/journal.pone.0335015 (PMC12574838; doi:10.1371/journal.pone.0335015)
Supplement: S1 Table — (PDF) [file pone.0335015.s001.pdf]

S1 Table. Full exclusion criteria.

Key exclusion criteria[a]
• Systemic treatment for suspected or active systemic infection<br>• Stroke (cerebral vascular accident) or myocardial infarction within 6 months<br>• Uncontrolled blood pressure (defined as systolic >180 mmHg and/or diastolic >110 mmHg while a patient is at rest)[a]<br>• Active cancer within 12 months, except for appropriately treated carcinoma in situ of the cervix, non-melanoma skin carcinoma, and prostate cancer with a Gleason score of ≤6 and a stable prostate-specific antigen for >12 months<br>• Significant disease, significant surgical procedure, or systemic steroids (including oral and injected) within 1 month<br>• History or presence of other diseases, metabolic dysfunction, physical examination finding, or clinical laboratory finding that may affect the ability to receive faricimab or participate in the study, or which may affect interpretation of the results<br>• Pregnancy, breastfeeding, or women of child-bearing potential with an intent to become pregnant (must agree to remain abstinent, or use acceptable contraceptive methods during the treatment period and for ≥3 months after the final dose of study treatment)<br>• History of severe allergic reactions or anaphylaxis to a biologic agent, or known hypersensitivity to any component of faricimab, drugs used in the study procedures (including fluorescein), dilating drops, or any of the anesthetic and antimicrobial preparations used by a patient during the study<br>• Participation in an ophthalmologic clinical trial that involves treatment with any drug (with the exception of vitamins and minerals) or device within 3 months<br>• Requirement for continuous use of any prohibited medications and treatments (including systemic anti-VEGF; IVT anti-VEGF in the study eye; drugs known to cause ME [e.g., fingolimod, tamoxifen]; IVT, periocular, or chronic topical corticosteroids in the study eye; verteporfin in the study eye; administration of micropulse and focal/grid laser in the study eye; or any other experimental therapies, except for vitamins and minerals)
For the study eye:
• CRVO-ME or HRVO-ME, or persistent CRVO-ME or HRVO-ME, diagnosed >4 months before screening<br>• History of retinal detachment or macular hole (stage 3 or 4)<br>• Any current ocular condition that may cause irreversible vision loss (in the opinion of the investigator) other than CRVO-ME or HRVO-ME in the study eye (e.g., ischemic

maculopathy, Irvine-Gass syndrome, foveal atrophy, foveal fibrosis, pigment abnormalities, dense subfoveal hard exudates, or other non-retinal conditions)

- Tractional retinal detachment, full-thickness macular hole, vitreomacular traction, or epiretinal membrane involving the fovea or disrupting the macular architecture in the study eye
- Diagnosis of moderate non-proliferative diabetic retinopathy, worse proliferative diabetic retinopathy, DME, nAMD, geographic atrophy, or myopic choroidal neovascularization as assessed by the investigator
- Active rubeosis, angle neovascularization, neovascular glaucoma
- Cataract surgery or treatment for complications of cataract surgery (YAG laser capsulotomy) within 3 months
- Any other intraocular surgery (e.g., pars plana vitrectomy, scleral buckle, glaucoma surgery, corneal transplant, or radiotherapy)
- Macular laser (focal/grid) or PRP in the study eye, or PRP scheduled within 3 months
- Any prior intervention with PDT, laser, transpupillary thermotherapy, or vitreoretinal surgery including sheathotomy
- Any prior or current treatment for ME, macular neovascularization (including DME and nAMD), or vitreomacular-interface abnormalities, including IVT treatment (e.g., anti-VEGF, steroids, tissue plasminogen activator, C_3F_8, SF_6, air) or periocular injection
- Prior periocular or IVT treatment (including anti-VEGF) for other retinal diseases

For both eyes:

- Any history of idiopathic or immune-mediated uveitis in either eye
- Active ocular inflammation or suspected or active ocular or periocular infection in either eye

CRVO-ME, macular edema due to central retinal vein occlusion; DME, diabetic macular edema; HRVO-ME, macular edema due to hemi-retinal vein occlusion; IVT, intravitreal, ME, macular edema; mmHg, millimeters of mercury; nAMD, neovascular age-related macular degeneration; PDT, photodynamic therapy; PRP, panretinal photocoagulation; VEGF, vascular endothelial growth factor; YAG, Yttrium Aluminum Garnet.

[a]If a patient's initial reading during the screening period exceeds these values, a second reading may be obtained later the same day or on another day during the screening period.
