# Supplementary material for: A multicenter, single-arm study using a modified faricimab treat-and-extend regimen in patients with macular edema due to central retinal vein occlusion: RVOSTAR study design protocol
Source: PLoS One. 2025 Oct 30;20(10):e0335015. doi: 10.1371/journal.pone.0335015 (PMC12574838; doi:10.1371/journal.pone.0335015)

S1 Fig. RVOSTAR Dosing Regimen.

CST, central subfield thickness; Q4W, every 4 weeks; T&E, treat-and-extend.

[a]Change from the reference CST, which is the value at the visit at which resolution of macular edema is confirmed.

[b]No maximum dosing interval.

[c]The minimum dosing interval is 4 weeks.

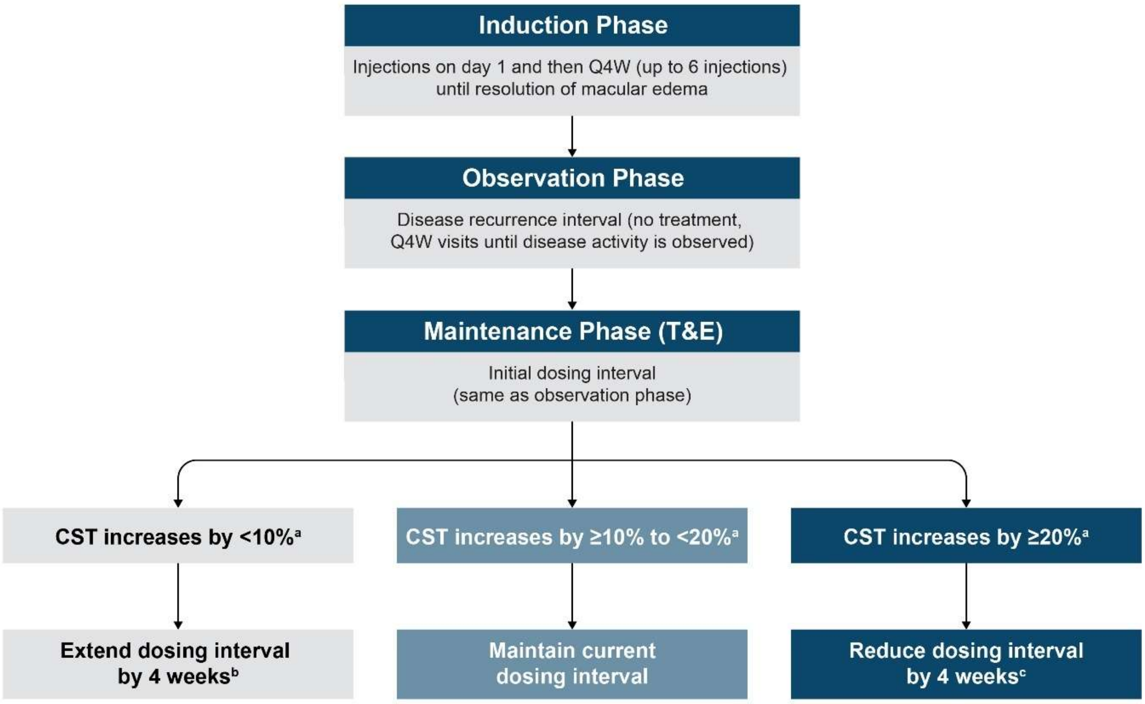

Supplement: S1 Fig — (PDF) [file pone.0335015.s002.pdf]
