# Supplementary material for: A multicenter, single-arm study using a modified faricimab treat-and-extend regimen in patients with macular edema due to central retinal vein occlusion: RVOSTAR study design protocol
Source: PLoS One. 2025 Oct 30;20(10):e0335015. doi: 10.1371/journal.pone.0335015 (PMC12574838; doi:10.1371/journal.pone.0335015)
Supplement: S1 File — RVOSTAR study protocol (English). (PDF) [file pone.0335015.s003.pdf]

PROTOCOL

TITLE:	A Study of Modified Treat-and-Extend (mTAE) Regimen Using Faricimab in Patients with Macular Edema Secondary to Central Retinal Vein Occlusion (CRVO)<br>Abbreviated Title: RVO treatment Strategy with modified Treat And extend Regimen of faricimab (RVOSTAR) study
PROTOCOL NUMBER:	CMA-0210
VERSION NUMBER:	1.0
DATE FINAL:	February 28, 2025
TEST PRODUCT:	Faricimab (genetical recombination)
PRINCIPAL INVESTIGATOR:	Department of Ophthalmology, Mie University Graduate School of Medicine<br>Mineo Kondo, Professor
COLLABORATING RESEARCH INSTITUTE:	Chugai Pharmaceutical Co., Ltd.
CLINICAL STUDY OFFICE:	Department of Ophthalmology, Mie University Graduate School of Medicine<br>Mineo Kondo, Professor
SUPPORT OFFICE:	IQVIA Services Japan G.K.
CRB APPROVAL DATE:	Month XX, 2025

TABLE OF CONTENTS

PROTOCOL 1
PROTOCOL SYNOPSIS 7
List of Abbreviations and Definitions of Terms 16
1. Background 18
1.1 Background on the Disease under Study 18
1.2 Background on Faricimab 18
1.3 Rationale for Study Plan 19
2. Objectives and Endpoints 19
2.1 Objectives 19
2.2 Endpoints 19
2.2.1 Primary Endpoint 20
2.2.2 Secondary Endpoints 20
2.2.3 Exploratory Endpoints 20
2.2.4 Safety Endpoints 21
3. Overview of the Study 21
3.1 Study Type 21
3.2 Study Design 21
3.3 End of Study and Duration of Study 22
3.4 Rationale for Study Design 22
3.5 Rationale for the Target Sample Size 23
3.6 Significance of Study 24
3.7 Potential Benefits and Disadvantages Associated with Study Participation 24
4. Materials and Methods 26
4.1 Patients 26
4.1.1 Inclusion Criteria 26
4.1.2 Exclusion Criteria 26
4.2 Enrollment 28
4.2.1 Enrollment Procedures 28
4.2.2 Issuance and Notification of Enrollment Results 29
4.2.3 Enrollment in Multiple Studies 29
4.2.4 Notes on Enrollment 29
4.2.5 Procedures for Closing Enrollment 29
4.3 Schedule of Study Assessments 30
4.3.1 Informed Consent Forms and Screening Log 30
4.3.2 Medical and Surgical History and Patient Demographics 30
4.3.3 Blood Pressure 30

4.3.4 Ocular Tests 31
4.3.4.1 Intraocular Pressure (IOP) Measurement 31
4.3.4.2 Refraction Test 31
4.3.4.3 Axial Length Test 31
4.3.4.4 Visual Acuity Test 31
4.3.4.5 Fundoscopy 31
4.3.5 Optical Coherence Tomography (OCT) 32
4.3.6 Unscheduled Visits 32
4.3.7 Patient Discontinuation from Study 32
4.3.8 Site Discontinuation 33
5. Treatment Plans and Criteria for Adjusting Treatment 33
5.1 Study Drug 33
5.2 Protocol Treatment 33
5.3 Protocol Treatment Interruption/Discontinuation Criteria 35
5.4 Concomitant and Supportive Therapies 37
5.4.1 Specified Concomitant and Supportive Therapies 37
5.4.2 Concomitant and Supportive Therapies That Are Recommended/Not Recommended 37
5.4.3 Concomitant and Supportive Therapies That Are Permitted 37
5.4.4 Prohibited Medications and Therapies 37
5.5 Subsequent Therapy 38
6. Safety Assessments 38
6.1 Anticipated Adverse Drug Events of Individual Drugs 38
6.2 Definition of Adverse Events 38
6.2.1 Definition of Adverse Events 38
6.2.2 Definition of Illness 38
6.2.3 Grade of Adverse Events 38
6.2.4 Assessment of Severity of Adverse Events 39
6.2.5 Causality Assessment Criteria 39
7. Reporting of Device Malfunctions That May Lead to Adverse Events or Injuries 40
7.1 Reporting Period for Device Malfunctions That May Lead to Adverse Events or Injuries 40
7.2 Management of All Device Malfunctions That May Lead to Adverse Events or Injuries 40
7.3 Management of Illnesses and Infections 40
7.4 Follow-up of Patients after Adverse Events 41
7.5 Pregnancies in Female Patients 42
7.6 Reporting of Overdose/Medication Error/Drug Abuse/Drug Misuse 42

8. Statistical Considerations and Analysis Plan 42
8.1 Planned Enrollment and Planned Overall Duration of Study 42
8.2 Analysis Populations 42
8.3 Primary Endpoint Analysis 42
8.4 Secondary Endpoint Analysis 43
8.5 Exploratory Endpoint Analysis 43
8.6 Safety Endpoint Analysis 44
8.7 Interim Analysis 44
8.8 End of Study 44
9. Data Collection and Management 44
9.1 Data Quality Assurance 44
9.2 Electronic Case Report Forms (eCRFs) 44
9.2.1 eCRF Items 44
9.3 Source Data Documentation 45
9.3.1 Data Entered into eCRFs Only 45
9.3.2 Source Documents 45
9.3.3 Use of Computerized Systems 45
9.4 Handling and Retention of Records, Information, and Samples 45
9.4.1 Study Site 45
9.4.2 Collaborating Research Institute 46
9.4.3 Support Office 46
9.4.4 Image Analysis Institution 46
9.5 Retention and Destruction of Information Provision Records 46
10. Ethical Considerations 47
10.1 Protection of Patients 47
10.2 Informed Consent 47
10.2.1 Informed Consent 47
10.2.2 Withdrawal of Consent 48
10.3 Handling of Inquiries from Patients 48
10.4 Protection of Personal Information and Identification of Patients 49
10.4.1 Purpose of Use of Personal Information, Information to be Used, and Methods for Use 49
10.4.2 Secondary Use of Data 50
10.4.3 Handling Requests for Information Disclosure 50
10.4.4 Information Management System 50
10.5 Necessity and System of Genetic Counseling 50
10.6 Protocol Compliance 50
10.7 Approval by Certified Review Board and Submissions to Ministry of Health, Labour

and Welfare 50
10.7.1 Initial Application Procedures..51
10.7.2 Procedures Taken by the Study Site Investigator 51
10.7.3 Permission for Conducting the Study at the Study Site 51
10.8 Change Application Procedures 51
10.8.1 Procedures Taken by the Principal Investigator 51
10.8.2 Procedures Taken by the Study Site Investigator 51
10.8.3 Study Progress and Review and Approval of Study Continuation (Periodic Report)...52
10.8.4 Reporting of the Progress of the Clinical Study..52
10.9 Conflicts of Interest 52
10.9.1 Management of Study-Related Conflicts of Interest..52
10.9.2 Study Funding Source/Funding Provider and Financial Interests......................52
10.9.3 Information Disclosure 53
10.10 Study Costs 53
10.10.1 Costs of Treatment..53
10.10.2 Compensation for Injuries 53
11. Quality Control and Quality Assurance 53
11.1 Study Records...53
11.2 Monitoring...54
11.2.1 Site Visit Monitoring 54
11.2.2 Audits..54
11.3 Protocol Deviations and Violations 54
12. Publication of Data 55
12.1 Public Database Registration 55
12.2 Publication of Data..55
12.3 Layperson Summary 56
13. Study Termination, Suspension, or Completion 56
13.1 Study Completion..56
13.2 Study Termination or Suspension...56
13.3 Clinical Study Report...56
13.4 Procedures at Study Completion...56
14. Ownership of Study Results 56
15. Study Administrative Structure..57
15.1 Study Administrator...57
15.1.1 Principal Investigator...57
15.2 Collaborating Research Institute..57
15.2.1 Head of the Collaborating Research Institute..57

15.2.2 Supervisory Manager of the Collaborating Research Institute 57
15.2.3 Manager of the Collaborating Research Institute 58
15.3 Funding Provider 59
15.4 Clinical Study Office 59
15.5 Study Sites 59
15.6 Person Responsible for Statistical Analysis 59
15.7 Support Office 60
15.8 Organization Responsible for Monitoring 60
15.9 Organization Responsible for Data Management 60
15.10 Auditing 60
15.11 Person Responsible for Coordination and Management 60
15.12 Image Analysis Institution 61
15.13 Study Executive Committee 61
15.14 Medical Expert Advisor (Image Analysis) 61
16. References 63

LIST OF APPENDICES

Appendix 1 Schedule of Activities 67
Appendix 2 eCRF Entries 69

List of Attachments

Attachment 1 List of Study Sites and Investigators
Attachment 2 Reporting Form for Overdose/Medication Error/Drug Abuse/Drug Misuse
Attachment 3 Reporting Form for Pregnancy
Attachment 4 Reporting Form for Newborn Child

PROTOCOL SYNOPSIS

TITLE:	A Study of Modified Treat-and-Extend (mTAE) Regimen Using Faricimab in Patients with Macular Edema Secondary to Central Retinal Vein Occlusion (CRVO)<br>Abbreviated Title: RVO treatment Strategy with modified Treat And extend Regimen of faricimab (RVOSTAR) study
PROTOCOL NUMBER:	CMA-0210
VERSION NUMBER:	1.0
TEST PRODUCT:	Faricimab (genetical recombination)
INDICATION:	Patients with macular edema secondary to central or hemispherical retinal vein occlusion
STUDY ADMINISTRATOR:	Division of Clinical Medical Science, Mie University Graduate School of Medicine

I Objectives and Endpoints

(1) Objectives

To evaluate the efficacy and extension of dosing intervals of faricimab, as well as to explore the factors related to efficacy and dosing intervals using a dosing regimen that is workable in clinical practice (mTAE) in treatment-naive patients with macular edema secondary to central retinal vein occlusion (CRVO) or hemispherical retinal vein occlusion (HRVO).

(2) Endpoints

① Primary Endpoint

- Change from baseline in best corrected visual acuity (BCVA) at Week 72
  BCVA will be measured in decimal visual acuity at each measurement time point and converted to logarithm of the minimum angle of resolution (logMAR) to calculate the change in BCVA.

② Secondary Endpoints

The following items will be evaluated at time points specified in the Statistical Analysis Plan (SAP) for each endpoint up to Week 72:

- BCVA and change from baseline in BCVA (logMAR)
- Proportion of patients with a ≥0.3 logMAR improvement from baseline
- Proportion of patients without a ≥0.3 logMAR worsening from baseline
- Central subfield thickness (CST) and change from baseline in CST
- Proportion of patients for each dosing interval of faricimab
- Mean number of faricimab injections

The following items will be evaluated at Week 72:

- Number of days of the observation phase after induction of faricimab

- Proportion of patients who did not receive additional doses of faricimab during the observation phase after induction of faricimab

The following items will be evaluated at time points (specified in the SAP) for each endpoint up to Week 72 by the presence or absence of retinal ischemia at baseline. Retinal ischemia at baseline is defined as ≥10 disc areas of capillary occlusion on Early Treatment Diabetic Retinopathy Study (ETDRS) 7-field fundus photography (or an equivalent range), and all others are defined as non-retinal ischemia.

- BCVA and change from baseline in BCVA
- CST and change from baseline in CST
- Proportion of patients for each dosing interval of faricimab
- Mean number of faricimab injections

③ Exploratory Endpoints

The following items will be evaluated at time points specified for each endpoint up to Week 72:

- Proportion of patients with resolution of macular edema
- Proportion of patients with absence of intraretinal fluid (IRF), proportion of patients with absence of subretinal fluid (SRF), and proportion of patients with absence of both
- Fluid volumes (IRF and SRF) and their change from baseline
- Proportion of patients with absence of retinal ischemia (assessed by fluorescein angiography [FA])
- The macular and total retinal areas of ischemic non-perfusion (capillary loss) and their change from baseline (assessed by FA)
- Proportion of patients who transitioned from non-ischemic to ischemic CRVO (assessed by FA)
- Area of vascular leakage in the macula and in the total retinal area and its change from baseline (assessed by FA)
- Proportion of patients with absence of vascular leakage (assessed by FA)
- Change from baseline in vascular density of the superficial, deep, and whole capillary plexuses (assessed by OCT-angiography [OCT-A])
- Relationships between baseline parameters and change from baseline in BCVA, change from baseline in CST, number of injections, dosing interval, and other efficacy parameters*
- Exploration of factors affecting the dosing interval at Week 72 *
- Relationships between variation of CST up to Week 24 (standard deviation [SD] of each patient based on CST values from Week 4 to Week 24) and BCVA, dosing interval, and other efficacy parameters *
- Comparison of parameters at baseline and Week 72 of patients who received multiple injections versus patients who received only one injection of faricimab during the induction phase *
- Number of days to achieve the best BCVA (highest decimal visual acuity value) after initiation of faricimab
- Number of days to achieve the best CST (lowest CST value) after initiation of faricimab
- Area under the curve (AUC) of BCVA from Day 1 to Week 24

- Proportion of loss of peak vision (0.1, 0.2, and ≥0.3 logMAR worsening from best BCVA) and exploration of contributing demographic factors *
- Incidence of new epimacular membranes in patients with absence of epimacular membranes at baseline

* Factors to be analyzed and the methods of analysis will be specified in the SAP.

④ Safety Endpoints

- Incidence and severity of ocular adverse events
- Incidence and severity of non-ocular adverse events

II Study Design

This study is an unmasked, single-arm, multicenter, prospective interventional study to investigate the efficacy and safety of faricimab dosing regimen (mTAE regimen) in treatment-naive patients with macular edema secondary to CRVO or HRVO. This study is a “specified clinical trial” under the Clinical Trials Act.

Patients who take part in this study will receive intravitreal (IVT) injections of 6.0 mg faricimab. The duration of participation is 72 weeks; all patients will receive faricimab on Day 1, after which patients will receive faricimab or undergo observation according to each criteria of the induction phase, observation phase, and maintenance phase. Study visits will occur every 4 weeks from Day 1 through Week 24, then after Week 24, patients will come to the study site at a frequency depending on the dosing interval, with scheduled visits at Week 36 (only patients in the observation phase), Week 52, and Week 72.

1) Induction Phase

The induction phase will be the period from faricimab initiation until first observation of resolution of macular edema (or until Week 20 if resolution of macular edema is not observed).

All patients will receive one injection of faricimab on Day 1 and every 4 weeks thereafter. After Week 4, if resolution of macular edema is observed before treatment, faricimab will not be administered and the patient will enter the observation phase. The CST value at this visit will be the reference CST.

The maximum number of faricimab injections to be administered during the induction phase is 6. If macular edema does not resolve after 6 consecutive injections by Week 20, the patient will enter the maintenance phase without undergoing the observation phase with the next scheduled injection at Week 24. In this case, the lowest CST value obtained from Day 1 through Week 20 will be the reference CST.

Of note, resolution of macular edema will be determined by the investigator based on the protocol-defined criteria; even in cases where these criteria are not met, the investigator may consider that macular edema has resolved, with recording of the reason for such judgment.

2) Observation Phase

The observation phase is defined as the period from the day of the visit when resolution of macular edema is observed until the day disease activity is observed again.

During the observation phase, faricimab will not be administered, and patients will be monitored every 4 weeks until Week 24 and at scheduled visits at Week 36, Week 52, and Week 72 thereafter, unless disease activity is observed.

If disease activity is observed at a visit, faricimab will be administered on that day or within the visit window (-7 to +14 days of the scheduled visit), and the patient will enter the maintenance phase. Even if a patient does not meet the disease activity criteria, if he or she has a significant decrease in visual acuity compared to the last visit (e.g., decimal visual acuity of 0.6 to 0.3) and has edema that requires treatment as judged by the investigator, he or she will be considered to have disease activity and administration of faricimab will be permitted. On the other hand, even if a patient meets the disease activity criteria, if he or she has no subjective symptoms and the change is considered to be of no clinical significance by the investigator, he or she may be judged to have no disease activity. The investigator should record the reasons for such judgments.

Patients will be monitored at unscheduled visits in between scheduled visits if deemed necessary by the investigator. If disease activity is observed at an unscheduled visit, the patient will be given faricimab and enter the maintenance phase.

3) Maintenance Phase

The initial dosing interval during the maintenance phase will be the same duration as the observation phase (or 4 weeks if the patient entered the maintenance phase without undergoing the observation phase). Dosing intervals during the maintenance phase will be adjusted every 4 weeks according to the following adjustment criteria. Dosing intervals will be adjusted by ±4 weeks, with a minimum dosing interval of 4 weeks and no limits on the maximum dosing interval. If the investigator deems that the patient needs an adjustment that differs from this adjustment criteria, the dosing interval will be adjusted as such, with recording of the reason for the judgment.

CST percent change from reference CST (%)	Dosing interval adjustment
<+10%	Extend interval (+4 weeks)
≥+10% to <+20%	Maintain interval (±0 weeks)
≥+20%	Reduce interval (-4 weeks)

Even if a patient does not meet the disease activity criteria, if he or she has a significant decrease in visual acuity compared to the last visit (e.g., decimal visual acuity of 0.6 to 0.3) and has edema that requires treatment as judged by the investigator, he or she will be considered to have disease activity and dosing interval will be reduced. On the other hand, even if a patient meets the disease activity criteria, if he or she has no symptoms and the change is considered to be of no clinical significance by the investigator, he or she may be considered to have no disease activity with recording of the reason for such judgment, and the dosing interval may be extended or maintained.

The study schedule diagram is shown below.

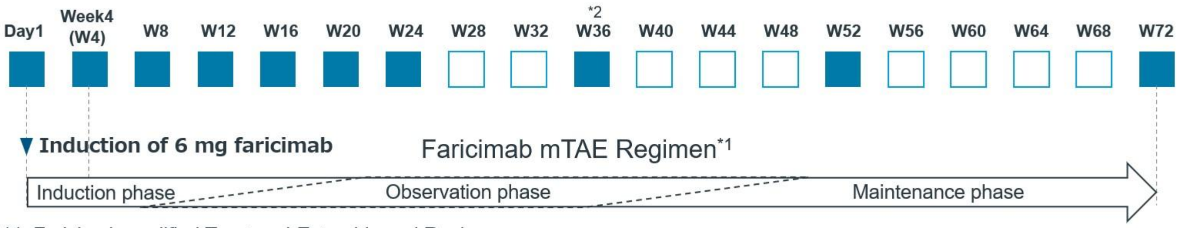

*1: Faricimab modified Treat-and-Extend-based Regimen

- Induction phase: All patients will receive one injection of faricimab on Day 1 and every 4 weeks after Week 4 until resolution of macular edema is observed (up to 6 injections).
- Observation phase: Faricimab will not be administered, and patients will visit every 4 weeks until disease activity is observed.
- Maintenance phase: The initial dosing interval during the maintenance phase will be the duration obtained by subtracting 4 weeks from duration of the observation phase. Dosing intervals during the maintenance phase will be adjusted by 4-week extension, reduction, or maintenance.

■ : Scheduled visit

At scheduled visits, the following are performed:

– Review of concomitant medication/therapy
– Review of adverse events
– Visual acuity test
– SD-OCT or SS-OCT
– OCT-A

*2 (W36): Only patients in the observation phase

III Planned enrollment

The sample size was set at 72 cases. Among them, the enrollment of patients with HRVO will be limited to 14 cases or fewer. See Section 3.5 “Rationale for the Target Sample Size”, for more details.

IV Patients

Patients who meet all of the inclusion criteria and do not meet any of the exclusion criteria below are included in the study.

(1) Inclusion Criteria

1) General Inclusion Criteria

① Willingness and the ability to provide signed informed consent

② Age ≥18 years at the time of signing informed consent

③ Ability and willingness to undertake all scheduled visits and assessments

2) Ocular Inclusion Criteria for Study Eye

In this study, only one eye will be assigned as the study eye. If both eyes can serve as the study eye, the study eye will be selected at the discretion of the investigator.

① Foveal center-involved macular edema secondary to CRVO or HRVO, diagnosed within 4 months before the screening visit based on spectral-domain optical coherence tomography (SD-OCT) or swept source OCT (SS-OCT) images
CRVO is defined as retinal hemorrhages, telangiectatic capillary bed, dilated venous system, or other biomicroscopic evidence of Retinal vein occlusion (RVO) (neovascularization or vitreous hemorrhages) in the entire retina, and HRVO as those in 2 quadrants of the retina drained by the affected vein.
* Rationale: Established in accordance with the COMINO study (NCT04740931).

② Naive to treatment of macular edema secondary to CRVO or HRVO (including IVT anti-VEGF injections and steroids)

③ Decimal visual acuity of 0.5 to 0.05, as assessed on the visual acuity test on Day 1 pre-dose

* Rationale: Decimal visual acuity of 0.5 to 0.05 was considered appropriate in accordance with the criterion in the COMINO study (73 to 19 letters on the ETDRS visual acuity test).

④ CST that meets either of the following at the screening visit:
  – ≥325 μm on Spectralis SD-OCT
  – ≥315 μm on Cirrus SD-OCT, Topcon SD-OCT, or other equivalent OCT

* Rationale: To perform assessments appropriately.

⑤ Sufficiently clear ocular media and adequate pupillary dilatation to allow acquisition of good quality retinal images to confirm diagnosis

* Rationale: To perform assessments appropriately.

(2) Exclusion Criteria

Patients who meet any of the following criteria will be excluded from the study.

1) General Exclusion Criteria

① Systemic treatment for suspected or active systemic infection on Day 1

② Stroke (cerebral vascular accident) or myocardial infarction within 6 months prior to Day 1

③ Uncontrolled blood pressure (defined as systolic >180 mmHg and/or diastolic >110 mmHg while a patient is at rest) on Day 1. If a patient's initial reading during the screening period exceeds these values, a second reading may be obtained later the same day or on another day during the screening period.

④ Active cancer within 12 months prior to screening except for appropriately treated carcinoma in situ of the cervix, non-melanoma skin carcinoma, and prostate cancer with a Gleason score of ≤6 and a stable prostate-specific antigen for >12 months.

⑤ Any significant disease or significant surgical procedure within 1 month prior to screening

⑥ Systemic steroids (e.g., oral or injected) within 1 month prior to screening

⑦ History or presence of other diseases, metabolic dysfunction, physical examination finding, or clinical laboratory finding giving reasonable suspicion of a condition that contraindicates the use of faricimab or that might affect interpretation of the results of the study or renders the patient at high risk for treatment complications in the opinion of the investigator

⑧ Pregnancy or breastfeeding

⑨ Women of childbearing potential * unless they agree to remain abstinent (refrain from heterosexual intercourse) or use acceptable contraceptive methods that result in a failure rate of <1% per year ** during the treatment period and for at least 3 months after the final dose of study treatment.

* A woman is considered to be of childbearing potential if she is postmenarcheal, has not reached a postmenopausal state (≥12 continuous months of amenorrhea with no identified cause other than menopause), and is not permanently infertile due to surgery (removal of ovaries, fallopian tubes, and/or uterus) or another cause as determined by the investigator (e.g., Mullerian agenesis). As per this rule, women who have had unilateral tubal ligation is considered to be of childbearing potential.

** Examples of contraceptive methods that result in a failure rate of <1% per year include bilateral tubal ligation, male sterilization, hormonal contraceptives that inhibit ovulation, hormone-releasing intrauterine devices, and copper intrauterine devices. The reliability of sexual abstinence should be evaluated in relation to the duration of the clinical trial and the preferred and usual lifestyle of the patient. Periodic abstinence (e.g., calendar,

ovulation, symptothermal, or post ovulation methods) and withdrawal are not adequate methods of contraception.

⑩ History of a severe allergic reaction or anaphylactic reaction to a biologic agent or known hypersensitivity to any component of faricimab, drugs used in the study procedures (including fluorescein), dilating drops, or any of the anesthetic and antimicrobial preparations used by a patient during the study

⑪ Participation in an ophthalmologic clinical trial that involves treatment with any drug (with the exception of vitamins and minerals) or device within 3 months prior to Day 1

⑫ Requirement for continuous use of any prohibited medications and treatments indicated below:

- Systemic anti-vascular endothelial growth factor (VEGF) therapy
- Systemic drugs known to cause macular edema (e.g., fingolimod, tamoxifen)
- IVT anti-VEGF agents (other than faricimab) in study eye
- IVT, periocular (subtenon), or chronic topical ocular corticosteroids in study eye
- Verteporfin (Visudyne®) therapy in study eye
- Administration of micropulse and focal or grid laser in study eye
- Other experimental therapies (except those comprising vitamins and minerals)

* Rationale:

① through ⑩ and ⑫: Due to safety concerns or effects on safety assessments.

⑪: This can prevent appropriate evaluation or affect efficacy assessments.

2) Ocular Exclusion Criteria for Study Eye

① History of macular edema secondary to CRVO or HRVO or persistent macular edema secondary to CRVO or HRVO diagnosed ≥4 months before screening

② History of retinal detachment or macular hole (Stage 3 or 4)

③ Any current ocular condition which, in the opinion of the investigator, is currently causing or could be expected to contribute to irreversible vision loss due to a cause other than macular edema secondary to CRVO or HRVO in the study eye (e.g., ischemic maculopathy, Irvine-Gass syndrome, foveal atrophy, foveal fibrosis, pigment abnormalities, dense subfoveal hard exudates, or other non-retinal conditions)

④ Tractional retinal detachment, full-thickness macular hole, vitreomacular traction, or epiretinal membrane involving the fovea or disrupting the macular architecture in the study eye

⑤ Diagnosis of moderate non-proliferative diabetic retinopathy, worse proliferative diabetic retinopathy, diabetic macular edema (DME), neovascular age-related macular degeneration (nAMD), geographic atrophy, or myopic choroidal neovascularization as assessed by the investigator

⑥ Active rubeosis, angle neovascularization, neovascular glaucoma

⑦ Any cataract surgery or treatment for complications of cataract surgery with yttrium-aluminum-garnet (YAG) laser capsulotomy within 3 months prior to Day 1

⑧ Any other intraocular surgery (e.g., pars plana vitrectomy, scleral buckle, glaucoma surgery, corneal transplant, or radiotherapy)

⑨ Macular laser (focal/grid) or panretinal photocoagulation (PRP) in the study eye performed prior to Day 1 or PRP scheduled within 3 months of the start of treatment on Day 1

⑩ Any prior intervention with photodynamic therapy (PDT), laser, transpupillary thermotherapy, or vitreoretinal surgery including sheathotomy

⑪ Any prior or current treatment for macular edema; macular neovascularization, including DME and nAMD; or vitreomacular-interface abnormalities, including IVT treatment (e.g., anti-VEGF medication, steroids, tissue plasminogen activator, C_3F_8, SF_6, air) or periocular injection

⑫ Prior periocular pharmacological or IVT treatment (including anti-VEGF medication) for other retinal diseases

* Rationale:

① through ⑫: These can prevent appropriate efficacy and safety assessments.

3) Exclusion Criteria for Both Eyes

① Any history of idiopathic or immune-mediated uveitis in either eye

② Active ocular inflammation or suspected or active ocular or periocular infection in either eye on Day 1

* Rationale:

①: This can prevent appropriate efficacy and safety assessments.

②: Due to safety concerns or effects on safety assessments.

V End of Study and Duration of Study

The study will end on the date when the summary of the Clinical Study Report is registered and released on the Japan Registry of Clinical Trials (jRCT).

VI Study Drug

Generic name: Faricimab (genetical recombination)

Brand name: VABYSMO solution for Intravitreal Injection 120 mg/mL

VII Schedule of Study Assessments

See Appendix 1.

VIII Statistical Methods

(1) Primary Analysis

1) Rationale for the Target Sample Size

Target sample size: 72 patients (of these, ≤14 patients with HRVO will be enrolled)

Primary endpoint: change from baseline in BCVA (logMAR) at Week 72.

Based on a clinical study of faricimab in macular edema secondary to CRVO or HRVO (COMINO study), assuming a true value of 16.9 letters and SD of 16.45 for the change from baseline in BCVA at Week 72 (primary endpoint), a sample size of 50 patients will provide at least 90% probability that the point estimate of the primary endpoint is no more than 3 letters below the true value. The dropout rate was 10% in the COMINO study; however, in this current study, given that the medication costs are borne by the patients and outpatient visits also pose a great burden on the patients, the dropout rate by Week 72 was anticipated to be 30%, and the sample size was determined to be 72 patients.

2) Primary Endpoint Analysis

The primary efficacy endpoint in this study is the change from baseline in BCVA at Week 72. BCVA will be expressed as a logMAR value that is converted from decimal visual acuity measured on Landolt ring chart at a distance of 5 m.

BCVA will be analyzed using a mixed effect model for repeated measure (MMRM). The model will include visit (categorical variable) and baseline BCVA (continuous variable) as fixed effects, and an unstructured covariance structure will be assumed for modeling within-patient errors. If the model does not converge, a compound symmetry or autoregressive (1) covariance structure will be used. Missing at random will be assumed for the missing data mechanism. Patients with complete withdrawal of consent including refusal to have their data used for research will be excluded from the evaluation.

(2) Interim Analysis

When the BCVA data at Week 24 after treatment initiation are obtained from all patients, an interim analysis will be performed for the purpose of presenting the results at academic conferences and other venues. Additionally, further interim analysis may be conducted as needed. Additional details will be provided in the SAP.

IX Contact information

Principal Investigator:	Department of Ophthalmology, Mie University Graduate School of Medicine<br>Mineo Kondo, Professor
Clinical Study Office:	Department of Ophthalmology, Mie University Graduate School of Medicine<br>Mineo Kondo, Professor<br>2-174 Edobashi, Tsu, Mie 514-8507, Japan<br>Tel: +81-59-232-1111 (main)
Collaborating Research Institute:	Chugai Pharmaceutical Co., Ltd.<br>2-1-1 Nihonbashi-Muromachi, Chuo-ku, Tokyo 103-8324, Japan<br>Tel: +81-3-3281-6611
Support Office:	IQVIA Services Japan G.K.<br>Keikyu First Building, 4-10-18 Takanawa, Minato-ku, Tokyo 108-0074, Japan<br>Tel: +81-3-6859-9500

List of Abbreviations and Definitions of Terms

Abbreviation	Definition
Ang-1	angiopoietin-1
Ang-2	angiopoietin-2
AUC	area under the curve
BCVA	best corrected visual acuity
BRVO	branch retinal vein occlusion
CFP	color fundus photography
CRB	certified review board
CRVO	central retinal vein occlusion
CST	central subfield thickness
eCRF	electronic case report form
DME	diabetic macular edema
EDC	electronic data capture
ETDRS	Early Treatment Diabetic Retinopathy Study
FA	fluorescein angiography
Fab	fragment antigen-binding
FAS	full analysis set
Fc	fragment crystallizable
FcRn	neonatal Fc receptor
HBV	hepatitis B virus
HCV	hepatitis C virus
HIV	human immunodeficiency virus
HRVO	hemispherical retinal vein occlusion
IgG	immunoglobulin G
IRF	intraretinal fluid
jRCT	Japan Registry of Clinical Trials
logMAR	logarithm of the minimum angle of resolution
LPS	layperson Summary
MMRM	mixed effect model for repeated measure
mTAE	modified Treat and Extend
nAMD	neovascular age-related macular degeneration
OCT	optical coherence tomography
OCT-A	optical coherence tomography-angiography
PDT	photodynamic therapy
PPS	Per-protocol set
PRN	pro re nata
PRP	panretinal photocoagulation
RVO	retinal vein occlusion
SAP	statistical analysis plan
SD	standard deviation
SD-OCT	spectral-domain optical coherence tomography
SRF	subretinal fluid

Abbreviation	Definition
SS-OCT	swept source optical coherence tomography
TAE	treat-and-extend
Tie-2	tyrosine kinase with immunoglobulin and epidermal growth factor homology domains-2
VEGF	vascular endothelial growth factor
VEGF-A	vascular endothelial growth factor A
YAG	Yttrium Aluminum Garnet

Term	Definition
Resolution of macular edema	Central subfield thickness (CST) <325 μm, as measured on Spectralis spectral-domain optical coherence tomography (SD-OCT), or CST <315 μm, as measured on Cirrus SD-OCT, Topcon SD-OCT, or an equivalent OCT (to be determined by the investigator using this criteria)
Disease activity	CST increase of ≥20% from the reference CST (to be determined by the investigator using this criteria)
Reference CST	The CST value when resolution of macular edema is first observed after initiation of faricimab, or the lowest CST value so far if macular edema does not resolve after 6 consecutive injections of faricimab
Induction phase	The period from the day of faricimab initiation until the day when resolution of macular edema is first observed, or until Week 20 if macular edema does not resolve
Observation phase	The period from the day when resolution of macular edema is first observed after initiation of faricimab until the day disease activity is observed
Maintenance phase	Faricimab maintenance dosing period after the induction phase/observation phase

1. Background

1.1 Background on the Disease under Study

RVO is an obstruction of a vein in the retina or the optic nerve, which limits normal blood flow to the retina and induces increased expression and secretion of cytokines, particularly vascular endothelial growth factor (VEGF), leading to conditions such as fundal hemorrhage, macular edema, and reduced visual acuity [1].

According to the site of occlusion, RVO can be classified as central RVO (CRVO), hemispherical RVO (HRVO), and branch RVO (BRVO) [2]; typically, CRVO, which affects the entire retina, is more extensive and severe compared to BRVO, which affects only a part (approximately one quarter) of the retina [3,4].

RVO affected 16.4 million people worldwide in 2008 and is the second most common sight-threatening retinal vascular disorder after diabetic retinopathy [5]. The prevalence of RVO in Japan, which was examined in a study in people aged 40 years or older in 1998, was reported to be 2.1%, including 2.0% with BRVO and 0.2% with CRVO [6].

Macular edema secondary to RVO is also a cause of vision loss. For the treatment of macular edema secondary to RVO, intravitreal (IVT) anti-VEGF injections are currently considered the treatment of choice [2], and macular lasers [7] and IVT steroid injections [8] are used in some patients. IVT anti-VEGF injections may improve the outcome and durability of treatment by targeting not only VEGF but also other disease pathways [9]. Angiopoietin-2 (Ang-2) competes with angiopoietin-1 (Ang-1) for binding with tyrosine kinase with immunoglobulin and epidermal growth factor homology domains-2 (Tie-2), and interferes with the vessel stabilizing effects of Ang-1/Tie signaling [10–12]. In addition, preclinical study findings suggest that in retinal disorders, Ang-2 plays a critical role in cytokine-induced vascular leakage [13]. Ang-2 and VEGF promote vessel instability, leading to disease progression including increased vascular leakage, inflammation, and neovascularization [12], and they are considered as key factors involved in the pathophysiology of macular edema secondary to RVO. Dual inhibition of these two pathways may enable a more sustained stability of the retinal vessels compared with vascular endothelial growth factor A (VEGF-A) inhibitor alone [14–16].

1.2 Background on Faricimab

Faricimab (genetical recombination) (hereafter referred to as faricimab) is a humanized bispecific immunoglobulin G (IgG)1 antibody discovered by F. Hoffmann-La Roche Ltd. (hereafter referred to as Roche) that selectively binds to VEGF-A and Ang-2. The VEGF-A-binding fragment antigen-binding (Fab) domain binds to VEGF-A with high affinity, and the Ang-2-binding Fab domain binds to Ang-2 with high affinity and selectivity. Because the fragment crystallizable (Fc) domain was engineered to eliminate binding to Fcγ receptors on effector cells and the neonatal Fc receptor (FcRn), the systemic exposure of faricimab following IVT injection may be reduced. The efficacy and safety of faricimab have been confirmed in clinical studies in patients with nAMD [17] and DME [18], and based on these study results, faricimab was approved for age-related macular degeneration with subfoveal choroidal neovascularization and DME in Japan in March 2022. In addition, based on the results of clinical studies in patients with BRVO, CRVO, and HRVO, faricimab was approved for an additional indication of macular edema secondary to RVO in March 2024 [1].

1.3 Rationale for Study Plan

International phase III studies of anti-VEGF agents for macular edema in RVO [19–26] have demonstrated marked vision improvement with 6 monthly injections, and IVT injection of anti-VEGF agents is considered the first-line treatment. Anti-VEGF agents usually require long-term administration, and various methods of administration, including fixed dosing, pro re nata (PRN) regimen, and treat-and-extend (TAE) regimen, have been studied to determine the optimal dosing interval. However, it has been found that in clinical practice, a high percentage of patients discontinue treatment during the long-term treatment period, and treatment outcomes are poorer compared with clinical study results [27]. Factors that decrease adherence to anti-VEGF treatment include significant financial and psychological burdens on patients due to frequent treatments [28]. The PRN regimen is used in the maintenance phase following 6 injections, and while vision can be maintained for 1 year or longer in BRVO [21,26], the improved vision tends to decrease in CRVO, which remains a challenge [23–26]. Therefore, studies are needed to investigate new dosing regimens that are less burdensome to patients and factors related to their effects.

This study will examine a modified TAE (mTAE) regimen of faricimab. In the mTAE regimen, there is an observation phase before the traditional TAE regimen, allowing the TAE regimen to be started after determining each patient’s interval of recurrence. If this dosing regimen can extend dosing intervals while maintaining the vision improvement achieved, it may help close the current clinical gap.

In clinical trials of faricimab for macular edema secondary to BRVO, CRVO, and HRVO (BALATON and COMINO trials) [29], patients received 6 injections administered every 4 weeks as in studies using other anti-VEGF medication for macular edema in RVO [19–26], and non-inferiority of faricimab compared with aflibercept, which was used in the control arm, was demonstrated in the primary endpoint of vision improvement at Week 24. Furthermore, during the maintenance phase after Week 24, vision improvement was maintained up to Week 72 not only in BRVO but also in CRVO and HRVO following the TAE regimen [30].

2. Objectives and Endpoints

2.1 Objectives

To evaluate the efficacy and extension of dosing intervals of faricimab, as well as to explore the factors related to efficacy and dosing intervals using a dosing regimen that is workable in clinical practice (mTAE) in treatment-naive patients with macular edema secondary to CRVO or HRVO.

2.2 Endpoints

See below for efficacy assessments:

Section 2.2.1 “Primary Endpoint”, Section 2.2.2 “Secondary Endpoints”, and Section 2.2.3 “Exploratory Endpoints”.

See Section 2.2.4 “Safety Endpoints” for safety assessments.

See List of Abbreviations and Definitions of Terms for definitions of “induction phase,” “observation phase,” and “maintenance phase” of this study, as well as for definitions of “resolution of macular edema,” “disease activity,” and “reference CST.”

2.2.1 Primary Endpoint

- Change from baseline in BCVA at Week 72
  BCVA will be measured in decimal visual acuity at each measurement time point and converted to logarithm of the minimum angle of resolution (logMAR) to calculate the change in BCVA.

2.2.2 Secondary Endpoints

The following items will be evaluated at time points (specified in the SAP) for each endpoint up to Week 72:

- BCVA and change from baseline in BCVA (logMAR)
- Proportion of patients with a ≥0.3 logMAR improvement from baseline
- Proportion of patients without a ≥0.3 logMAR worsening from baseline
- CST and change from baseline in CST
- Proportion of patients for each dosing interval of faricimab
- Mean number of faricimab injections

The following items will be evaluated at Week 72:

- Number of days of the observation phase after induction of faricimab
- Proportion of patients who did not receive additional doses of faricimab during the observation phase after induction of faricimab

The following items will be evaluated at time points (specified in the SAP) for each endpoint up to Week 72 by the presence or absence of retinal ischemia at baseline. Retinal ischemia at baseline is defined as ≥10 disc areas of capillary occlusion on ETDRS 7-field fundus photography (or an equivalent range), and all others are defined as non-retinal ischemia.

- BCVA and change from baseline in BCVA
- CST and change from baseline in CST
- Proportion of patients for each dosing interval of faricimab
- Mean number of faricimab injections

2.2.3 Exploratory Endpoints

The following items will be evaluated at time points specified for each endpoint up to Week 72:

- Proportion of patients with resolution of macular edema
- Proportion of patients with absence of IRF, proportion of patients with absence of SRF, and proportion of patients with absence of both
- Fluid volumes (IRF and SRF) and their change from baseline
- Proportion of patients with absence of retinal ischemia (assessed by FA)
- The macular and total retinal areas of ischemic non-perfusion (capillary loss) and their change from baseline (assessed by FA)
- Proportion of patients who transitioned from non-ischemic CRVO to ischemic CRVO (assessed by FA)
- Area of vascular leakage in the macula and in the total retinal area and its change from baseline (assessed by FA)
- Proportion of patients with absence of vascular leakage (assessed by FA)

- Change from baseline in vascular density of the superficial, deep, and whole capillary plexuses (assessed by OCT-angiography [OCT-A])
- Relationships between baseline parameters and change from baseline in BCVA, change from baseline in CST, number of injections, dosing interval, and other efficacy parameters*
- Exploration of factors affecting the dosing interval at Week 72 *
- Relationships between variation of CST up to Week 24 (SD of each patient based on CST values from Week 4 to Week 24) and BCVA, dosing interval, and other efficacy parameters *
- Comparison of parameters at baseline and Week 72 of patients who received multiple injections versus patients who received only one injection of faricimab during the induction phase *
- Number of days to achieve the best BCVA (highest decimal visual acuity value) after initiation of faricimab
- Number of days to achieve the best CST (lowest CST value) after initiation of faricimab
- Area under the curve (AUC) of BCVA from Day 1 to Week 24
- Proportion of loss of peak vision (0.1, 0.2, and ≥0.3 logMAR worsening from best BCVA) and exploration of contributing demographic factors *
- Incidence of new epimacular membranes in patients with absence of epimacular membranes at baseline

* Factors to be analyzed and the methods of analysis will be specified in the SAP.

2.2.4 Safety Endpoints

- Incidence and severity of ocular adverse events
- Incidence and severity of non-ocular adverse events

3. Overview of the Study

3.1 Study Type

This is an interventional, unmasked, single-arm, multicenter clinical study that controls the use and extent of tests, medications, and other diagnostic or therapeutic medical acts for research purposes. This study is a “specified clinical trial” under the Clinical Trials Act.

The study will be conducted in compliance with the Declaration of Helsinki (Japan Medical Association translation),* the Clinical Trials Act (Act No. 16 of 2017),** the Enforcement Regulations of the Clinical Trials Act (Ministry of Health, Labour and Welfare [MHLW] Ordinance No. 17 of 2018),** and this protocol.

* https://dl.med.or.jp/dl-med/wma/helsinki2013j.pdf

** https://www.mhlw.go.jp/stf/seisakunitsuite/bunya/0000163417.html

3.2 Study Design

This study is an unmasked, single-arm, multicenter, prospective interventional study to investigate the efficacy and safety of faricimab dosing regimen (mTAE regimen) in treatment-naive patients with macular edema secondary to CRVO or HRVO. This study is a “specified clinical trial” under the Clinical Trials Act.

Patients who take part in this study will receive IVT injections of 6.0 mg faricimab. The duration of participation is 72 weeks; all patients will receive faricimab on Day 1, after which patients will

receive faricimab or undergo observation according to each criteria of the induction phase, observation phase, and maintenance phase. Procedures performed during each phase are described in Section 5.2 “Protocol Treatment”. Study visits will occur every 4 weeks from Day 1 through Week 24, then after Week 24, patients will come to the study site at a frequency depending on the dosing interval determined according to the mTAE regimen, with scheduled visits at Week 36 (only patients in the observation phase), Week 52, and Week 72.

The study schedule diagram is shown in Figure 3.2-1.

Figure 3.2-1 Study schedule diagram.

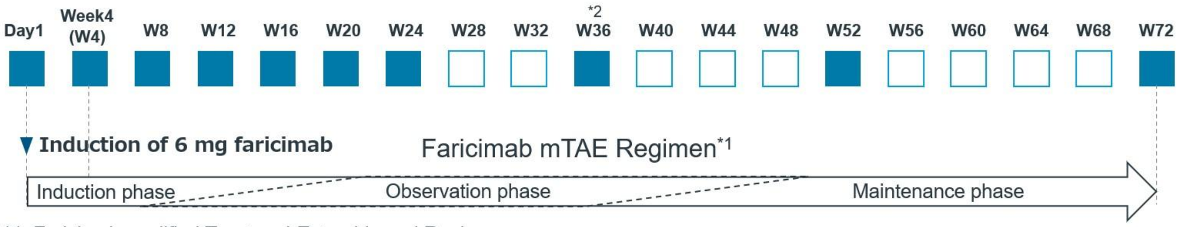

*1: Faricimab modified Treat-and-Extend-based Regimen

- Induction phase: All patients will receive one injection of faricimab on Day 1 and every 4 weeks after Week 4 until resolution of macular edema is observed (up to 6 injections).
- Observation phase: Faricimab will not be administered, and patients will visit every 4 weeks until disease activity is observed.
- Maintenance phase: The initial dosing interval during the maintenance phase will be the duration obtained by subtracting 4 weeks from duration of the observation phase. Dosing intervals during the maintenance phase will be adjusted by 4-week extension, reduction, or maintenance.

■ : Scheduled visit

At scheduled visits, the following are performed:

– Review of concomitant medication/therapy
– Review of adverse events
– Visual acuity test
– SD-OCT or SS-OCT
– OCT-A

*2 (W36): Only patients in the observation phase

3.3 End of Study and Duration of Study

The study will end on the date when the summary of the Clinical Study Report is registered and released on the Japan Registry of Clinical Trials (jRCT).

The planned enrollment period and planned follow-up period are set as follows, considering the time needed for application to the Certified Review Board (CRB).

- Planned enrollment period: 1 year and 6 months from the day of the first patient enrollment after the jRCT release date
- Planned follow-up period: 72 weeks from the day of the last patient enrollment after the jRCT release date
- Report preparation period: 1 year from the end of the follow-up period
- Planned duration of study: 4 years from the jRCT release date

3.4 Rationale for Study Design

In international phase III studies of anti-VEGF treatment for macular edema in RVO [21,24–26], patients were given 6 consecutive injections every 4 weeks and demonstrated marked improvement in vision, and this has become the standard of care. However, previous international phase III studies have used the PRN regimen during the maintenance phase following 6 injections, and while vision can be maintained for 1 year or longer in BRVO [21,26], the vision improvement tend to decrease in CRVO [23–26], which remains a challenge.

In clinical trials of faricimab for macular edema secondary to BRVO, CRVO, and HRVO (BALATON and COMINO trials) [29], patients received 6 injections administered every 4 weeks as

in studies using other anti-VEGF medication for macular edema in RVO [19–26], and non-inferiority of faricimab, compared with aflibercept in the control arm, was demonstrated in the primary endpoint of vision improvement at Week 24. Furthermore, during the maintenance phase after Week 24, vision improvement was maintained up to Week 72 not only in BRVO but also in CRVO and HRVO following the TAE regimen [29].

On the other hand, the mean number of faricimab injections in the COMINO study was 11 in 72 weeks, which is more than twice as many as in clinical practice [31]. Regarding dosing intervals, the COMINO study had a lower proportion of patients with a dosing interval of 12 weeks or longer, compared with results of faricimab in other diseases (nAMD, DME) [17,18]. One possible reason for these differences is that the COMINO study used a stricter regimen (once the dosing interval was reduced it could not be re-extended) compared with studies in other diseases. While burden of treatment (such as drug costs, frequency of visits) is a major factor that decreases treatment adherence [32], insufficient vision improvement in clinical practice also remains a challenge; therefore, studies in CRVO and HRVO are needed to investigate faricimab regimens that are usable in clinical practice and can reduce the dosing frequency, while maintaining vision improvement comparable to those in international phase III studies.

The mTAE regimen used in this study does not specify the number of injections to be administered during the induction phase; patients will receive up to 6 injections of faricimab until resolution of macular edema, and after the resolution, they will enter the observation phase and undergo observation. Then, if patients meet the re-dosing criteria, treatment will be resumed immediately, and they will enter the maintenance phase with an initial dosing interval that is the same duration as the observation phase. With this mTAE regimen, the number of injections during the induction phase and dosing intervals during the maintenance phase can be adjusted to the patient’s disease status, which may prevent overdosage of anti-VEGF medication, reduce burden for patients, and contribute to long-term maintenance of vision.

3.5 Rationale for the Target Sample Size

Target sample size: 72 patients (of these, ≤14 patients with HRVO will be enrolled)

Based on a clinical study of faricimab in macular edema secondary to CRVO or HRVO (COMINO study), assuming a true value of 16.9 letters and SD of 16.45 for the change from baseline in BCVA at Week 72 (primary endpoint), a sample size of 50 patients will provide at least 90% probability that the point estimate of the primary endpoint is no more than 3 letters below the true value. The dropout rate was 10% in the COMINO study [29]; however, in this current study, given that the medication costs are borne by the patients and outpatient visits also pose a great burden on the patients, the dropout rate by Week 72 was anticipated to be 30%, and the sample size was determined to be 72 patients. In addition, in order to align patient demographics with the COMINO study, an upper limit was set for the number of HRVO patients to be enrolled. Based on the proportion of HRVO patients in the COMINO study (17.5% in the intention-to-treat population, 16.3% in the Japan subpopulation), the maximum number of HRVO patients was set to 14 in the current study so that the proportion of HRVO patients does not exceed 20% of all patients.

3.6 Significance of Study

The emergence of anti-VEGF agents has greatly advanced the treatment of RVO. On the other hand, since frequent IVT injections of anti-VEGF agents are burdensome for patients, the PRN treatment, in which treatment is given again at worsening of disease, is the mainstream treatment in clinical practice. However, inadequate treatment may lead to gradual progression of the retinal disorder and may result in irreversible vision loss. If this study can demonstrate the efficacy and safety of treatment with the faricimab mTAE regimen, it can provide a new treatment option that enables patients to maintain visual function with less burden, which may help to close the current clinical gap.

3.7 Potential Benefits and Disadvantages Associated with Study Participation

Because all patients in the study will receive treatment with faricimab for 72 weeks, they can expect improvement in their macular edema secondary to CRVO or HRVO, but no greater benefit compared with usual care. The results of the study may lead to advances in the treatment of macular edema secondary to CRVO or HRVO in the future as an academic achievement.

On the other hand, participation in the study may involve increased number of visits and tests compared to usual care, and patients may have to bear more costs of tests; therefore, patients will receive payments for reducing patient burden per visit for observation and testing visits for which there is no scheduled treatment (see Section 10.10.1 “Costs of Treatment”). As when receiving faricimab in usual care, patients may experience adverse reactions listed in the faricimab package insert. A list of adverse reactions associated with faricimab is shown in Table 3.7-1, and potential risks from tests and procedures performed in the study are shown in Table 3.7-2.

Table 3.7-1 Adverse Reactions of Faricimab (as of March 2024) [1]

**Significant adverse reactions**	
Eye disorders<br>• Intraocular inflammation (e.g., uveitis, vitritis) (1.2%)<br>• Retinal pigment epithelial tear (0.2%)<br>• Endophthalmitis (incidence unknown)<br>• Rhegmatogenous retinal detachment (incidence unknown)<br>• Retinal tear (incidence unknown)<br>Stroke (0.3%)	
**Other adverse reactions**	
Eye disorders (<1%)<br>• Intraocular pressure increased<br>• Vitreous floaters<br>• Ocular hypertension<br>• Corneal abrasion<br>• Eye pain<br>• Ocular discomfort	• Conjunctival hemorrhage<br>• Cataract<br>• Vitreous detachment<br>• Ocular hyperemia<br>• Blurred vision<br>• Visual acuity reduced

Table 3.7-2 Potential Risks from Tests and Procedures

Test or procedure	Potential risks
IVT injection	• Bacterial infection may occur at the site of injection. Antibiotic eye drops that are used to prevent infection may cause eye irritation, itching, swelling, or hyperemia.<br>• Use of anticoagulants (e.g., aspirin or similar drugs [such as warfarin]) may predispose to intraocular hemorrhage associated with faricimab injection.
Intraocular pressure (IOP) measurement (if the measurement involves touching the cornea with a tonometer)	• Topical ocular anesthetics may be used.
FA	• Before obtaining images, fluorescein will be injected into the patient's arm. The needle puncture may cause discomfort.<br>• The injection of the dye can cause inflammation in the vein or cause redness or swelling at the injection site.<br>• The main adverse reactions of fluorescein include nausea and vomiting; however, occasional allergic reactions, syncope, dyspnea, or shock may occur. Fluorescein may cause the skin and urine to turn yellow, but this will disappear in about a day.
Use of povidone iodine	• Povidone iodine, which will be used by the investigator who will be administering the faricimab injections to prevent infection, may cause a brief sensation of burning, soreness, or irritation in the eye.
Use of mydriatics	• Mydriatics may cause stinging in the eye.<br>• Patients may experience blurred vision for a period of time after the use of mydriatics. Patients should be advised not to drive or use machinery until this symptom has resolved.
Use of antibiotic eye drops	• Antibiotic eye drops may be used before and after the faricimab injection to prevent infection at the discretion of the investigator.<br>• Use of antibiotic eye drops may cause irritation, itching, swelling, or redness of the eye.

For adverse events, the inclusion criteria (Section 4.1.1), exclusion criteria (Section 4.1.2), and treatment plans and criteria for adjusting treatment (Section 5) are established to minimize their risks, and a structure is in place to ensure that necessary measures are taken when serious or unexpected adverse events occur.

4. Materials and Methods

4.1 Patients

Patients who meet all of the inclusion criteria and do not meet any of the exclusion criteria below are included in the study.

4.1.1 Inclusion Criteria

1) General Inclusion Criteria

① Willingness and the ability to provide signed informed consent

② Age ≥18 years at the time of signing informed consent

③ Ability and willingness to undertake all scheduled visits and assessments

2) Ocular Inclusion Criteria for Study Eye

In this study, only one eye will be assigned as the study eye. If both eyes can serve as the study eye, the study eye will be selected at the discretion of the investigator.

① Foveal center-involved macular edema secondary to CRVO or HRVO, diagnosed within 4 months before the screening visit based on SD-OCT or SS-OCT images
CRVO is defined as retinal hemorrhages, telangiectatic capillary bed, dilated venous system, or other biomicroscopic evidence of RVO (neovascularization or vitreous hemorrhages) in the entire retina, and HRVO as those in 2 quadrants of the retina drained by the affected vein.
* Rationale: Established in accordance with the COMINO study.

② Naive to treatment of macular edema secondary to CRVO or HRVO (including IVT anti-VEGF injections and steroids)

③ Decimal visual acuity of 0.5 to 0.05, as assessed on the visual acuity test on Day 1 pre-dose
* Rationale: Decimal visual acuity of 0.5 to 0.05 was considered appropriate in accordance with the criterion in the COMINO study (73 to 19 letters on the ETDRS visual acuity test).

④ CST that meets either of the following at the screening visit:
- ≥325 μm on Spectralis SD-OCT
- ≥315 μm on Cirrus SD-OCT, Topcon SD-OCT, or other equivalent OCT

* Rationale: To perform assessments appropriately.

⑤ Sufficiently clear ocular media and adequate pupillary dilatation to allow acquisition of good quality retinal images to confirm diagnosis
* Rationale: To perform assessments appropriately.

4.1.2 Exclusion Criteria

Patients who meet any of the following criteria will be excluded from the study.

1) General Exclusion Criteria

① Systemic treatment for suspected or active systemic infection on Day 1

② Stroke (cerebral vascular accident) or myocardial infarction within 6 months prior to Day 1

③ Uncontrolled blood pressure (defined as systolic >180 mmHg and/or diastolic >110 mmHg while a patient is at rest) on Day 1. If a patient's initial reading during the screening period exceeds these values, a second reading may be obtained later the same day or on another day during the screening period.

④ Active cancer within 12 months prior to screening except for appropriately treated carcinoma in situ of the cervix, non-melanoma skin carcinoma, and prostate cancer with a Gleason score of ≤6 and a stable prostate-specific antigen for >12 months.

⑤ Any significant disease or significant surgical procedure within 1 month prior to screening

⑥ Systemic steroids (e.g., oral or injected) within 1 month prior to screening

⑦ History or presence of other diseases, metabolic dysfunction, physical examination finding, or clinical laboratory finding giving reasonable suspicion of a condition that contraindicates the use of faricimab or that might affect interpretation of the results of the study or renders the patient at high risk for treatment complications in the opinion of the investigator

⑧ Pregnancy or breastfeeding

⑨ Women of childbearing potential * unless they agree to remain abstinent (refrain from heterosexual intercourse) or use acceptable contraceptive methods that result in a failure rate of <1% per year ** during the treatment period and for at least 3 months after the final dose of study treatment.

* A woman is considered to be of childbearing potential if she is postmenarcheal, has not reached a postmenopausal state (≥12 continuous months of amenorrhea with no identified cause other than menopause), and is not permanently infertile due to surgery (removal of ovaries, fallopian tubes, and/or uterus) or another cause as determined by the investigator (e.g., Mullerian agenesis). As per this rule, women who have had unilateral tubal ligation is considered to be of childbearing potential.

** Examples of contraceptive methods that result in a failure rate of <1% per year include bilateral tubal ligation, male sterilization, hormonal contraceptives that inhibit ovulation, hormone-releasing intrauterine devices, and copper intrauterine devices. The reliability of sexual abstinence should be evaluated in relation to the duration of the clinical trial and the preferred and usual lifestyle of the patient. Periodic abstinence (e.g., calendar, ovulation, symptothermal, or post ovulation methods) and withdrawal are not adequate methods of contraception.

⑩ History of a severe allergic reaction or anaphylactic reaction to a biologic agent or known hypersensitivity to any component of faricimab, drugs used in the study procedures (including fluorescein), dilating drops, or any of the anesthetic and antimicrobial preparations used by a patient during the study

⑪ Participation in an ophthalmologic clinical trial that involves treatment with any drug (with the exception of vitamins and minerals) or device within 3 months prior to Day 1

⑫ Requirement for continuous use of any prohibited medications and treatments indicated below:

- Systemic anti-VEGF therapy
- Systemic drugs known to cause macular edema (e.g., fingolimod, tamoxifen)
- IVT anti-VEGF agents (other than faricimab) in study eye
- IVT, periocular (subtenon), or chronic topical ocular corticosteroids in study eye
- Verteporfin (Visudyne®) therapy in study eye
- Administration of micropulse and focal or grid laser in study eye
- Other experimental therapies (except those comprising vitamins and minerals)

* Rationale:

① through ⑩ and ⑫: Due to safety concerns or effects on safety assessments.

⑪: This can prevent appropriate evaluation or affect efficacy assessments.

2) Ocular Exclusion Criteria for Study Eye

① History of macular edema secondary to CRVO or HRVO or persistent macular edema secondary to CRVO or HRVO diagnosed ≥4 months before screening
② History of retinal detachment or macular hole (Stage 3 or 4)
③ Any current ocular condition which, in the opinion of the investigator, is currently causing or could be expected to contribute to irreversible vision loss due to a cause other than macular edema secondary to CRVO or HRVO in the study eye (e.g., ischemic maculopathy, Irvine-Gass syndrome, foveal atrophy, foveal fibrosis, pigment abnormalities, dense subfoveal hard exudates, or other non-retinal conditions)
④ Tractional retinal detachment, full-thickness macular hole, vitreomacular traction, or epiretinal membrane involving the fovea or disrupting the macular architecture in the study eye
⑤ Diagnosis of moderate non-proliferative diabetic retinopathy, worse proliferative diabetic retinopathy, DME, nAMD, geographic atrophy, or myopic choroidal neovascularization as assessed by the investigator
⑥ Active rubeosis, angle neovascularization, neovascular glaucoma
⑦ Any cataract surgery or treatment for complications of cataract surgery with YAG laser capsulotomy within 3 months prior to Day 1
⑧ Any other intraocular surgery (e.g., pars plana vitrectomy, scleral buckle, glaucoma surgery, corneal transplant, or radiotherapy)
⑨ Macular laser (focal/grid) or panretinal photocoagulation (PRP) in the study eye performed prior to Day 1 or PRP scheduled within 3 months of the start of treatment on Day 1
⑩ Any prior intervention with PDT, laser, transpupillary thermotherapy, or vitreoretinal surgery including sheathotomy
⑪ Any prior or current treatment for macular edema; macular neovascularization, including DME and nAMD; or vitreomacular-interface abnormalities, including IVT treatment (e.g., anti-VEGF medication, steroids, tissue plasminogen activator, C_3F_8, SF_6, air) or periocular injection
⑫ Prior periocular pharmacological or IVT treatment (including anti-VEGF medication) for other retinal diseases

* Rationale:
① through ⑫: These can prevent appropriate efficacy and safety assessments.

3) Exclusion Criteria for Both Eyes

① Any history of idiopathic or immune-mediated uveitis in either eye
② Active ocular inflammation or suspected or active ocular or periocular infection in either eye on Day 1

* Rationale:
①: This can prevent appropriate efficacy and safety assessments.
②: Due to safety concerns or effects on safety assessments.

4.2 Enrollment

4.2.1 Enrollment Procedures

Patients will be enrolled in the study using an electronic data capture (EDC) system.

The investigator is required to obtain approval from the CRB. After approval, the investigator will obtain a user ID and password needed to log in to the EDC system from the Support Office and will enroll patients.

The investigator will explain to each study patient about the study, and if the patient gives written informed consent, the investigator will confirm that the patient meets all of the entry criteria and none of the exclusion criteria, and will then access the EDC system via the Internet.

Enrollment is available 24 hours a day, except during system checks and maintenance for troubleshooting. The investigator will follow the directions of the EDC system and enter the necessary items to enroll patients.

Name of EDC system:	Zelta
URL:	https://zeltatrials.com/ng/login/credential
Contact information:	zelta.support@merative.com

4.2.2 Issuance and Notification of Enrollment Results

Entry into electronic case report forms (eCRFs) is possible only for patients whose eligibility is confirmed based on the entries on the enrollment screen.

4.2.3 Enrollment in Multiple Studies

Co-enrollment in another study is permitted if the investigator judges that there will be no problem with co-enrollment upon review of the protocol of the other study. However, co-enrollment in a clinical trial for regulatory approval in Japan is not permitted.

4.2.4 Notes on Enrollment

Enrollment after treatment initiation is not permitted without exception. Enrollment is performed by accessing the EDC system URL listed in Section 4.2.1 “Enrollment Procedures”. Eligibility will be confirmed on the screen of the EDC system.

All data required at enrollment are mandatory, and they must be true and correct. If a false enrollment is discovered after enrollment, this will be handled as a serious violation.

If data entry is insufficient, the enrollment will not be accepted until all data are filled in.

Once a patient is enrolled, his or her enrollment will not be canceled (deleted from the database) even when the patient requests complete withdrawal of consent including refusal to have his or her data used for research (if a patient requests complete withdrawal of consent including refusal to have his or her data used for research, the patient’s data will be excluded from analyses by handling data; however, if the database is locked for analysis report at the time of the request, consent may not be withdrawn).

If erroneous or duplicate enrollment is discovered, the Support Office should be contacted immediately. For duplicate enrollments, the initial enrollment information (enrollment number) should be used in all cases.

4.2.5 Procedures for Closing Enrollment

When the planned enrollment for the study is expected to be reached, the Support Office will send an e-mail informing the investigators of the enrollment status. The Support Office will notify the study sites that enrollment will be achieved and of notes on future patient enrollment (after the

planned enrollment has been achieved, study sites will no longer inform new patients about the study).

4.3 Schedule of Study Assessments

The schedule of activities in this study is provided in Appendix 1. The investigator will perform observations and tests according to the study schedule. The screening and Day 1 visits may occur as a combined visit if all assessments are completed and evaluated on the same day or within 2 business days. When screening and the Day 1 visit are completed on the same day, the assessments listed for both visits should be conducted only once.

4.3.1 Informed Consent Forms and Screening Log

Before a study patient participates in the study, the investigator must fully inform the patient about the study using the information sheet and obtain his or her voluntary consent in writing. If the study patient cannot give written consent due to illness or any other reason (if the patient is unable to read the document because of visual impairment but is able to understand its contents by oral explanation, or if the patient is unable to write his or her signature due to limb problems but is able to read and understand the document), a witness should be present during the informed consent process. The witness will sign and date the Informed Consent Form, certifying that the potential subject of the study has understood the study and freely given his or her consent. The witness must not be a person involved in the study. Regardless of enrollment in the study, the subject's Informed Consent Form will be maintained at the study site, and the patient will receive a copy of the Informed Consent Form.

The investigator should confirm the eligibility of study patients before enrollment in the study. Results of tests and assessments performed as part of routine care within 14 days before Day 1, even prior to obtaining informed consent, may be used, and such tests and assessments do not need to be repeated for screening. In addition, the study drug should be given within 28 days after obtaining informed consent.

4.3.2 Medical and Surgical History and Patient Demographics

During the screening period, history of clinically significant diseases, surgeries, and medications (all prescription drugs taken more than 7 days before the start of treatment) will be recorded in the eCRF.

Adverse events that occur after obtaining informed consent but prior to the first dose of the study drug will be recorded in the eCRF as past medical history or concomitant disease. Clinically significant diseases that have been cured within 6 months before from initiation of faricimab treatment will be recorded as past medical history, and diseases that are not cured at the time of initiation of faricimab treatment will be recorded as concomitant disease.

Sex, age, and race will be recorded as patient demographics in the eCRF.

4.3.3 Blood Pressure

Systolic and diastolic blood pressure will be measured while the patient is in a sitting position according to the study schedule (Appendix 1).

4.3.4 Ocular Tests

The following ocular tests will be performed according to the study schedule (Appendix 1).

4.3.4.1 Intraocular Pressure (IOP) Measurement

IOP will be measured before dilation for ocular tests and recorded in the eCRF. If IOP before dilation is ≥30 mmHg, administration of dilating drops and study drug should be stopped. Persistently increased IOP will be recorded in the eCRF as an adverse event.

The method of IOP measurement used for a patient must remain consistent throughout the study.

4.3.4.2 Refraction Test

Refraction tests of the study eye will be performed. The tests will be performed before dilation. Spherical power, cylindrical power, and cylinder axis at screening will be recorded in the eCRF. Any clinically significant refractive errors (myopia, hyperopia, astigmatism, and anisometropia) will be recorded in the eCRF as concurrent disease.

4.3.4.3 Axial Length Test

Axial length of the study eye will be measured before dilation for ocular tests and recorded in the eCRF.

4.3.4.4 Visual Acuity Test

Visual acuity tests will be performed before dilation. Corrected distance (5 m) visual acuity will be measured on Landolt ring chart, and decimal visual acuity will be recorded in the eCRF.

BCVA will be expressed in logMAR, which is converted from decimal visual acuity using the following formula:

logMAR = log (1/d), (d = decimal visual acuity)

Decimal visual acuity below 0.02 will be assessed by finger-counting, hand motion, or light perception tests.

4.3.4.5 Fundoscopy

FA

Following separate written procedures, FA of the study eye will be performed, in principle, after pupillary dilation. Images will be analyzed (e.g., microaneurysms, area of ischemic non-perfusion, or vascular leakage area calculations) at the Image Analysis Institution. The imaging procedures, sending and receiving of imaging data, and analysis of images are described in the written procedures.

Color Fundus Photography (CFP)

Following separate written procedures, color fundus photography images of the study eye will be taken, in principle, after pupillary dilation. Images will be analyzed at the Image Analysis Institution. The imaging procedures, sending and receiving of imaging data, and analysis of images are described in the written procedures.

4.3.5 Optical Coherence Tomography (OCT)

SD-OCT or SS-OCT

Following separate written procedures, OCT scans of the study eye will be performed, in principle, after pupillary dilation to measure CST and to determine the presence of IRF, SRF, and epimacular membranes, and the results will be recorded in the eCRF. Images (IRF and SRF volumes) will be analyzed at the Image Analysis Institution. The imaging procedures, sending and receiving of imaging data, analysis of images, and reporting of analysis results are described in the written procedures.

OCT-A

OCT-A will be performed at each visit. OCT-A of the study eye will be performed with a scanning range of 6 mm × 6 mm, in principle, after pupillary dilation, and vascular density of the superficial, deep, and whole capillary plexuses will be recorded in the eCRF.

4.3.6 Unscheduled Visits

If a patient visits the study site before a scheduled study visit (± visit window) (unscheduled visit), the patient is required to undergo assessments that are to be performed on all scheduled visits (review of concomitant medication/therapy, adverse events, visual acuity test, IOP measurement, SD-OCT or SS-OCT, and OCT-A) and undergoes other necessary assessments as determined by the investigator.

If disease activity (a CST increase of ≥20% from the reference CST) is observed at an unscheduled visit, the need for treatment on that day and subsequent dosing interval will be determined according to the previous treatment status (see Section 5.2 "Protocol Treatment").

4.3.7 Patient Discontinuation from Study

Patients have the right to voluntarily withdraw from the study at any time for any reason. In addition, the investigator has the right to withdraw a patient from the study at any time. Reasons for withdrawal from the study may include the following:

- Patient withdrawal of consent
- Patient meets the exclusion criteria
- Any medical condition that, in the opinion of the investigator, makes it difficult for the patient to continue in the study for safety reasons
- Investigator determines that discontinuation of faricimab or the study is in the best interest of the patient

If a patient voluntarily withdraws from the study, the investigator or study collaborator should record the date of and reason for discontinuation for each study patient. In addition, as long as the study patient agrees, an effort should be made to ensure the safety of the subject by performing protocol-specified reviews, observations, and tests. Not all assessments at discontinuation are required to be conducted; these should be conducted upon confirming the study patient's will.

If the investigator determines that it is difficult for the patient to continue in the study due to the occurrence of adverse events, the investigator should discontinue the study and subsequently follow up on the patient. If an illness or infection is unresolved at the time of study end or discontinuation, follow-up should be continued until it recovers or resolves.

4.3.8 Site Discontinuation

If the investigator terminates or suspends the study, he or she should promptly notify the site manager and provide a detailed written explanation. The site manager should promptly notify the Principal Investigator in writing and provide a detailed written explanation.

The Principal Investigator will report the site's discontinuation during the continuation review by the CRB and modify the information registered in the database managed by the MHLW (jRCT*).

* The database managed by the MHLW that specified in Article 24 paragraph (1) of the Enforcement Regulations of the Clinical Trials Act (Japan Registry of Clinical Trials)

URL: https://jrct.niph.go.jp/

5. Treatment Plans and Criteria for Adjusting Treatment

5.1 Study Drug

Generic name	Faricimab (genetical recombination)
Brand name	VABYSMO solution for Intravitreal Injection 120 mg/mL
Route of administration	Intravitreal
Dosage and administration in the package insert [1] (Macular edema secondary to retinal vein occlusion)	One dose is 6.0 mg (0.05 mL) of faricimab (genetical recombination) administered by intravitreal injection. The dosing interval should be 4 weeks or longer.
Marketing authorization holder	Chugai Pharmaceutical Co., Ltd.

5.2 Protocol Treatment

Patients who take part in this study will receive IVT injections of 6.0 mg faricimab. The duration of participation is 72 weeks; all patients will receive faricimab on Day 1, and subsequent injections will be administered according to each criteria of the induction phase, observation phase, and maintenance phase. Study visits will occur every 4 weeks from Day 1 through Week 24, then after Week 24, patients will come to the study site at a frequency depending on the dosing interval determined according to the mTAE regimen, with scheduled visits at Week 36 (only patients in the observation phase), Week 52, and Week 72.

In this study, the terms "resolution of macular edema," "disease activity," and "reference CST" are defined as follows:

Resolution of macular edema	CST <325 μm, as measured on Spectralis spectral-domain optical coherence tomography (SD-OCT), or CST <315 μm, as measured on Cirrus SD-OCT, Topcon SD-OCT, or an equivalent OCT (to be determined by the investigator using this criteria)
Disease activity	CST increase of ≥20% from the reference CST
Reference CST	The CST value when resolution of macular edema is first observed after initiation of faricimab, or the lowest CST value so far if macular edema does not resolve after 6 consecutive injections of faricimab

1) Induction Phase

The induction phase will be the period from faricimab initiation until first observation of resolution of macular edema (or until Week 20 if resolution of macular edema is not observed).

All patients will receive one injection of faricimab on Day 1 and every 4 weeks thereafter. After Week 4, if resolution of macular edema is observed before treatment, faricimab will not be administered and the patient will enter the observation phase. The CST value at this visit will be the reference CST.

The maximum number of faricimab injections to be administered during the induction phase is 6. If macular edema does not resolve after 6 consecutive injections by Week 20, the patient will enter the maintenance phase without undergoing the observation phase with the next scheduled injection at Week 24. In this case, the lowest CST value obtained from Day 1 through Week 20 will be the reference CST.

Of note, resolution of macular edema will be determined by the investigator based on the protocol-defined criteria; even in cases where these criteria are not met, the investigator may consider that macular edema has resolved, with recording of the reason for such judgment.

2) Observation Phase

The observation phase is defined as the period from the day of the visit when resolution of macular edema is observed until the day disease activity is observed again.

During the observation phase, faricimab will not be administered, and patients will be monitored every 4 weeks until Week 24 and at scheduled visits at Week 36, Week 52, and Week 72 thereafter, unless disease activity is observed. If disease activity is observed at a visit, faricimab will be administered on that day or within the visit window (-7 to +14 days of the scheduled visit), and the patient will enter the maintenance phase.

Even if a patient does not meet the disease activity criteria, if he or she has a significant decrease in visual acuity compared to the last visit (e.g., decimal visual acuity of 0.6 to 0.3) and has edema that requires treatment as judged by the investigator, he or she will be considered to have disease activity and administration of faricimab will be permitted. On the other hand, even if a patient meets the disease activity criteria, if he or she has no subjective symptoms and the change is considered to be of no clinical significance by the investigator, he or she may be judged to have no disease activity. The investigator should record the reasons for such judgments.

Patients will be monitored at unscheduled visits in between scheduled visits if deemed necessary by the investigator. If disease activity is observed at an unscheduled visit, the patient will be given faricimab and enter the maintenance phase.

3) Maintenance Phase

The initial dosing interval during the maintenance phase will be the same duration as the observation phase (or 4 weeks if the patient entered the maintenance phase without undergoing the observation phase). Dosing intervals during the maintenance phase will be adjusted every 4 weeks according to the following adjustment criteria. Dosing intervals will be adjusted by ±4 weeks, with a minimum dosing interval of 4 weeks and no limits on the maximum dosing interval. If the investigator deems that the patient needs an adjustment that differs from this adjustment criteria, the dosing interval will be adjusted as such, with recording of the reason for the judgment.

CST percent change from reference CST (%)	Dosing interval adjustment
<+10%	Extend interval (+4 weeks)
≥+10% to <+20%	Maintain interval (±0 weeks)
≥+20%	Reduce interval (-4 weeks)

Even if a patient does not meet the disease activity criteria, if he or she has a significant decrease in visual acuity compared to the last visit (e.g., decimal visual acuity of 0.6 to 0.3) and has edema that requires treatment as judged by the investigator, he or she will be considered to have disease activity and dosing interval will be reduced. On the other hand, even if a patient meets the disease activity criteria, if he or she has no symptoms and the change is considered to be of no clinical significance by the investigator, he or she may be considered to have no disease activity with recording of the reason for such judgment, and the dosing interval may be extended or maintained.

IVT injections of faricimab must be administered appropriately, referring to the latest package insert [19] and the Appropriate Use Guide [33].

5.3 Protocol Treatment Interruption/Discontinuation Criteria

If any of the following criteria are met, treatment with the study drug will be interrupted or discontinued. The reason for treatment interruption/discontinuation should be recorded on the appropriate eCRF page, and treatment interruption/discontinuation for adverse events should be recorded on the Adverse Event eCRF.

Table 5.3-1 Dose Interruption and Treatment Discontinuation Criteria

Event	Criteria
Intraocular inflammation	• Interrupt study treatment if intraocular inflammation (e.g., iritis, iridocyclitis or vitritis) is ≥2+ in the study eye.<br>• Study treatment may be resumed subsequently as determined by the investigator.
Cataract surgery in the study eye	• Interrupt study treatment after cataract surgery in the study eye.<br>• Study treatment may be resumed no earlier than 28 days after an uncomplicated cataract surgery and no evidence of post-operational inflammation at that time. For cataract surgery with complications, study treatment may be permitted as determined by the investigator.
BCVA decrease	• Interrupt study treatment if there is a study treatment-related decrease in BCVA of ≥0.6 logMAR in the study eye compared with the last assessment of BCVA prior to the most recent treatment.<br>• Study treatment may be permitted subsequently, as determined by the investigator.
Elevated IOP	• Interrupt study treatment if pre-treatment IOP in the study eye is ≥30 mmHg.<br>• Study treatment may be permitted when IOP has been lowered to <30 mmHg, either spontaneously or by treatment, as determined by the investigator.
Rhegmatogenous retinal break	• Interrupt study treatment if a retinal break is present in the study eye.<br>• Study treatment may be resumed no earlier than 28 days after successful laser retinopexy, as determined by the investigator.
Rhegmatogenous retinal detachment or macular hole	• Interrupt study treatment if rhegmatogenous retinal detachment or Stage 3 or 4 macular hole occurs in the study eye.<br>• Study treatment may be permitted, as determined by the investigator.
Active or suspected infection	• Interrupt study treatment if active or suspected ocular or periocular infections are present (e.g., infectious conjunctivitis, infectious keratitis, infectious scleritis, or endophthalmitis) in either eye or if the patient requires treatment for an active systemic infection.<br>• Study treatment may be permitted, as determined by the investigator.
Use of prohibited medications or therapies	• Discontinue from the study if prohibited medications or therapies listed in Section 5.4.4 “Prohibited Medications and Therapies” are used.

If treatment is interrupted for any reason, the procedure below should be followed when resuming treatment:

- Treatment will be given at the time of resumption, and subsequent treatment schedule will be determined according to the dosing interval prior to interruption.

5.4 Concomitant and Supportive Therapies

5.4.1 Specified Concomitant and Supportive Therapies

Not applicable.

5.4.2 Concomitant and Supportive Therapies That Are Recommended/Not Recommended

Not applicable.

5.4.3 Concomitant and Supportive Therapies That Are Permitted

The following are some common therapies that are permitted to be used concomitantly with faricimab in this study:

- Onset of ocular hypertension or glaucoma in the study eye during a patient's study participation should be treated as clinically indicated.
- Onset of cataract or posterior capsular opacification in either eye during a patient's study participation may be treated as clinically indicated. Dose interruption criteria (see Section 5.3 "Protocol Treatment Interruption/Discontinuation Criteria", Table 5.3-1) may apply with cataract surgery
- Short-term use of topical ocular corticosteroids after cataract surgery, YAG laser capsulotomy, peripheral iridotomy, argon/selective laser trabeculoplasty, or ocular allergic conditions
- Complete, partial, or local PRP in the study eye may be performed if deemed necessary by the investigator for the treatment of ischemic RVO or new neovascularization in the periphery. If performed, it should be recorded as concomitant therapy, and the reason should also be recorded.
- Sight-threatening vitreous hemorrhage or retinal detachment in the study eye may be treated with vitrectomy. These conditions should be recorded as adverse events and also as concomitant therapy. Treatment should be interrupted and may be resumed based on the patient's condition. The patient should complete study visits as scheduled.
- Non-study eye treatment with anti-VEGF therapy (At the discretion of the investigator, patients may receive treatment with anti-VEGF therapy licensed for use in the non-study eye. However, treatment of both eyes on the same day should be avoided in initial treatment; safety in one eye should be fully evaluated before treatment is given to the other eye.)

5.4.4 Prohibited Medications and Therapies

The following medications and treatments are prohibited from use during a patient's study participation. Patients will be discontinued from the study to receive these therapies:

- Systemic anti-VEGF therapy
- Systemic drugs known to cause macular edema (e.g., fingolimod, tamoxifen)
- IVT anti-VEGF agents (other than faricimab) in study eye

- IVT, periocular (subtenon), steroid implants, or chronic topical ocular corticosteroids in study eye
- Verteporfin (Visudyne®) therapy in study eye
- Administration of micropulse and focal or grid laser in study eye
- Other experimental therapies (except those comprising vitamins and minerals)

5.5 Subsequent Therapy

No provisions are made concerning subsequent therapy.

6. Safety Assessments

6.1 Anticipated Adverse Drug Events of Individual Drugs

Refer to the latest package inserts for details on individual drugs used in the study. (See: Japan Pharmaceuticals and Medical Devices Agency [PMDA] https://www.pmda.go.jp/PmdaSearch/iyakuSearch/)

6.2 Definition of Adverse Events

6.2.1 Definition of Adverse Events

An adverse event is defined as any unfavorable and unintended injury or illness or its sign (including an abnormal laboratory finding) that occurs in a patient, whether or not considered related to the study. In this study, all adverse events that occur after initiation of treatment with faricimab until the final study visit will be assessed. The outcome of an adverse event should be followed until it resolves, abates, or stabilizes.

6.2.2 Definition of Illness

An "illness" in this document is defined as, among adverse events, any disease, disability, death, infection, abnormal laboratory finding, or symptom that is suspected to be caused by the conduct of the study. In this study, it refers to a case where there is a reasonable possibility that the adverse event is related to the study drug or to a protocol-specified test.

"Infection" refers to cases in which contamination of the biological product with pathogenic agents from biological materials is suspected. In addition, positive virus markers, including hepatitis B virus (HBV), hepatitis C virus (HCV), and human immunodeficiency virus (HIV), are subject to infectious disease reporting.

6.2.3 Grade of Adverse Events

The seriousness of adverse events is categorized in two groups: 0. Non-serious and 1. Serious, and will be assessed for each adverse event.

In accordance with the Act on Securing Quality, Efficacy and Safety of Pharmaceuticals, Medical Devices, Regenerative and Cellular Therapy Products, Gene Therapy Products, and Cosmetics, an event that meets any of the following criteria will be handled as a serious adverse event in this study:

Definition of a serious adverse event

1) Is fatal
2) Is life threatening
3) Requires or prolongs inpatient hospitalization for treatment
4) Results in persistent or significant disability/incapacity
5) Is a congenital anomaly/birth defect
6) Other events or reactions that are considered medically significant

6.2.4 Assessment of Severity of Adverse Events

The criteria for assessing adverse event severity are provided below.

Table 6.2-1 Adverse Event Severity Grading Scale

Severity	Description
Mild	Discomfort noticed, but no disruption of normal daily activity
Moderate	Discomfort sufficient to reduce or affect normal daily activity
Severe	Incapacitating with inability to work or to perform normal daily activity

* Regardless of severity, some events may also meet seriousness criteria. Refer to Section 6.2.3 “Grade of Adverse Events” for serious adverse events.

6.2.5 Causality Assessment Criteria

The criteria for assessing causality of adverse events are as follows.

Table 6.2-2 Criteria for Assessing Causality of Adverse Events to Treatment

Causality	Assessment criteria
Yes	There is a reasonable possibility that the adverse event is related to faricimab.<br>The adverse event meets any of the following:<br>• The event recurs after reintroduction of the drug<br>• The event is adequately predictable from the pharmacological actions of the drug, or a causal relationship with the drug or other drugs in the same class has been established<br>• Administration of faricimab and the onset of the event can be explained temporally<br>• The event resolves upon discontinuation of faricimab alone<br>• The event cannot be explained by factors such as underlying disease, concomitant disease, preexisting condition, or concomitant treatment
No	The causal relationship between the adverse event and faricimab can be ruled out.<br>The adverse event meets any of the following:<br>• There is no temporal relationship with administration of faricimab<br>• The event can be reasonably explained by other factors (e.g., underlying disease, concomitant disease, preexisting condition, concomitant treatment)

7. Reporting of Device Malfunctions That May Lead to Adverse Events or Injuries

7.1 Reporting Period for Device Malfunctions That May Lead to Adverse Events or Injuries

After initiation of the first dose of faricimab in this study, all device malfunctions that may lead to adverse events or injuries, regardless of relationship to the study drug, that developed until the final visit, date consent is withdrawn, or date lost to follow-up will be reported and recorded in the eCRF .

7.2 Management of All Device Malfunctions That May Lead to Adverse Events or Injuries

Investigators will seek information on adverse events at each patient contact and update the information in the eCRF. All device malfunctions that may lead to adverse events or injuries, whether reported by the patient or noted by study personnel at the site, will be recorded in the patient's medical record.

When the Support Office learns of a device malfunction that may lead to adverse events or injuries from the site, the Support Office should report this to Chugai Pharmaceutical Co., Ltd. according to predetermined procedures.

Chugai Pharmaceutical Co., Ltd. will evaluate the information on device malfunctions that may lead to adverse events or injuries that has been obtained and, when necessary, report this information to the PMDA and affiliate companies contracted to send and receive safety information. If needed, measures will be taken including notifying the sites of matters that need to be known. In addition, investigators should cooperate as much as possible in the additional investigations on queries from Chugai Pharmaceutical Co. Ltd.

The Principal Investigator will consider the urgency, importance, and impact of the reported information on device malfunctions that may lead to adverse events or injuries that has been obtained from the site and determine how to manage them, seeking the opinion of the Study Executive Committee as necessary.

7.3 Management of Illnesses and Infections

The Principal Investigator and the investigator will report any information obtained on an illness or infection following the procedures 1) through 6) below.

If a subinvestigator obtains the information, he or she should inform the investigator immediately to take similar actions; however, if it is not possible to contact the investigator, the subinvestigator should perform the duties of the investigator on his or her behalf.

1) If the investigator obtains information on adverse events that are serious illnesses, serious infections, or non-serious unexpected infections, he or she should report this to the site manager immediately, then fill out the designated items on Uniform Form 8 Drug Illness Report (hereafter referred to as Form 8) to the extent possible within 10 days, and contact the Principal Investigator, Clinical Study Office, and the safety management personnel of the Support Office via email. Any new information after the initial report should be added on Form 8 and reported to the Principal Investigator/Clinical Study Office/Support Office at any time.
2) After obtaining Form 8, the Principal Investigator/Clinical Study Office/Support Office should submit this form to the manager of the Principal Investigator's site and CRB within the

reporting time limit specified below. However, urgent cases, such as when information on an unexpected death is obtained, should be immediately reported to the manager of the Principal Investigator's site. This reporting can be done by telephone or orally.

	Condition other than infection		Infection	
	Unexpected*	Expected**	Unexpected*	Expected**
Death	15 days	15 days	15 days	15 days
Serious	15 days	30 days	15 days	15 days
Non-serious	Periodic reporting	Periodic reporting	15 days	Periodic reporting

* The investigator will consider the illness as "unexpected" if it is not listed in any of the following documents and is unpredictable: (1) study protocol or Informed Consent Form, (2) significant adverse reactions and other adverse reactions sections of the package insert(s) of drug(s) used in this study, (3) Appropriate Use Guide of drug(s) used in this study [33].

** If the illness is listed in any of the documents (1), (2), or (3) listed above, it will be considered as "expected."

3) After reporting to the Clinical Study Office in step 2), the Principal Investigator/Clinical Study Office/Support Office will notify the investigators at all sites that reporting has been made to the CRB and provide them with this information. In addition, a copy of Form 8 that has been submitted should be provided to the Collaborating Research Institute without delay after CRB reporting.
4) The investigators at all sites will report the provided information to the manager of their site.
5) If the Principal Investigator/Clinical Study Office/Support Office obtains the review results of the information from the CRB, they will report the results to the site manager, as well as to the investigators at all sites.
6) The investigators at all sites will report the CRB review results to the manager of their site.

* The latest version of Form 8 is available on the MHLW website; the latest version should be used for reporting. (MHLW website: http://www.mhlw.go.jp/stf/seisakunitsuite/bunya/0000163417.html)

Upon receiving an illness report, the CRB should, if deemed necessary, provide its opinion to the investigator or the Principal Investigator on measures to be taken to identify the cause or prevent recurrence of the illness that has been reported. If the CRB provides its opinion to the Principal Investigator or the investigator after receiving the illness report, the Principal Investigator or the investigator must respect this opinion and take necessary measures.

7.4 Follow-up of Patients after Adverse Events

The investigator will provide the best available treatment for any adverse events (including adverse events relating to laboratory values) that occur after initiation of treatment with faricimab in this study. If the investigator determines that it is difficult for the patient to continue in the study due to the occurrence of adverse events, the investigator will discontinue the study and subsequently follow up on the patient.

If an illness or infection is unresolved at the time of study end or discontinuation, follow-up should be continued until it recovers or resolves.

During the study period, any changes in the outcome of study patients (with dates) should be documented in the source document and the eCRF.

7.5 Pregnancies in Female Patients

Female patients will be instructed through the Informed Consent Form to immediately inform the investigator if they become pregnant during the study or within 3 months after the last dose of faricimab. After learning of the pregnancy, the investigator should report it to the Clinical Study Office using the Reporting Form for Pregnancy (Attachment 3) and handle it appropriately. Pregnancy in female patients should not be recorded in the eCRF.

The health status of the newborn child should be recorded on the Reporting Form for Pregnancy (Attachment 3) and submitted to the Clinical Study Office. In addition, information about the child's health status at 6 and 12 months after birth should be recorded on the Reporting Form for Newborn Child (Attachment 4) and submitted to the Clinical Study Office/Support Office.

7.6 Reporting of Overdose/Medication Error/Drug Abuse/Drug Misuse

If an event that corresponds to faricimab overdose or medication error is identified, the investigator should report it to the Clinical Study Office/Support Office using the Reporting Form for Overdose/Medication Error/Drug Abuse/Drug Misuse (Attachment 2).

8. Statistical Considerations and Analysis Plan

Details of the statistical analyses will be specified in the SAP that will be created separately before the database is locked. If there are changes to be made to the SAP, it will be amended as needed according to Section 10.8 "Change Application Procedures".

8.1 Planned Enrollment and Planned Overall Duration of Study

Based on the rationale described in Section 3.5 "Rationale for the Target Sample Size", 72 patients will be enrolled in the study.

The planned enrollment period and planned follow-up period are set as follows, considering the time needed for application to the CRB:

- Planned enrollment: 72 patients
- Planned enrollment period: 1 year and 6 months from the day of the first patient enrollment
- Planned follow-up period: 72 weeks from the day of the last patient enrollment
- Report preparation period: 1 year from the end of the follow-up period
- Planned duration of study: 4 years from the jRCT release date

8.2 Analysis Populations

The analysis populations are defined as follows:

- Full analysis set (FAS): All patients who receive at least one injection of faricimab and who have post-treatment efficacy data
- Per-protocol population (PPS): All patients in the FAS who do not have a major protocol violation
- Safety-evaluable population: All patients who receive at least one injection of faricimab

8.3 Primary Endpoint Analysis

The primary efficacy endpoint in this study is the change from baseline in BCVA at Week 72.

The consistency with the change from baseline in BCVA at Week 72 in the COMINO study will be evaluated on an exploratory basis. No statistical hypothesis is formulated and no tests are performed in the consistency assessment.

BCVA will be expressed as a logMAR value that is converted from decimal visual acuity measured on Landolt ring chart at a distance of 5 m.

logMAR = log (1/d), (d = decimal visual acuity)

If decimal visual acuity is below 0.02, visual acuity will be assessed by finger-counting, hand motion, or light perception tests and converted to decimal visual acuity and logMAR using the table below.

	Decimal visual acuity	LogMAR
Finger-counting (50 cm/finger-counting)	0.01	2.0
Hand motion	0.005	2.3
Light perception	0.002	2.7
No light perception	0.001	3.0

BCVA will be analyzed using a MMRM. The model will include visit (categorical variable) and baseline BCVA (continuous variable) as fixed effects, and an unstructured covariance structure will be assumed for modeling within-patient errors. If the model does not converge, a compound symmetry or autoregressive (1) covariance structure will be used. Missing at random will be assumed for the missing data mechanism. Patients with complete withdrawal of consent including refusal to have their data used for research will be excluded from the evaluation.

In addition, as a secondary analysis, approximate ETDRS letter scores converted from logMAR units using the formula below [34] will be analyzed with the same methods as the primary endpoint.

ETDRS letters = 85 - 50 x logMAR = 85 - 50 x log (1/d), (d = decimal visual acuity)

Decimal visual acuity below 0.02 (assessed by finger-counting, hand motion, or light perception tests) will be calculated as 0 ETDRS letters.

8.4 Secondary Endpoint Analysis

Means, medians, ranges, interquartile range, and SDs will be calculated for continuous variables.

Absolute and relative frequencies will be calculated for categorical variables.

The primary analysis will be repeated on subpopulations (specified in the SAP).

Additional details regarding the plan for the secondary endpoint analyses will be provided in the SAP.

8.5 Exploratory Endpoint Analysis

Means, medians, ranges, interquartile range, and SDs will be calculated for continuous variables.

Absolute and relative frequencies will be calculated for categorical variables.

Scatter diagrams of parameters will be created.

Additional details regarding the plan for the exploratory endpoint analyses will be provided in the SAP.

8.6 Safety Endpoint Analysis

The number and proportion of patients will be tabulated.

Additional details regarding the plan for the safety endpoint analyses will be provided in the SAP.

8.7 Interim Analysis

Some analyses, including the primary analysis, will be performed when the BCVA data at Week 24 after treatment initiation are obtained from all patients.

8.8 End of Study

The Principal Investigator should create a primary endpoint report, Clinical Study Report, and its summary and, after consulting the CRB, inform the study personnel that the study has ended, and will submit documents reporting the study conclusion and the summary of the results to the manager of the Principal Investigator's site. Summaries will be submitted to the Minister of Health, Labour and Welfare and released on the jRCT managed by the MHLW.

The study will end on the date when the summary of the Clinical Study Report is registered and released on the jRCT.

9. Data Collection and Management

9.1 Data Quality Assurance

The data manager of the Support Office will be responsible for data management of this study, including quality checking of the data. Data entered will be collected via EDC through use of eCRFs. Sites will be responsible for data entry into the EDC system. In the event of discrepant data, the data manager will request data clarification from the sites, which the sites will resolve electronically in the EDC system.

eCRFs and correction documentation will be maintained in the EDC system's audit trail. System backups for data stored by the Support Office and records retention for the study data will be consistent with the Support Office's standard procedures.

9.2 Electronic Case Report Forms (eCRFs)

eCRFs of all patients enrolled should be completed and submitted.

Data will be entered in eCRFs using the EDC system described in Section 4.2 "Enrollment". The investigator or the study collaborator at the site will be issued an account to access the eCRFs. eCRFs will be electronically submitted to and stored in the database and should be handled in accordance with separate written procedures.

All eCRFs should be completed by site staff who have been issued an account. eCRFs should be reviewed and electronically signed by the investigator.

9.2.1 eCRF Items

See Appendix 2.

9.3 Source Data Documentation

9.3.1 Data Entered into eCRFs Only

The eCRF is considered the source document for the following items, which are entered into eCRFs only:

1) Treatment status: Reason for treatment interruption/discontinuation, reason for change in treatment schedule
2) Adverse events: Severity and seriousness of adverse event, causality assessment, outcome, and outcome date
3) Concomitant medication and concomitant therapy: Purpose of concomitant medication or concomitant therapy
4) Study discontinuation: Reason for discontinuation

9.3.2 Source Documents

Source documents are documents (paper or electronic) in which patient data are recorded. They include, but are not limited to, hospital records, clinical and office charts, laboratory notes, memoranda, pharmacy dispensing records, recorded data from automated instruments, copies of transcriptions that are certified after verification as being accurate and complete, microfiche, photographic negatives, microfilm or magnetic media, X-rays, patient files, and records kept at pharmacies, laboratories, and medico-technical departments involved in a clinical trial.

Source documents that are required to verify the validity and completeness of data entered into the eCRFs must not be obliterated or destroyed and must be retained per the policy for retention of records.

The investigators and study sites must provide direct access to all clinical records including source documents for study-related monitoring (audits) and CRB and regulatory authority inspections.

9.3.3 Use of Computerized Systems

When clinical observations are entered directly into a study site’s electronic medical record system in lieu of original hardcopy records, the electronic record can serve as the source document.

An computerized data collection system allows preservation of the original entry of data (original data). If original data are modified, the system should maintain a viewable audit trail that shows the original data as well as the reason for the change, name of the person making the change, and date and time of the change.

9.4 Handling and Retention of Records, Information, and Samples

9.4.1 Study Site

Records relating to patient’s consent, basic data (e.g., laboratory data) for the preparation of reports (eCRFs), CRB approval letters, and records created by the institution will be properly retained by the investigator according to the study site’s standard operating procedures. The retention period will last until the day 5 years after the termination or completion of the entire study or the day after the period specified by each study site ends, whichever comes later. After the end of the retention period, the documents will be properly destroyed according to study site rules.

9.4.2 Collaborating Research Institute

Study-related documents and records to be retained by the Collaborating Research Institute will be retained by the Collaborating Research Institute according to the Standard Operating Procedures on the retention of clinical study records and storage and destruction of information, which are prepared and managed by Chugai Pharmaceutical Co., Ltd. The retention period will last until the day 5 years after the termination or completion of the entire study. After the end of the retention period, the documents and records will be properly destroyed according to the Standard Operating Procedures, prepared and managed by Chugai Pharmaceutical Co., Ltd.

9.4.3 Support Office

Information entered into the eCRFs during the study will be stored on the server of the Support Office, which is responsible for managing the EDC system. Data entered into the EDC system will be analyzed after database lock.

Study-related documents and records will be properly retained by the Support Office according to the Support Office's standard procedures. The retention period will last until the day 5 years after the termination or completion of the entire study.

9.4.4 Image Analysis Institution

Study-related documents and records will be properly retained according to the Image Analysis Center's standard procedures. The retention period will last until the day 5 years after the termination or completion of the entire study.

9.5 Retention and Destruction of Information Provision Records

In this study, information will not be provided to research institutes other than the study organization; however, the Support Office will collect information as part of its contracted study-related duties. Therefore, in order to maintain traceability, the Support Office will retain the following records pertaining to provision of information. These records will be destroyed as described in Section 9.4.3 "Support Office".

Name of institution receiving information	See Section 15 "Study Administrative Structure, Support Office"
Name of investigator at site receiving information	See Section 15 "Study Administrative Structure, Support Office"
Name of institution providing information	See Attachment 1 "List of Study Sites and Investigators"
Name of investigator at site providing information	See Attachment 1 "List of Study Sites and Investigators"
Items of information	See Section 9.2.1 "eCRF Items"
Process of obtaining information	Information was obtained at study sites according to the protocol.
Patient's name, etc.	Retention of patient's name, etc. will be substituted by proper retention of the Consent Forms by study sites.

10. Ethical Considerations

10.1 Protection of Patients

All researchers involved in the study will conduct the study in compliance with the Declaration of Helsinki (Japan Medical Association translation),* the Clinical Trials Act (Act No. 16 of 2017),** the Enforcement Regulations of the Clinical Trials Act (MHLW Ordinance No. 17 of 2018),** and related notifications and this protocol.

* https://dl.med.or.jp/dl-med/wma/helsinki2013j.pdf

** https://www.mhlw.go.jp/stf/seisakunitsuite/bunya/0000163417.html

10.2 Informed Consent

10.2.1 Informed Consent

Before a study patient participates in the study, the investigator must inform the patient of the following information using the Information Sheet. The investigator should provide the study patient with an opportunity to ask questions or discuss concerns, give adequate time for consideration, and confirm that the patient fully understands the contents of the study before asking the patient to participate in the study. If the study patient agrees to participate in the study, the investigator will obtain the patient's signature on the Consent Form. Regardless of enrollment in the study, the patient's Informed Consent Form will be maintained at the study site, and the patient will receive a copy of the Informed Consent Form. If the patient is unable to read the document because of visual impairment but is able to understand its contents by oral explanation, or if the patient is unable to write his or her signature due to limb problems but is able to read and understand the document, a witness should be present during the informed consent process. In this case, the witness will sign and date the Informed Consent Form, certifying that the potential study patient of the study has understood the study and freely given his or her consent. The witness must not be a person involved in the study.

1) Name of the specified clinical trial to be conducted and the fact that the conduct of the study has been approved by the site manager and that the implementation plan has been submitted to the Minister of Health, Labour and Welfare
2) Name of the study site and the name, title, and contact details of the investigator (if the specified clinical trial is conducted as a multicenter study, the name and title of the Principal Investigator, names of other study sites, and names and titles of investigators at other study sites should be included)
3) Reason the patient was selected as a subject of the specified clinical trial
4) Potential benefits and disadvantages of the specified clinical trial
5) That the patient is free to refuse participation in the specified clinical trial
6) Matters concerning withdrawal of consent
7) That there will be no penalty for refusing to participate or withdrawing consent to participate in the specified clinical trial
8) How information from the specified clinical trial will be publicized
9) That the subject of the specified clinical trial may obtain or view the protocol and other documents related to the conduct of the specified clinical trial upon request, and how to obtain or view them
10) Matters concerning the protection of personal information about the subjects of the specified clinical trial

11) How samples, etc. will be retained and destroyed
12) Status of study-related conflicts of interest of the study institution (e.g., study funding sources) and study-related conflicts of interest of the researchers (e.g., personal income)
13) Structure of handling complaints and inquiries
14) Matters concerning the costs of conducting the specified clinical trial
15) Availability of other treatments, their contents, and comparison with the expected benefits and disadvantages of other treatments
16) Matters concerning compensation and provision of medical care for injuries resulting from the conduct of the specified clinical trial
17) Matters reviewed by the CRB, which is responsible for reviewing and providing opinions on the specified clinical trial, and other matters concerning the CRB on the specified clinical trial
18) Other matters necessary for the conduct of the specified clinical trial (e.g., purpose, significance, methods, and duration of the study, and secondary use of data)

10.2.2 Withdrawal of Consent

Withdrawal of consent means to withdraw consent to participate in the study and should be distinguished from refusal to continue treatment (see 1 below). If a patient expresses his or her wish to withdraw consent, it should be made clear whether item 2 or 3 below is applicable, and this should be entered in the Discontinuation/Completion Report on the eCRF.

In the case of withdrawal of consent (see 2 below), all subsequent requests for protocol follow-up should be stopped. In the case of complete withdrawal of consent (see 3 below), the patient's data will be made unavailable for data review, correction, output, and analysis on confirming that the patient is completely withdrawing consent.

The procedures for stopping follow-up requests to the patient and for removing the patient's data from the analysis set will be determined separately.

1) Refusal to continue treatment: The patient refuses to continue further treatment (but continues with follow-up).
2) Withdrawal of consent: The patient withdraws consent to participate in the study and does not permit any subsequent protocol treatment or follow-up. Data obtained before withdrawal of consent may be used in the study.
3) Complete withdrawal of consent: The patient withdraws consent to participate in the study and does not permit any data obtained from the time of study entry, including information collected at enrollment, to be used in the study. However, if the database is already locked for analysis report, consent may not be withdrawn.

10.3 Handling of Inquiries from Patients

The investigator will set up a study help desk for patients and relevant others and provide information in the Information Sheet on how it can be contacted. In principle, any study-related inquiries from patients or their families received after enrollment should be handled by the researcher (investigator) at the study site of the patient. If it is unclear how to handle an inquiry, the matter will be addressed in consultation with the Principal Investigator and the Study Executive Committee, if necessary, through the Support Office.

10.4 Protection of Personal Information and Identification of Patients

Patient names will not be provided from the study site to the Support Office personnel, except to monitoring personnel.

Enrollment numbers issued at the time of enrollment and patient identification codes will be used to identify and make queries about patients. Patient names and other information that a third party could use to directly identify a patient without unauthorized access to a study site employee or database will not be registered in the study database. If the study results are published, information that personally identifies patients will not be included.

The study site should use personal information with due consideration given to the confidentiality of patient information and with proper management according to study site rules, in order to minimize the risk of data leakage.

10.4.1 Purpose of Use of Personal Information, Information to be Used, and Methods for Use

1) Purpose of Use

Following the philosophy of "Providing the best treatment to more patients," personal patient information will be used with the objectives of "Studying certain patients not only during treatment but also over longer periods after the completion of treatment to obtain correct results of the clinical study and properly managing the information obtained."

In addition, patients should be informed that information from this study will be provided to Roche (Switzerland), which is the developer of the study drug, as well as to its affiliates, collaborators, and partners, and that appropriate measures will be taken according to the laws and regulations of the recipient's country* if information is provided, and consent should be obtained.

* Reference information: Personal Information Protection Commission website, Laws in other countries https://www.ppc.go.jp/enforcement/infoprovision/laws/

2) Information Used

The following information, which is the minimum necessary information to identify and make queries about patients, will be used:

- Patient identification number (assigned by the study site), year and month of birth

The study sites will not inform the data center of patient names or any personal information other than the above; and if any personal information is accidentally sent, regardless of the recording media used, it will be destroyed or made illegible through appropriate processing such as masking before storage.

3) Methods for Use

Personal information and healthcare information of patients will be collected when the investigator or study collaborator of the study site enters it in the eCRF. Personal information will not be shared via email. Medical records and other data may be directly viewed during central monitoring and site visits carried out on the basis of data entries in the eCRFs; however, even if personal information is accessed for such monitoring and audit purposes, the information is protected from being leaked through contracts and confidentiality obligations.

10.4.2 Secondary Use of Data

Samples and data obtained in this study may be used secondarily in Japan or overseas, if approved after consultation with the Principal Investigator and the Collaborating Research Institute and after review and approval from the CRB. In such cases, appropriate actions will be taken in compliance with relevant laws and regulations in Japan and other countries. The possibility of secondary use of data should be stated in the Information Sheet, and consent from study patients should be obtained in advance. In order to comply with the General Data Protection Regulation (GDPR) and data privacy requirements of each country, when the Principal Investigator or Collaborating Research Institute provides clinical study data to third parties including those in other countries (e.g., other medical institutions or pharmaceutical companies), clinical study data will be managed in a manner that does not identify individuals, and access to only anonymized patient data will be given to secondary users of data.

10.4.3 Handling Requests for Information Disclosure

If a patient who participated in the study requests the disclosure of retained personal information that identifies the patient, in principle, the retained personal information must be disclosed. However, if the disclosure results in harm to the life, body, property, or other rights and interests of the patient or a third party, or if it significantly prevents the researchers from properly performing duties related to this study, all or part of the retained personal information may not be disclosed. If all or part of the requested personal information is not to be disclosed, the patient should be notified with explanation of the reasons.

10.4.4 Information Management System

Security control measures for the use of personal information will be established to minimize the risk of information leakage.

10.5 Necessity and System of Genetic Counseling

The study does not involve handling of genetic information, so genetic counseling will not be provided.

10.6 Protocol Compliance

Researchers participating in the study will comply with the study protocol as long as it does not jeopardize the safety or rights of the patients.

10.7 Approval by Certified Review Board and Submissions to Ministry of Health, Labour and Welfare

The conduct of this study using this protocol and patient information sheet must be approved by the CRB and permitted by the study site manager. In addition, an implementation plan* must be submitted to the Minister of Health, Labour and Welfare, and study information must be publicized in the jRCT before the start of the study. The Principal Investigator will be responsible for the application to the CRB, submission of the implementation plan to the Minister of Health, Labour and Welfare, and registration in the jRCT, and the Support Office will provide support for these application procedures.

* Form 1 of the Ministerial Order specified in Article 39 paragraph (1) of the Enforcement Regulations of the Clinical Trials Act

10.7.1 Initial Application Procedures

Procedures from Initial Application to Start of Study

Procedures should be followed according to the Clinical Trials Act and relevant regulations.

Any change in the study administrative structure (Section 15), addition or replacement of study sites, corresponds to a change in the protocol, and the change should be made following the change application procedures (Section 10.8).

10.7.2 Procedures Taken by the Study Site Investigator

After obtaining approval by the CRB, the investigator at the study site will obtain approval from the manager of his or her site. After obtaining approval from the manager, the investigator will promptly report it to the Support Office.

10.7.3 Permission for Conducting the Study at the Study Site

Procedures for obtaining permission for study conduct from the site manager will be done according to study site rules.

Changes to the study protocol, Information Sheet, and other documents approved by the CRB are not permitted, except for site contact details and selected items specified in advance. If there is a need for a change in the protocol or Information Sheet and the site manager requests a correction, the Principal Investigator and Support Office should be consulted.

10.8 Change Application Procedures

10.8.1 Procedures Taken by the Principal Investigator

If changes need to be made to the documents submitted during initial application to the CRB, the Principal Investigator should request the CRB to review the changes.

If the change involves a change to the implementation plan, the Principal Investigator should, after obtaining approval from the CRB, register the information in the jRCT and make a submission to the Minister of Health, Labour and Welfare before making the change, and immediately notify the CRB, site manager, and investigators.

Procedures for change application to the CRB will follow the Procedures from Initial Application to Start of Study in Section 10.7.1 “Initial Application Procedures”.

Minor changes, as defined by ministerial order, should be notified by the Principal Investigator to the CRB, as well as registered in the jRCT and submitted to the Minister of Health, Labour and Welfare within 10 days after the date of the change. If the study is not continued in some study sites, changes to the implementation plan will be submitted after the end of the patient observation period in the study site.

10.8.2 Procedures Taken by the Study Site Investigator

When the study site investigator receives information on CRB approval from the Principal Investigator, he or she will obtain approval from the manager of his or her site. After obtaining approval from the manager, the site investigator will promptly report it to the Support Office.

For changes that do not involve changes to the implementation plan, the site investigator will report these to the site manager.

If changes need to be made to the administrative structure of the study site, the site investigator should prepare conflicts of interest documents and list of subinvestigators as needed and contact the Principal Investigator and Support Office.

10.8.3 Study Progress and Review and Approval of Study Continuation (Periodic Report)

The Principal Investigator will report the status of items 1) through 5) below to the site manager, make a periodic report to the CRB, and undergo review on the appropriateness of continuing the study. He or she will report to the Minister of Health, Labour and Welfare within 1 month from the date the CRB provided its opinion and every year from the date of submission of the initial implementation plan within 2 months after the expiration of that period. If the Principal Investigator makes a report to the CRB, he or she should promptly provide this information to the site investigators, and the site investigators should report it to the manager of their sites.

1) Number of patients in the study (planned number of patients, number of patients providing consent, number of patients undergoing the study, number of patients who completed the study, number of patients who discontinued the study, and number of compensations made)
2) Occurrence of study-related illness and its prognosis
3) Occurrence of non-compliance with the ministerial ordinance related to the study or the protocol and subsequent actions
4) Assessment of safety and scientific validity of the study
5) Any changes to the conflict-of-interest management criteria or the conflict-of-interest management plan of the study

10.8.4 Reporting of the Progress of the Clinical Study

The Support Office will manage information on the study progress and the occurrence of illnesses and malfunctions as appropriate. Necessary information will be provided to the Principal Investigator for periodic reporting procedures.

10.9 Conflicts of Interest

10.9.1 Management of Study-Related Conflicts of Interest

Conflicts of interest related to the study will be managed according to the Guidance for Conflict of Interest Management in the Clinical Trials Act (Notification 1130-17 of the Director of the Research and Development Division of the Health Policy Bureau, MHLW dated November 30, 2018)* (hereafter referred to as the Guidance).

10.9.2 Study Funding Source/Funding Provider and Financial Interests

Chugai Pharmaceutical Co., Ltd. will provide funding for the study based on the contract concluded among the Department of Ophthalmology, Mie University Graduate School of Medicine, with which the Principal Investigator is affiliated; IQVIA Services Japan G.K., to which some duties are outsourced; and Chugai Pharmaceutical Co., Ltd. The study sites will conclude an outsourcing contract with IQVIA Services Japan G.K. and receive payment of study funds according to the

number of enrollment and other work related to the clinical study. These funds are not provided to promote enrollment, but to compensate for the study-related work done. The study will use a drug manufactured and marketed by Chugai Pharmaceutical Co., Ltd., but the company will not be directly involved in the monitoring, data management, statistical analyses, or audits of the study and will not affect the study results.

10.9.3 Information Disclosure

Information on the provision of study funds related to this study is subject to disclosure. Provision to organizations with which involved parties are affiliated is also subject to disclosure. This information will be published on the website of Chugai Pharmaceutical Co., Ltd. after the end of each business year.

Of note, some physicians participating in this study are receiving benefits from Chugai Pharmaceutical Co., Ltd., the funding provider; however, the conflict of interest existing between Chugai Pharmaceutical Co., Ltd. and these physicians has been reviewed by the Institutional Review Board of Mie University and has been determined that the conflict of interest is being properly managed and will not put patients participating in the study at a disadvantage.

10.10 Study Costs

10.10.1 Costs of Treatment

The study will be conducted within the scope of regular health insurance. All observations, tests, and drugs used during the study period will be covered by the health insurance of the patient, and the patient will pay for the costs within the scope of insurance care (the patient will also pay for the costs of travel for attending study visits). However, participation in the study may involve increased number of visits compared to usual care. Therefore, patients will receive 3000 yen (or actual cost up to 7000 yen with the consent of the study site if the travel expenses exceed 3000 yen) per visit as a payment for reducing patient burden when attending observation and testing visits for which there is no faricimab treatment.

10.10.2 Compensation for Injuries

If adverse events occur during the study, the investigator will promptly take necessary actions (e.g., perform tests, give treatment, or discontinue the patient from the study) to ensure the safety of the patients. In such a case, the investigator will provide the best medical care within the scope of health insurance. The Principal Investigator and the collaborator will purchase a clinical research compensation insurance and take other necessary actions before the start of the study to cover legal liabilities and compensation for injuries resulting from the study.

The investigator should establish a system for providing medical care and take other necessary actions for injuries that occur with the conduct of the study.

11. Quality Control and Quality Assurance

11.1 Study Records

The investigator must maintain adequate and accurate records to enable the conduct of the study to be fully documented, including the protocol, Informed Consent Forms, and documentation of site manager approval.

11.2 Monitoring

Monitoring will be performed by the Support Office personnel designated by the Principal Investigator to confirm that the study is being conducted safely and in accordance with the protocol and that data are being collected accurately. Monitoring involves central monitoring performed on the basis of data entries in the eCRFs collected at the data center. Monitoring should be conducted in accordance with the monitoring procedures determined separately.

11.2.1 Site Visit Monitoring

The frequency and detailed procedures for site visit monitoring will follow the monitoring procedures determined separately. Of note, the Collaborating Research Institute will not directly view the source documents which include personal patient information through site visits.

11.2.2 Audits

Site visit audits by the Support Office personnel designated by the Principal Investigator should be considered for the purpose of improving the scientific and ethical quality of the study. Procedures for site visit audits, including check items and frequency, will be determined separately. Auditors from the Support Office who engage in audit activities will report the results of the audit to the investigator. If necessary, this information will be provided to other investigators, as directed by the Principal Investigator.

11.3 Protocol Deviations and Violations

In this study, a protocol deviation or violation is defined as anything that was not performed in accordance with the terms of the protocol and is considered a situation of non-compliance of the clinical study with the Enforcement Regulations of the Clinical Trials Act or the protocol. If the investigator learns of any fact or information about something that has not been performed in accordance with the terms of the protocol, the investigator must report it to the manager of the site. Particularly, in the case of significant deviations or violations, the investigator must promptly seek the opinion of the CRB.

1) Violation

In principle, a “violation” is a deviation from the terms of the protocol that meets more than one of the following:

① Affects the study endpoints
② Is due to the investigator/study site
③ Is intentional or systematic
④ Is hazardous or significant degree of deviation

2) Deviation

Defined as a deviation that is not considered a “violation” or an “acceptable deviation.” If certain types of deviations occur frequently, these should be noted when the results are published in a paper.

Deviation: unfavorable and should be reduced

Deviation (unavoidable): does not need to be actively reduced

Deviation (clinically relevant): positively affirms the judgment of the investigator/site

3) Acceptable deviation

Defined as a protocol deviation within an acceptable range determined in advance or afterwards by the Principal Investigator.

12. Publication of Data

12.1 Public Database Registration

1) Publication of Implementation Plan

The implementation plan will be publicized by registering the study information in the database managed by the MHLW (jRCT) in advance. Similarly, changes to the implementation plan will be publicized by registering the changed information.

2) Publication of Primary Endpoint Report

Once the protocol-specified period for the collection of primary endpoint data is completed at all study sites, the investigator (Principle Investigator) should create a primary endpoint report (a report summarizing the results of primary endpoint data collection) and make changes to the implementation plan, in principle, within one year after the final day of the period. He or she should seek the opinion of the CRB about the primary endpoint report and the changed implementation plan in advance, submit them to the site manager without delay, and publicize them by registering the information in the jRCT within one month after the date the CRB provided its opinion.

3) Publication of Clinical Study Report Summary

Once the protocol-specified period for the collection of all endpoint data is completed at all study sites, the investigators and Principal Investigator should create the Clinical Study Report (a document summarizing the results of the study) and its summary (study results summary registered in the jRCT is also permitted), in principle, within one year after the final day of the period. The Clinical Study Report should include at least ① through ④ below:

① Demographics of patients in the study (e.g., age, sex)

② Information on the progress of the study according to the study design (e.g., change in number of patients)

③ Summary of the incidence of illnesses

④ Analyses of primary endpoint and secondary endpoint data and their results

They should seek in advance the opinion of the CRB about the Clinical Study Report and its summary prepared and submit them to the site manager without delay. The Clinical Study Report summary will be publicized by registering the information in the jRCT within one month after the date the CRB provided its opinion. Along with the Clinical Study Report summary, the protocol, Information Sheet, and SAP should be submitted to the Minister of Health, Labour and Welfare. If the primary endpoint report and the Clinical Study Report are to be created during the same period, the primary endpoint report is considered to have been created with the creation of the clinical study report.

12.2 Publication of Data

If the results of the study are published or presented at scientific meetings, arrangements will be made after discussion with the Principal Investigator, investigators, and the Collaborating Research Institute.

12.3 Layperson Summary

After publication of the main paper, a layperson summary (LPS), a document explaining the study results to study participants and their family, will be created based on the study results and data that have been published in the paper. The LPS will serve as a document used by researchers to explain the study results to study participants and/or their family, only when the researcher is asked by the study participants and/or their family to explain the study results.

13. Study Termination, Suspension, or Completion

13.1 Study Completion

The Study Administrator will notify the Principal Investigator of “study completion” once it decides to complete or terminate the study and approves the Clinical Study Report.

13.2 Study Termination or Suspension

If emergency safety measures are necessary, such as when marketing authorization for the study drug is revoked, or the occurrence of an unexpected serious adverse event results in the issuance of expedited safety information (Yellow Letter), the Study Administrator will seek the medical/ethical opinion of the Study Executive Committee and then make a decision to terminate or suspend the study. If it decides that the study must be terminated or suspended while the study is ongoing, the Study Administrator should promptly notify the study site managers in writing that the study has been terminated or suspended, providing a detailed reason. Each study site manager will notify the investigator and the CRB, providing a detailed written explanation. The investigator will notify the patients and ensure that they receive appropriate treatment and follow-up.

13.3 Clinical Study Report

If the Support Office receives a notification of the completion or termination of the study, it will prepare the Clinical Study Report by summarizing the results of the study and submit the Clinical Study Report (Draft) to the Study Administrator without delay. The Study Administrator will interpret the results and finalize the document.

13.4 Procedures at Study Completion

After confirming that the study has been completed, the Principal Investigator will submit a summary of the results and a report that the study has been completed to the study site investigators. The summary of the study results (study completion report) will be distributed to the study site investigators via the Support Office at a time decided by the Study Administrator and the Principal Investigator in consideration of the timing of publication of the main results. After receiving the study completion report, the study site investigators will promptly follow the procedures to complete the study as per the rules of their study sites. Study sites that did not enroll any patients may consider the final day of enrollment to be the day of study completion at that site.

14. Ownership of Study Results

The results from this study are the property of National University Corporation Mie University and Chugai Pharmaceutical Co., Ltd., which is the Collaborating Research Institute. The ownership and management of intellectual property rights of any inventions and resulting patents,

improvements, and/or know-how originating from the use of data from this study will be determined in discussion between National University Corporation Mie University and Chugai Pharmaceutical Co., Ltd., which is the Collaborating Research Institute. All intellectual property rights related to the drugs manufactured and marketed by Chugai Pharmaceutical Co., Ltd. belong to Chugai Pharmaceutical Co., Ltd.

15. Study Administrative Structure

15.1 Study Administrator

Department of Ophthalmology, Mie University Graduate School of Medicine

2-174 Edobashi, Tsu, Mie 514-8507, Japan

Tel: +81-59-232-1111 (main)

15.1.1 Principal Investigator

Department of Ophthalmology, Mie University Graduate School of Medicine
Mineo Kondo, Professor, Department of Ophthalmology
2-174 Edobashi, Tsu, Mie 514-8507, Japan
Tel: +81-59-232-1111 (main)

Roles and Responsibilities of the Principal Investigator

The Principal Investigator takes the role as the coordinator of the entire study organization including all study sites and is responsible for conducting the study in compliance with the Clinical Trials Act.

15.2 Collaborating Research Institute

Chugai Pharmaceutical Co., Ltd.
2-1-1 Nihonbashi-Muromachi, Chuo-ku, Tokyo 103-8324, Japan
Tel: +81-3-3281-6611

15.2.1 Head of the Collaborating Research Institute

Osamu Okuda, Representative Director, President & CEO, Chugai Pharmaceutical Co., Ltd.

Roles and Responsibilities of the Head of the Collaborating Research Institute

The Head of the Collaborating Research Institute will oversee that the study is being conducted properly at the Collaborating Research Institute in accordance with the Clinical Trials Act and the protocol. He or she will take appropriate measures in case of violations of the company regulations of the collaborating institute or the protocol.

15.2.2 Supervisory Manager of the Collaborating Research Institute

Kazuhiko Nishi, Head of Medical Affairs Div., Chugai Pharmaceutical Co., Ltd.
2-1-1 Nihonbashi-Muromachi, Chuo-ku, Tokyo 103-8324, Japan
Tel: +81-3-3281-6611

Roles and Responsibilities of the Supervisory Manager of the Collaborating Research Institute

1) Conclusion of contracts related to funding for the study

2) Overall supervision of the study
   ① He or she is responsible for undertaking necessary supervision to ensure that work is carried out properly in the Collaborating Research Institute in the study.
   ② He or she will confirm as necessary that work in the Collaborating Research Institute is performed properly and in accordance with the above-mentioned guidelines and the study protocol, as well as take necessary measures to ensure proper conduct of the study.
   ③ In the Collaborating Research Institute, he or she must give thorough instructions to all personnel involved in the conduct of the authorized study to conduct the study with respect for the life, health, and rights of study participants, their family, and relevant others, and make them aware that violations of the company regulations of the Collaborating Research Institute or the study protocol may result in disciplinary action or other penalties.
   ④ He or she must not disclose the information that he or she comes to know in the course of his or her work without justifiable grounds. The same applies when he or she is no longer engaging in the work.
   ⑤ In the Collaborating Research Institute, he or she must execute a written contract that includes provisions with which contractors must comply if part of the work related to the authorized study is outsourced; and must undertake necessary and appropriate supervision of the contractors.
3) Development of systems and regulations for performing work for the study
   ① In the Collaborating Research Institute, he or she must ensure that patients receive compensation or other necessary measures for any injuries related to the authorized study.
   ② He or she must ensure that the study results and other study-related information are publicized appropriately, after taking necessary measures to protect the rights of study participants, their family, and relevant others or the rights and interests of researchers and relevant others.
   ③ In the Collaborating Research Institute, he or she must inspect and evaluate as necessary whether the authorized study complies with the company regulations of the Collaborating Research Institute, and must take appropriate action based on the results.
   ④ In the Collaborating Research Institute, he or she must take measures to ensure that the researchers of the authorized study receive education and training on research ethics as well as on knowledge and techniques necessary for the conduct of the study. He or she must also receive such education and training.

15.2.3 Manager of the Collaborating Research Institute

Manager of the Collaborating Research Institute

Jun Tsujimura, Medical Manager, Specialty Medical Science Dept., Chugai Pharmaceutical Co., Ltd.

2-1-1 Nihonbashi-Muromachi, Chuo-ku, Tokyo 103-8324, Japan

Tel: +81-3-3281-6611

Roles and Responsibilities of the Manager of the Collaborating Research Institute

- He or she will cooperate with the Principle Investigator to develop the implementation plan.
- The Manager of the Collaborating Research Institute is responsible for properly carrying out the duties of the Collaborating Research Institute in the study in accordance with the company regulations of the research institute and the study protocol.
- He or she will notify and explain the publicized study results to the sites participating in the study.

15.3 Funding Provider

Kazuhiko Nishi, Head of Medical Affairs Div., Chugai Pharmaceutical Co., Ltd.
2-1-1 Nihonbashi-Muromachi, Chuo-ku, Tokyo 103-8324, Japan
Tel: +81-3-3281-6611

Roles and Responsibilities of the Funding Provider

The Funding Provider is responsible for providing study-related funds and appropriate disclosure of information, and bears a responsibility as a collaborator of the study.

15.4 Clinical Study Office

Department of Ophthalmology, Mie University Graduate School of Medicine
Mineo Kondo, Professor, Department of Ophthalmology
2-174 Edobashi, Tsu, Mie 514-8507, Japan
Tel: +81-59-232-1111 (main)

Roles of the Clinical Study Office

The Clinical Study Office will handle matters related to the study protocol that involve medical decisions.

15.5 Study Sites

Each selected site will determine whether it is possible to conduct the study. See Attachment 1 for the study sites that have been determined to participate in this study (other sites will also participate later).

15.6 Person Responsible for Statistical Analysis

Statistical Analysis
IQVIA Services Japan G.K.
Keisuke Takada
4-10-18 Takanawa, Minato-ku, Tokyo 108-0074, Japan
Tel: +81-3-6859-9500

Roles of the Person Responsible for Statistical Analysis

- Management and oversight of statistical analysis duties
- Validation of analysis results

15.7 Support Office

IQVIA Services Japan G.K.
Keikyu First Building, 4-10-18 Takanawa, Minato-ku, Tokyo 108-0074, Japan
Tel: +81-3-6859-9500

Roles of the Support Office

The Support Office serves as a study contact to provide support for the study management.

15.8 Organization Responsible for Monitoring

Clinical Operations
IQVIA Services Japan G.K.
Tomohito Ide
Tatsuya Watanabe
4-10-18 Takanawa, Minato-ku, Tokyo 108-0074, Japan
Tel: +81-3-6859-9500

Roles and Responsibilities of the Organization Responsible for Monitoring

The Organization Responsible for Monitoring will carry out the activities of monitoring.

15.9 Organization Responsible for Data Management

Data Management
IQVIA Services Japan G.K.
Ayaka Mitsumune
4-10-18 Takanawa, Minato-ku, Tokyo 108-0074, Japan
Tel: +81-3-6859-9500

Roles and Responsibilities of the Organization Responsible for Data Management

The Organization Responsible for Data Management will carry out the activities of enrollment and data management, and is responsible for assuring data quality.

15.10 Auditing

Quality Assurance
IQVIA Services Japan G.K.
Katsura Nakamura
4-10-18 Takanawa, Minato-ku, Tokyo 108-0074, Japan
Tel: +81-3-6859-9500

Roles and Responsibilities of the Organization Responsible for Auditing

The Organization Responsible for Auditing will carry out the activities of audit work and is responsible for the contents of the audit.

15.11 Person Responsible for Coordination and Management

IQVIA Services Japan G.K.
Manager: Misa Arata

Keikyu First Building, 4-10-18 Takanawa, Minato-ku, Tokyo 108-0074, Japan
Tel: +81-3-6859-9500

Roles and Responsibilities of the Person Responsible for Coordination and Management

- Management of the progress and budget of the study
- Undertaking of procedures necessary for the study, proper management of documents, and ensuring of reliability of collected data
- Communication/coordination and exchange of information with the relevant parties involved in the study

15.12 Image Analysis Institution

Micron, Inc.
3-13-16 Mita, Minato-ku, Tokyo 108-0073, Japan

Roles of the Image Analysis Institution
The Image Analysis Institution will carry out image analysis activities.

15.13 Study Executive Committee

Motohiro Kamei, Professor, Department of Ophthalmology, Aichi Medical University
Masahiko Shimura, Professor, Department of Ophthalmology, Tokyo Medical University Hachioji Medical Center
Akitaka Tsujikawa, Professor, Department of Ophthalmology and Visual Sciences, Kyoto University Graduate School of Medicine

Roles and Responsibilities of the Study Executive Committee

- Make medical decisions on the conduct of the study
- Provide advice and proposals on the preparation and modification of the Protocol
- Provide advice and proposals on the preparation and revision of the sample Informed Consent Form
- Provide advice and proposals on the design and creation of the eCRFs
- Select candidate study sites
- Review progress of the study
- Prepare and issue letters to encourage eCRF entries
- Review the publication plan
- Discuss the Statistical Analysis Plan
- Agree to the Clinical Study Report
- Share information with study site investigators
- Engage in other tasks determined in consultation with the Study Administrator

15.14 Medical Expert Advisor (Image Analysis)

Yuki Muraoka, Program Specific Lecturer, Department of Ophthalmology and Visual Sciences, Kyoto University Hospital

Roles and Responsibilities of the Medical Expert Advisor

The Medical Expert Advisor will provide advice on image analysis and the interpretation of the results.

16. References

1. Song P, Xu Y, Zha M,et al. Global epidemiology of retinal vein occlusion: a systematic review and meta-analysis of prevalence, incidence, and risk factors. J Glob Health. 2019 Jun;9(1):010427. doi: 10.7189/jogh.09.010427. PMID: 31131101; PMCID: PMC6513508.
2. Tadayoni R, Paris LP, Danzig CJ, et al. and COMINO Investigators. Efficacy and Safety of Faricimab for Macular Edema due to Retinal Vein Occlusion: 24-Week Results from the BALATON and COMINO Trials. Ophthalmology. 2024 Aug;131(8):950-960. doi: 10.1016/j.ophtha.2024.01.029. Epub 2024 Jan 26. PMID: 38280653.
3. Hayreh SS, Zimmerman MB, Podhajsky P. Incidence of various types of retinal vein occlusion and their recurrence and demographic characteristics. Am J Ophthalmol. 1994 Apr 15;117(4):429-41. doi: 10.1016/s0002-9394(14)70001-7. PMID: 8154523.
4. Boyd SR, Zachary I, Chakravarthy U, et al. Correlation of increased vascular endothelial growth factor with neovascularization and permeability in ischemic central vein occlusion. Arch Ophthalmol. 2002 Dec;120(12):1644-50. doi: 10.1001/archopht.120.12.1644. PMID: 12470137.
5. Laouri M, Chen E, Looman M,et al. The burden of disease of retinal vein occlusion: review of the literature. Eye (Lond). 2011 Aug;25(8):981-8. doi: 10.1038/eye.2011.92. Epub 2011 May 6. PMID: 21546916; PMCID: PMC3178209.
6. Yasuda M, Kiyohara Y, Arakawa S,et al. Prevalence and systemic risk factors for retinal vein occlusion in a general Japanese population: the Hisayama study. Invest Ophthalmol Vis Sci. 2010 Jun;51(6):3205-9. doi: 10.1167/iovs.09-4453. Epub 2010 Jan 13. PMID: 20071683.
7. A randomized clinical trial of early panretinal photocoagulation for ischemic central vein occlusion. The Central Vein Occlusion Study Group N report. Ophthalmology. 1995 Oct;102(10):1434-44. PMID: 9097789.
8. Ip MS, Scott IU, VanVeldhuisen PC,et al.; SCORE Study Research Group. A randomized trial comparing the efficacy and safety of intravitreal triamcinolone with observation to treat vision loss associated with macular edema secondary to central retinal vein occlusion: the Standard Care vs Corticosteroid for Retinal Vein Occlusion (SCORE) study report 5. Arch Ophthalmol. 2009 Sep;127(9):1101-14. doi: 10.1001/archophthalmol.2009.234. Erratum in: Arch Ophthalmol. 2009 Dec;127(12):1648. PMID: 19752419; PMCID: PMC2872173.
9. Campochiaro PA, Hafiz G, Mir TA, Scott AW, Solomon S, Zimmer-Galler I, Sodhi A, Duh E, Ying H, Wenick A, Shah SM, Do DV, Nguyen QD, Kherani S, Sophie R. Scatter Photocoagulation Does Not Reduce Macular Edema or Treatment Burden in Patients with Retinal Vein Occlusion: The RELATE Trial. Ophthalmology. 2015 Jul;122(7):1426-37. doi: 10.1016/j.ophtha.2015.04.006. Epub 2015 May 9. PMID: 25972260; PMCID: PMC10020833.
10. Heier JS, Singh RP, Wykoff CCet al. THE ANGIOPOIETIN/TIE PATHWAY IN RETINAL VASCULAR DISEASES: A Review. Retina. 2021 Jan 1;41(1):1-19. doi: 10.1097/IAE.0000000000003003. PMID: 33136975.
11. Joussen AM, Ricci F, Paris LP,et al. Angiopoietin/Tie2 signalling and its role in retinal and choroidal vascular diseases: a review of preclinical data. Eye (Lond). 2021 May;35(5):1305-1316. doi: 10.1038/s41433-020-01377-x. Epub 2021 Feb 9. PMID: 33564135; PMCID: PMC8182896.
12. Saharinen P, Eklund L, Alitalo K. Therapeutic targeting of the angiopoietin-TIE pathway. Nat Rev Drug Discov. 2017 Sep;16(9):635-661. doi: 10.1038/nrd.2016.278. Epub 2017 May 19.

PMID: 28529319.

13. Benest AV, Kruse K, Savant S, et al. Angiopoietin-2 is critical for cytokine-induced vascular leakage. PLoS One. 2013 Aug 5;8(8):e70459. doi: 10.1371/journal.pone.0070459. PMID: 23940579; PMCID: PMC3734283.
14. Regula JT, Lundh von Leithner P, Foxton R, et al. Targeting key angiogenic pathways with a bispecific crossMAb optimized for neovascular eye diseases. EMBO Mol Med. 2016 Nov 2;8(11):1265-1288. doi: 10.15252/emmm.201505889. Erratum in: EMBO Mol Med. 2019 May;11(5):e10666. doi: 10.15252/emmm.201910666. PMID: 27742718; PMCID: PMC5090659.
15. Foxton RH, Uhles S, Grüner S, et al. Efficacy of simultaneous VEGF-A/ANG-2 neutralization in suppressing spontaneous choroidal neovascularization. EMBO Mol Med. 2019 May;11(5):e10204. doi: 10.15252/emmm.201810204. PMID: 31040126; PMCID: PMC6505683.
16. Canonica J, Foxton R, Garrido MG, et al. Delineating effects of angiopoietin-2 inhibition on vascular permeability and inflammation in models of retinal neovascularization and ischemia/reperfusion. Front Cell Neurosci. 2023 Jun 12;17:1192464. doi: 10.3389/fncel.2023.1192464. PMID: 37377777; PMCID: PMC10291265.
17. Heier JS, Khanani AM, Quezada Ruiz C, et al.; TENAYA and LUCERNE Investigators. Efficacy, durability, and safety of intravitreal faricimab up to every 16 weeks for neovascular age-related macular degeneration (TENAYA and LUCERNE): two randomised, double-masked, phase 3, non-inferiority trials. Lancet. 2022 Feb 19;399(10326):729-740. doi: 10.1016/S0140-6736(22)00010-1. Epub 2022 Jan 24. PMID: 35085502.
18. Wykoff CC, Abreu F, Adamis AP, et al.; YOSEMITE and RHINE Investigators. Efficacy, durability, and safety of intravitreal faricimab with extended dosing up to every 16 weeks in patients with diabetic macular oedema (YOSEMITE and RHINE): two randomised, double-masked, phase 3 trials. Lancet. 2022 Feb 19;399(10326):741-755. doi: 10.1016/S0140-6736(22)00018-6. Epub 2022 Jan 24. PMID: 35085503.
19. Package Insert for VABYSMO. Revised in March 2024 (3rd edition). Chugai Pharmaceutical Co., Ltd.
https://www.pmda.go.jp/PmdaSearch/iyakuDetail/ResultDataSetPDF/450045_1319408A1020_1_03
Last access date: September 20, 2024
20. Campochiaro PA, Heier JS, Feiner L, et al.; BRAVO Investigators. Ranibizumab for macular edema following branch retinal vein occlusion: six-month primary end point results of a phase III study. Ophthalmology. 2010 Jun;117(6):1102-1112.e1. doi: 10.1016/j.ophtha.2010.02.021. Epub 2010 Apr 15. PMID: 20398941.
21. Tadayoni R, Waldstein SM, Boscia F, et al.; BRIGHTER Study Group. Sustained Benefits of Ranibizumab with or without Laser in Branch Retinal Vein Occlusion: 24-Month Results of the BRIGHTER Study. Ophthalmology. 2017 Dec;124(12):1778-1787. doi: 10.1016/j.ophtha.2017.06.027. Epub 2017 Aug 12. Erratum in: Ophthalmology. 2018 Mar;125(3):463. doi: 10.1016/j.ophtha.2017.12.012. PMID: 28807635.
22. Campochiaro PA, Clark WL, Boyer DS, et al. Intravitreal aflibercept for macular edema following branch retinal vein occlusion: the 24-week results of the VIBRANT study. Ophthalmology. 2015 Mar;122(3):538-44. doi: 10.1016/j.ophtha.2014.08.031. Epub 2014 Oct

12. PMID: 25315663.

23. Brown DM, Campochiaro PA, Singh RP, et al.; CRUISE Investigators. Ranibizumab for macular edema following central retinal vein occlusion: six-month primary end point results of a phase III study. Ophthalmology. 2010 Jun;117(6):1124-1133.e1. doi: 10.1016/j.ophtha.2010.02.022. Epub 2010 Apr 9. PMID: 20381871.
24. Ogura Y, Roider J, Korobelnik JF, et al.; GALILEO Study Group. Intravitreal aflibercept for macular edema secondary to central retinal vein occlusion: 18-month results of the phase 3 GALILEO study. Am J Ophthalmol. 2014 Nov;158(5):1032-8. doi: 10.1016/j.ajo.2014.07.027. Epub 2014 Jul 25. PMID: 25068637.
25. Heier JS, Clark WL, Boyer DS, et al.. Intravitreal aflibercept injection for macular edema due to central retinal vein occlusion: two-year results from the COPERNICUS study. Ophthalmology. 2014 Jul;121(7):1414-1420.e1. doi: 10.1016/j.ophtha.2014.01.027. Epub 2014 Mar 27. Erratum in: Ophthalmology. 2014 Nov;121(11):2293. PMID: 24679444.
26. Heier JS, Campochiaro PA, Yau L, Li Z, et al. Ranibizumab for macular edema due to retinal vein occlusions: long-term follow-up in the HORIZON trial. Ophthalmology. 2012 Apr;119(4):802-9. doi: 10.1016/j.ophtha.2011.12.005. Epub 2012 Feb 1. PMID: 22301066.
27. Shimura M, Fukumatsu M, Tsujimura J,et al.; Participating Investigators. Real-World Data on Intravitreal Aflibercept for Macular Edema Secondary to Central Retinal Vein Occlusion: 24-Month Outcomes. Clin Ophthalmol. 2022 Mar 1;16:579-592. doi: 10.2147/OPTH.S344194. PMID: 35256840; PMCID: PMC8898177.
28. Shimura M, Kitano S, Muramatsu D, et al.; Japan Clinical Retina Study (J-CREST) group. Real-world management of treatment-naïve diabetic macular oedema in Japan: two-year visual outcomes with and without anti-VEGF therapy in the STREAT-DME study. Br J Ophthalmol. 2020 Sep;104(9):1209-1215. doi: 10.1136/bjophthalmol-2019-315199. Epub 2019 Nov 29. PMID: 31784500; PMCID: PMC7577088.
29. Hattenbach LO, Abreu F, Arrisi P, et al. BALATON and COMINO: Phase III Randomized Clinical Trials of Faricimab for Retinal Vein Occlusion: Study Design and Rationale. Ophthalmol Sci. 2023 Mar 27;3(3):100302. doi: 10.1016/j.xops.2023.100302. PMID: 37810589; PMCID: PMC10556281.
30. Sheryl Stevenson. Angiogenesis 2024: Highlighting 72-week results from the BALATON and COMINO Phase 3 studies of faricimab in RVO. 2024 Feb. https://www.modernretina.com/view/angiogenesis-2024-highlighting-72-week-results-from-the-balaton-and-comino-phase-3-studies-of-faricimab-in-rvo
31. Shimura M, Fukumatsu M, Tsujimura J,, et al.; Participating Investigators. Real-world data on intravitreal aflibercept for macular edema secondary to central retinal vein occlusion: 24-month outcomes. Clin Ophthalmol. 2022;16:579–592.
32. Ozdemir S, Finkelstein E, Lee JJ, et al. Understanding patient preferences in anti-VEGF treatment options for age-related macular degeneration. PLoS One. 2022 Aug 11;17(8):e0272301. doi: 10.1371/journal.pone.0272301. PMID: 35951503; PMCID: PMC9371344.
33. Appropriate Use Guide for VABYSMO solution for Intravitreal Injection 120mg/mL. March 2024. Chugai Pharmaceutical Co., Ltd. https://www.pmda.go.jp/RMP/www/450045/2e72f5fb-b81f-41ce-b2c7-d662f67b5d94/450045_1319408A1020_01_003RMPm.pdf

Last access date: September 20, 2024

34. Gregori NZ, Feuer W, Rosenfeld PJ. Novel method for analyzing snellen visual acuity measurements. Retina. 2010 Jul-Aug;30(7):1046-50. doi: 10.1097/IAE.0b013e3181d87e04. PMID: 20559157.

Appendix 1 Schedule of Activities

	Screening	W0	W4	W8	W12	W16	W20	W24	W28	W32	W36	W40	W44	W48	W52	W56	W60	W64	W68	W72	Unscheduled
Visit Window (days)	D-14～D1	D1	(-7, +14)	(-7, +14)	(-7, +14)	(-7, +14)	(-7, +14)	(-7, +14)	(-7, +14)	(-7, +14)	(-7, +14)	(-7, +14)	(-7, +14)	(-7, +14)	(-7, +14)	(-7, +14)	(-7, +14)	(-7, +14)	(-7, +14)	(-7, +14)	
Scheduled visit	○	○	○	○	○	○	○	○			○[a]				○					○	
Informed consent	○[b]																				
Review of inclusion and exclusion criteria	○																				
Medical and surgical history	○																				
Patient demographics	○																				
Blood pressure (systolic and diastolic)	○	○[c]													○					○	
Refraction test [d]	○	○[c]																			
Axial length test	●	●[c]																			
Study treatment [e]		↔	↔	↔	↔	↔	↔	↔	↔	↔	↔	↔	↔	↔	↔	↔	↔	↔	↔	↔	●
Concomitant medication/therapy		○	○	○	○	○	○	○	○[f]	○[f]	○[f]	○[f]	○[f]	○[f]	○	○[f]	○[f]	○[f]	○[f]	○	○
Adverse events		↔	↔	↔	↔	↔	↔	↔	↔	↔	↔	↔	↔	↔	↔	↔	↔	↔	↔	↔	○
Visual acuity test [d, g]	○	○[c]	○	○	○	○	○	○	○[f]	○[f]	○[f]	○[f]	○[f]	○[f]	○	○[f]	○[f]	○[f]	○[f]	○	○
Intraocular pressure test [d, h]	○	○[c]	○	○	○	○	○	○	○[f]	○[f]	○[f]	○[f]	○[f]	○[f]	○	○[f]	○[f]	○[f]	○[f]	○	○
SD-OCT or SS-OCT [d, i]	○	○[c]	○	○	○	○	○	○	○[f]	○[f]	○[f]	○[f]	○[f]	○[f]	○	○[f]	○[f]	○[f]	○[f]	○	○
OCT-A [d, i]	○	○[c]	○	○	○	○	○	○	○[f]	○[f]	○[f]	○[f]	○[f]	○[f]	○	○[f]	○[f]	○[f]	○[f]	○	○
FA [d, j]	○	○[c]													○						
CFP [d, j]	○	○[c]													○						

CFP = color fundus photography; FA = fluorescein angiography; OCT-A = optical coherence tomography-angiography; SD-OCT = spectral-domain optical coherence tomography; SS-OCT = swept source optical coherence tomography; W = Week; D = Day; ○ = mandatory; ● = optional.

a. This will be a scheduled visit for only patients in the observation phase.

[b] Results of tests and assessments performed as part of routine care within 14 days before Day 1, even prior to obtaining informed consent, may be used, and such tests and assessments do not need to be repeated for screening.

[c] It will be unnecessary on Day 1 if performed at screening.

[d] Ocular tests are mandatory for the study eye.

[e] The study drug on Day 1 should be given within 28 days after obtaining informed consent, and the next dose should be at least 21 days apart. Finger counting will be assessed within 15 minutes after study drug administration to confirmed that there is no problem with visual function.

[f] It will be measured during the visit.

[g] Visual acuity will be tested using a Landolt ring chart at a distance of 5 m.

[h] IOP of the study eye will be measured before dilation for ocular tests, and if IOP before dilation is ≥30 mmHg, administration of dilating drops and study drug should be stopped. If possible, IOP of the study eye will also be measured 30 minutes after study drug administration.

[i] It will be performed, in principle, after pupillary dilation.

[j] FA and CFP should follow written procedures.

Appendix 2 eCRF Entries

(1) Enrollment Form

Investigation item	Collected information
Patient demographics	Patient identification number<br>Sex<br>Age at initial informed consent<br>Year and month of birth<br>Race
Planned date of initiation of treatment	Planned date of initiation of treatment
Study eye	Study eye (right or left eye)
Informed consent	Written informed consent (yes or no)<br>(If yes) date of written informed consent
Inclusion and exclusion criteria	Inclusion criteria (meeting or not meeting)<br>Exclusion criteria (meeting or not meeting)

(2) Baseline (information at enrollment)

Investigation item	Collected information
Past medical history and concomitant disease	Past medical history/concomitant disease (yes or no) (if yes, whether it is past medical history or concomitant disease, name of disease)
History of surgery	History of surgery (yes or no) (if yes, name of disease, name of procedure, date of surgery)
History of surgery (eye disease)	History of surgery (yes or no) (if yes, name of disease, target eye, name of procedure, date of surgery)
Prior and concomitant medication	Name of drug, route of administration, daily dose, start/end date of treatment or ongoing, reason for administration
Prior and concomitant medication (eye disease)	Name of drug, target eye, route of administration, daily dose, start/end date of treatment or ongoing, reason for administration
Blood pressure	Systolic and diastolic blood pressure<br>Use of antihypertensives (yes or no)
IOP	IOP (study eye)
Refraction test	Spherical power, cylindrical power, and cylinder axis (study eye)<br>Abnormality (yes or no) (study eye)
Axial length	Axial length (study eye)
Visual acuity test	Decimal visual acuity (study eye)<br>(If decimal visual acuity is <0.02) finger-counting, hand motion, or light perception
SD-OCT or SS-OCT	CST (study eye)<br>IRF, SRF, and epimacular membranes (yes or no) (study eye)

FA	Date of imaging
CFP	Date of imaging
OCT-A	Vascular density of the superficial, deep, and hole capillary plexuses<br>Date of imaging

(3) Case report form

Investigation item	Collected information
Visit	Visit date
Blood pressure<br>(Weeks 52 and 72 only)	Systolic and diastolic blood pressures<br>Use of antihypertensives (yes or no)
IOP	Pre-treatment IOP (study eye)
Visual acuity test	Decimal visual acuity (study eye)<br>(If decimal visual acuity is <0.02) finger-counting, hand motion, or light perception<br>Finger-counting (within 15 minutes after treatment)
SD-OCT or SS-OCT	CST (study eye)<br>IRF, SRF, and epimacular membranes (yes or no) (study eye)
FA<br>(Week 52 only)	Date of imaging
CFP<br>(Week 52 only)	Date of imaging
OCT-A	Vascular density of the superficial, deep, and hole capillary plexuses<br>Date of imaging
IVT administration of study drug	Administration (yes or no) (if yes, date of administration, dose)<br>(If dose is changed, reason for the change)
Concomitant medication	Concomitant medication (yes or no) (if yes, name of drug, route of administration, daily dose, start date, end date, or ongoing, reason for administration)
Concomitant therapy	Concomitant therapy (yes or no) (if yes, name of therapy, start date, end date, or ongoing, reason for treatment)
Adverse event	Adverse event (if yes, name of adverse event, date of onset, seriousness [if serious, the reason], severity, outcome and date of outcome assessment, relationship to the study drug, action taken for the adverse event
Treatment status	Date confirmed<br>Ongoing, completed, or discontinued (if discontinued, date of discontinuation, reason for discontinuation)
