# Supplementary material for: A multicenter, single-arm study using a modified faricimab treat-and-extend regimen in patients with macular edema due to central retinal vein occlusion: RVOSTAR study design protocol
Source: PLoS One. 2025 Oct 30;20(10):e0335015. doi: 10.1371/journal.pone.0335015 (PMC12574838; doi:10.1371/journal.pone.0335015)
Supplement: S2 File — RVOSTAR study protocol (Japanese). (PDF) [file pone.0335015.s004.pdf]

研究実施計画書

標題：	網膜中心静脈閉塞症（CRVO）に伴う黄斑浮腫患者に対するファリシマブを用いた modified treat-and-extend（mTAE）レジメンの検討<br>略称：RVO treatment Strategy with modified Treat And extend Regimen of faricimab（RVOSTAR）study
研究実施計画書番号：	CMA-0210
版番号：	第1.0版
作成日	2025年2月28日
研究薬：	ファリシマブ（遺伝子組換え）
研究代表医師	三重大学大学院医学系研究科　臨床医学系講座　眼科学<br>教授　近藤　峰生
共同研究機関	中外製薬株式会社
研究事務局	三重大学大学院医学系研究科　臨床医学系講座　眼科学<br>教授　近藤　峰生
支援事務局	IQVIA サービシーズ ジャパン合同会社
CRB 承認日	2025年3月24日

目次

研究実施計画書 1
研究実施計画書要約 7
略語及び用語の定義 15
1.　背景 17
1.1　対象疾患の背景 17
1.2　ファリシマブの背景 17
1.3　研究計画設定の根拠 17
2.　目的及び評価項目 18
2.1　目的 18
2.2　評価項目 18
2.2.1　主要評価項目 18
2.2.2　副次的評価項目 18
2.2.3　探索的評価項目 19
2.2.4　安全性評価項目 19
3.　本研究の概要 20
3.1　本研究の種類 20
3.2　研究のデザイン 20
3.3　研究の終了と研究期間 20
3.4　研究デザインの設定根拠 21
3.5　目標症例数の設定根拠 21
3.6　本研究の意義 21
3.7　研究参加に伴い予想される利益と不利益 22
4.　対象と方法 24
4.1　対象 24
4.1.1　選択基準 24
4.1.2　除外基準 24
4.2　登録 26
4.2.1　登録の手順 26
4.2.2　登録結果の発行と通知 26
4.2.3　複数研究登録について 27
4.2.4　登録に際しての注意事項 27
4.2.5　登録終了の手続き 27
4.3　観察・検査項目スケジュール 27
4.3.1　同意取得及びスクリーニング記録 27
4.3.2　病歴・手術歴及び患者背景情報 28
4.3.3　血圧 28

4.3.4　眼科検査 28
4.3.4.1　眼圧測定 28
4.3.4.2　屈折検査 28
4.3.4.3　眼軸長検査 28
4.3.4.4　視力検査 28
4.3.4.5　眼底検査 28
4.3.5　光干渉断層撮影（OCT） 29
4.3.6　規定外来院 29
4.3.7　患者の研究の中止 29
4.3.8　実施医療機関における研究参加中止 29
5.　治療計画と治療変更基準 30
5.1　研究薬 30
5.2　プロトコール治療 30
5.3　プロトコール治療中断／中止基準 31
5.4　併用療法・支持療法 32
5.4.1　規定とする併用療法・支持療法 32
5.4.2　推奨される／推奨されない併用療法・支持療法 33
5.4.3　許容される併用療法・支持療法 33
5.4.4　併用禁止薬剤及び療法 33
5.5　後治療 33
6.　安全性評価 33
6.1　個々の薬剤で予期される薬物有害事象 33
6.2　有害事象の定義 33
6.2.1　有害事象の定義 33
6.2.2　疾病等の定義 34
6.2.3　有害事象の程度 34
6.2.4　有害事象の重症度判定 35
6.2.5　因果性分類判定基準 35
7.　有害事象又は健康被害の恐れのある機器の不具合の報告 35
7.1　有害事象又は健康被害の恐れのある機器の不具合の報告期間 35
7.2　すべての有害事象又は健康被害の恐れのある機器の不具合発生時の対応 36
7.3　疾病等、感染症への対応 36
7.4　有害事象発現後の患者フォローアップ 37
7.5　女性患者の妊娠 37
7.6　過量投与／投薬過誤／薬物乱用／薬物誤用に関する報告 37
8.　統計学的考察及び解析計画 37
8.1　予定登録数・予定総研究期間 37
8.2　解析対象集団 38

8.3　主要評価項目の解析 38
8.4　副次的評価項目の解析 39
8.5　探索的評価項目の解析 39
8.6　安全性評価項目の解析 39
8.7　中間解析 39
8.8　研究の終了 39
9.　データの収集と管理 39
9.1　データの品質保証 39
9.2　電子症例報告書（eCRF） 39
9.2.1　eCRF の項目 40
9.3　原データの特定 40
9.3.1　eCRF のみに入力されているデータ 40
9.3.2　原資料 40
9.3.3　コンピューター化システムの使用 40
9.4　記録・情報・試料の取扱い及び保存 40
9.4.1　実施医療機関 40
9.4.2　共同研究機関 40
9.4.3　支援事務局 40
9.4.4　画像解析機関 41
9.5　情報提供の記録の保存及び廃棄 41
10.　倫理的事項 41
10.1　患者の保護 41
10.2　インフォームドコンセント 41
10.2.1　説明と同意 41
10.2.2　同意撤回 42
10.3　患者から相談等への対応 42
10.4　個人情報の保護と患者の識別 43
10.4.1　個人情報の利用目的と利用する項目及び利用方法 43
10.4.2　データの 2 次利用について 43
10.4.3　情報開示等に対する対応 44
10.4.4　情報管理体制 44
10.5　遺伝子カウンセリングの必要性及びその体制 44
10.6　研究実施計画書の遵守 44
10.7　CRB の承認及び厚生労働省への届け出 44
10.7.1　新規申請手続き 44
10.7.2　実施医療機関の研究責任医師が行う手続き 44
10.7.3　実施医療機関での研究実施許可について 44
10.8　変更申請時の手続き 45

10.8.1　研究代表医師が行う手続き........45
10.8.2　実施医療機関の研究責任医師が行う手続き........45
10.8.3　研究の進捗状況や研究継続に関する審査・承認（定期報告）........45
10.8.4　臨床研究の進捗状況等報告........45
10.9　利益相反........46
10.9.1　本研究に係る利益相反管理について........46
10.9.2　臨床研究の資金源／資金提供及び財政上の関係........46
10.9.3　情報公開........46
10.10　研究の費用........46
10.10.1　治療に関する費用........46
10.10.2　健康被害に関する補償........46
11. 品質管理及び品質保証........47
11.1　研究の記録........47
11.2　モニタリング........47
11.2.1　施設訪問モニタリング........47
11.2.2　監査........47
11.3　研究実施計画書からの逸脱・違反........47
12. データの公表........48
12.1　公開データベースへの記録........48
12.2　データの公表........48
12.3　Layperson Summary（LPS）........48
13. 研究全体の中止・中断・終了........49
13.1　研究全体の終了........49
13.2　研究全体の中止・中断........49
13.3　総括報告書........49
13.4　研究終了時の手続き........49
14. 研究成果の帰属........49
15. 研究実施体制........49
15.1　実施責任組織........49
15.1.1　研究代表医師........49
15.2　共同研究機関........50
15.2.1　共同研究機関の長........50
15.2.2　共同研究機関統括責任者........50
15.2.3　共同研究機関責任者........51
15.3　資金提供者........51
15.4　研究事務局........51
15.5　実施医療機関........51
15.6　統計解析責任者........52

15.7　支援事務局 52
15.8　モニタリング担当機関 52
15.9　データマネジメント担当機関 52
15.10 監査業務 52
15.11 調整管理実務担当者 53
15.12 画像解析機関 53
15.13 研究実行委員 53
15.14 医学専門アドバイザー（画像解析） 53
16. 参考文献 54

添付資料一覧

Appendix 1　研究実施スケジュール 57
Appendix 2　eCRF の入力項目 59

別紙一覧

別紙1　実施医療機関及び研究責任医師一覧
別紙2　過量投与/投薬過誤／薬物乱用／薬物誤用に関する調査報告書
別紙3　妊娠症例に関する調査報告書
別紙4　出生児に関する調査報告書

研究実施計画書要約

標題：	網膜中心静脈閉塞症（CRVO）に伴う黄斑浮腫患者に対するファリシマブを用いた modified treat-and-extend（mTAE）レジメンの検討<br>略称：RVO treatment Strategy with modified Treat And extend Regimen of faricimab（RVOSTAR）study
研究実施計画書番号：	CMA-0210
版番号：	第1.0版
研究薬：	ファリシマブ（遺伝子組換え）
適応：	未治療の網膜中心静脈閉塞症又は半側網膜静脈閉塞症に伴う黄斑浮腫患者
実施責任組織：	三重大学大学院医学系研究科　臨床医学系講座

I　目的及び評価項目

(1) 目的

未治療の網膜中心静脈閉塞症（CRVO）又は半側網膜静脈閉塞症（HRVO）に伴う黄斑浮腫患者を対象とし、実臨床に即した投与レジメン（mTAE）を用いたファリシマブの有効性及び投与間隔の延長を評価するとともに、有効性及び投与間隔に関係する因子を探索的に評価する。

(2) 評価項目

① 主要評価項目

- Week（W）72 での最高矯正視力（BCVA）のベースラインからの変化量
  各計測時点での BCVA は小数視力で測定し、BCVA の変化量は logarithm of the minimum angle of resolution（logMAR）に換算して計算する。

② 副次的評価項目

評価項目ごとに規定された W72 までの各時点（統計解析計画書［SAP］に規定）における以下の項目を評価する。

- BCVA 及び BCVA のベースラインからの変化量（logMAR）
- ベースラインから logMAR で 0.3 以上の改善がみられた患者の割合
- ベースラインから logMAR で 0.3 以上の悪化がみられなかった患者の割合
- 中心領域網膜厚（CST）及び CST のベースラインからの変化量
- ファリシマブの投与間隔ごとの患者の割合
- ファリシマブの平均投与回数

また、W72 時点の以下の項目を評価する。

- ファリシマブ導入後の観察期の日数
- ファリシマブ導入後、観察期にファリシマブの追加投与がなかった患者の割合

評価項目ごとに規定された W72 までの各時点（SAP に規定）における以下の項目を、ベースライン時の網膜虚血の有無によりそれぞれ評価する。ベースライン時の網膜虚血は Early Treatment Diabetic Retinopathy Study（ETDRS）の 7 方向眼底写真（又はそれに準じた範囲）による 10 乳頭面積以上の毛細血管閉塞と定義し、それ以外を非網膜虚血と定義する。

- BCVA 及び BCVA のベースラインからの変化量

- CST 及び CST のベースラインからの変化量
- ファリシマブの投与間隔ごとの患者の割合
- ファリシマブの平均投与回数

③ 探索的評価項目

評価項目ごとに規定された W72 までの各時点における以下の項目を評価する。

- 黄斑浮腫の消失が認められた患者の割合
- 網膜内滲出液（IRF）を認めない患者の割合、網膜下液（SRF）を認めない患者の割合、及びその両方を認めない患者の割合
- Fluid volume（IRF、SRF）及びそのベースラインからの変化量
- 網膜虚血を認めない患者の割合（フルオレセイン蛍光眼底造影［FA］による評価）
- 黄斑及び網膜全域の虚血無灌流（毛細血管の喪失）領域、及びそのベースラインからの変化（FA による評価）
- 非虚血型 CRVO から虚血型へ移行した患者の割合（FA による評価）
- 黄斑及び網膜全域の血管漏出面積並びにそのベースラインからの変化量（FA による評価）
- 血管漏出を認めない患者の割合（FA による評価）
- 網膜浅層血管網、網膜深層血管網及び網膜全層血管網における血管密度のベースラインからの変化（OCT-A による評価）
- ベースラインのパラメータと BCVA のベースラインからの変化量、CST のベースラインからの変化量、投与回数、投与間隔、及びその他の有効性パラメータの関係※
- W72 の投与間隔に影響を与える因子の探索※
- W24 までの CST の変動（W4 から W24 の CST 値に基づく患者ごとの標準偏差［SD］）と、BCVA、投与間隔、及びその他の有効性パラメータとの関係※
- 導入期でファリシマブを複数回投与した患者と 1 回のみ投与した患者におけるベースライン時及び W72 時のパラメータの比較※
- ファリシマブ投与開始後の Best BCVA（小数視力の最高値）が得られる日までの日数
- ファリシマブ投与開始後の Best CST（CST の最低値）が得られるまでの日数
- Day 1 から W24 までの BCVA による曲線下面積（AUC）
- Loss of peak vision（Best BCVA から logMAR で 0.1、0.2、及び 0.3 以上の悪化）の割合とその要因となる背景因子の探索※
- ベースライン時に黄斑上膜がない患者での新規黄斑上膜発生数

※解析する因子及び解析方法は SAP に規定する。

④ 安全性評価項目

- 眼の有害事象の発現率及び重症度
- 眼以外の有害事象の発現率及び重症度

II 研究デザイン

本研究は、未治療の CRVO 又は HRVO に伴う黄斑浮腫患者を対象とし、ファリシマブの投与レジメン（mTAE レジメン）の有効性及び安全性を検討する、非遮蔽、単群の多施設共同前向き介入研究である。本研究は臨床研究法対象の特定臨床研究である。

本研究に参加する患者はファリシマブ 6.0 mg の硝子体内投与を受ける。参加期間は 72 週間で、全患者を対象に Day 1 にてファリシマブを投与し、以降、導入期、観察期、及び維持期の各基準に従ってファリシマブの投与又は観察を行う。来院間隔については、Day 1 から W24 までは 4 週ごとに、W24 以降は、W36（観察期の患者のみ）、W52、及び W72 で規定来院するが、投与間隔に応じた頻度で来院する。

1) 導入期

ファリシマブ投与開始から、最初に黄斑浮腫の消失が確認されるまでの期間（黄斑浮腫の消失が確認されない場合は W20 までの期間）を導入期とする。

全患者を対象に Day 1 で 1 回ファリシマブを投与し、以降は 4 週ごとに 1 回投与する。W4 以降、投与前に黄斑浮腫の消失が確認された場合、ファリシマブを投与せず、観察期に移行する。なお、当該来院日の CST 値を、reference CST とする。

導入期のファリシマブの投与回数は最大 6 回までとする。W20 まで 6 回連続投与しても黄斑浮腫の消失が確認できない場合は､次回投与日を W24 として観察期を経ずに維持期に移行する。この場合、Day 1 から W20 までに得られた CST の最低値を reference CST とする。

なお、黄斑浮腫の消失は、本研究実施計画書で定める基準に基づいて研究責任医師又は研究分担医師が判断するが、基準を満たしていない場合であっても、判断理由を記録した上で黄斑浮腫の消失とみなすことができる。

2) 観察期

黄斑浮腫の消失が確認された来院日から再び疾患活動性が確認される日までを観察期とする。

観察期には、ファリシマブを投与せず、W24 までは 4 週ごとに、W24 以降は疾患活動性が認められない限り W36、W52 及び W72 の規定来院日にモニタリングを行う。

来院時に疾患活動性が確認された場合は、同日又は当該来院の許容範囲（規定来院日の-7 日～+14 日）内にファリシマブを投与し、維持期に移行する。疾患活動性の基準を満たしていない場合であっても、前回の来院と比し大幅な視力低下（例：小数視力 0.6→0.3）を伴い、かつ、治療が必要な浮腫があると研究責任医師又は研究分担医師が判断した場合は「疾患活動性あり」と判断し、ファリシマブの投与を許容する。逆に、疾患活動性の基準を満たしている場合でも、自覚症状を認めず臨床的に意味のある変動ではないと研究責任医師又は研究分担医師が判断した場合は「疾患活動性なし」と判断することを許容する。研究責任医師又は研究分担医師はこれらの判断理由を記録する。

また、研究責任医師又は研究分担医師が必要と判断した場合は、規定来院の間でも規定外来院にてモニタリングを行う。規定外来院時に疾患活動性が確認された場合は、ファリシマブを投与し、維持期に移行する。

3) 維持期

観察期の期間と同じ期間（観察期を経ず維持期に移行した場合は 4 週）を、維持期における最初の投与間隔と設定する。維持期の投与間隔は以下の調整基準に沿って 4 週ごとに調整する。投与間隔の調整幅は±4 週であり、最短の投与間隔は 4 週とするが、最長については設定しない。この調整基準とは異なる調整が必要であると研究責任医師又は研究分担医師が判断した場合は、判断理由を記録した上で投与間隔を調整する。

Reference CST からの CST の変化率（%）	投与間隔の調整
+10%未満	延長（+4週）
+10%以上+20%未満	維持（±0週）
+20%以上	短縮（-4週）

疾患活動性の基準を満たしていない場合であっても､前回の来院と比し大幅な視力低下（例：小数視力 0.6→0.3）を伴い、かつ、治療が必要な浮腫があると研究責任医師又は研究分担医師が判断した場合は「疾患活動性あり」と判断し、投与間隔を短縮する。また、疾患活動性の基準を満たしている場合でも、自覚症状を認めず臨床的に意味のある変動ではないと研究責任医師又は研究分担医師が判断した場合は、判断理由を記録した上で「疾患活動性なし」とし投与間隔を延長又は維持できる。

研究スケジュール概略図を以下に示す。

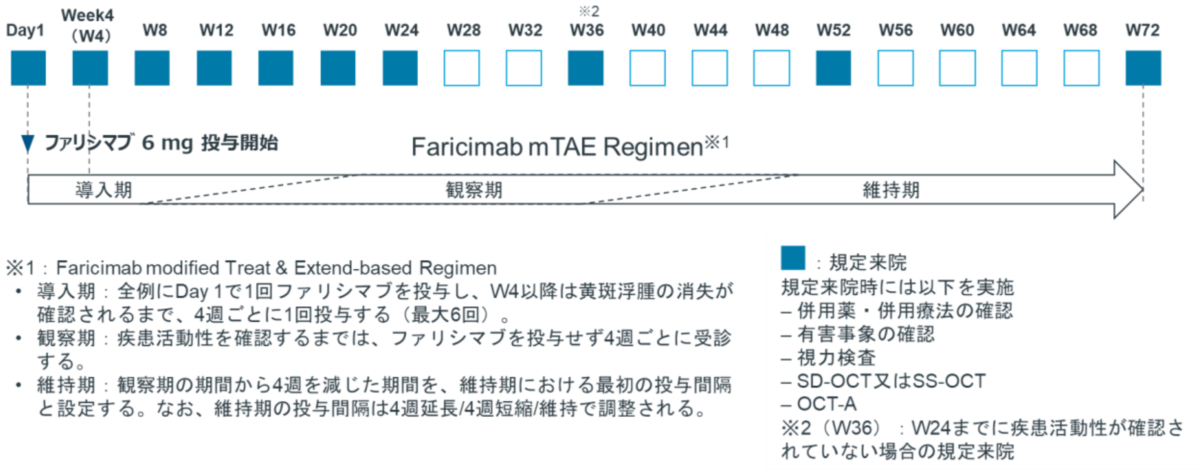

III　予定登録数

72 例と設定した。なお、HRVO 患者の登録はこのうち 14 例以下とする。詳細は「3.5 目標症例数の設定根拠」参照。

IV　対象

下記のすべての選択基準に該当し、すべての除外基準に該当しない患者を本研究の対象とする。

(1) 選択基準

1) 一般的な選択基準

① 署名した同意文書を提出する意思と能力のある患者
② 同意文書への署名時点で 18 歳以上の患者
③ 予定されるすべての来院と検査を受けることが可能であり、その意思がある患者

2) 対象眼の選択基準

本研究では、片眼のみを対象眼に指定する。両眼が対象となりうる場合は、研究責任医師または研究分担医師の判断で対象眼を選択する。

① スクリーニング来院前 4 カ月以内に、スペクトルドメイン型光干渉断層撮影（SD-OCT）又は波長掃引型光干渉断層撮影（SS-OCT）の画像に基づき中心窩を侵している CRVO 又は HRVO に伴う黄斑浮腫と認められた患者
なお、罹患静脈より漏出した網膜出血や拡張した毛細血管床、拡張した静脈系、又は、その他の網膜静脈閉塞症（RVO）の生物顕微鏡的所見（新生血管又は硝子体出血）が網膜の全体に認められる場合には CRVO、2 分の 1 の範囲に認められる場合には HRVO とする。
※設定根拠：COMINO 試験に準じて設定した。
② CRVO 又は HRVO に伴う黄斑浮腫の治療（抗 VEGF 硝子体内注射、ステロイドを含む）を受けたことがない患者
③ Day 1 の投与前に実施する視力検査で、小数視力 0.5～0.05 の患者
※設定根拠：COMINO 試験の設定（ETDRS 視力検査に基づき 73～19 文字）に準じ、小数視力として 0.5～0.05 が適切と判断した。
④ スクリーニング来院時の CST が以下のいずれかに該当する患者
– Spectralis SD-OCT で 325 μm 以上
– Cirrus SD-OCT 又は Topcon SD-OCT 若しくはその他これに準じる OCT で 315 μm 以上

※設定根拠：適切に評価を行うため

⑤ 中間透光体が十分にクリアで瞳孔は十分に散大し、確定診断のための良質な網膜画像の撮影が可能な患者
※設定根拠：適切に評価を行うため

(2) 除外基準

以下のいずれかに該当する患者は本研究の対象から除外する。

1) 一般的な除外基準

① Day 1 で全身性感染の疑い又は活動性の全身性感染に対して全身治療を受けている患者
② Day 1 前 6 カ月以内に脳卒中（脳血管発作）又は心筋梗塞を発症した患者
③ Day 1 にコントロール不良の高血圧である患者（安静時の収縮期血圧 180 mmHg 超及び／又は拡張期血圧 110 mmHg 超と定義）。スクリーニング期間中の最初の測定でこれらの値を超えた場合、同日又はスクリーニング期間中の別の日に再測定してもよい。
④ スクリーニング前 12 カ月以内の活動性の癌を有する患者。ただし適切に治療された子宮頚部上皮内癌、非黒色腫皮膚癌、グリーソンスコアが 6 以下で前立腺特異抗原が 12 カ月を超えて安定している前立腺癌を除く。
⑤ スクリーニング前 1 カ月以内に何らかの重大な疾患又は重大な外科的処置を経験した患者
⑥ スクリーニング前 1 カ月以内にステロイドの全身投与（例：経口投与又は注射）を受けた患者
⑦ ファリシマブの使用が禁忌となる、研究結果の解釈に影響を及ぼす可能性がある、治療合併症のリスクが高まることが十分に疑われると研究責任医師又は研究分担医師が判断したその他の疾患、代謝機能不全、理学的検査所見又は臨床検査所見の既往又は合併を有する患者
⑧ 妊娠中若しくは授乳中の女性
⑨ 妊娠可能な女性*の場合、研究治療期間中及び研究薬の最終投与日から少なくとも 3 カ月間にわたって、禁欲（異性との性交を行わない）の継続又は 1 年間の失敗率が 1%未満**の許容される避妊法の使用に同意できない患者。

* 初経後で、閉経後状態（連続12カ月以上にわたり無月経で、閉経以外の原因が認められない状態）に達しておらず、手術（卵巣、卵管及び／又は子宮の摘出）又は研究責任医師又は研究分担医師が判断した他の原因（例：ミュラー管形成不全）により永久的な不妊になっていない女性を妊娠可能とみなす。この規定に従い、片側卵管結紮の女性は妊娠可能とみなす。

** 年間避妊失敗率が1%未満の避妊法の例としては、両側卵管結紮術、男性不妊手術、排卵を阻害するホルモン避妊薬、ホルモン放出子宮内器具、銅付加子宮内器具等が挙げられる。性的禁欲の信頼性については、臨床試験の期間及び各患者の好みや通常のライフスタイルに関して評価すべきである。周期的禁欲（カレンダー法、排卵日法、徴候体温法、排卵後法等）及び膣外射精は十分な避妊法ではない。

⑩ 生物学的製剤に高度のアレルギー反応又はアナフィラキシー反応の既往がある患者、又はファリシマブの成分、研究手順に用いる薬剤（フルオレセインを含む）、散瞳点眼薬、研究期間中に使用する麻酔薬及び抗菌薬に対して既知の過敏症がある患者
⑪ Day 1 前 3 カ月以内に薬剤（ビタミン及びミネラルを除く）又は機器を用いて治療を行う眼科領域の臨床試験に参加していた患者
⑫ 以下に示す併用禁止薬剤又は治療を継続的に使用する必要のある患者
- 抗血管内皮増殖因子（vascular endothelial growth factor：VEGF）薬の全身投与
- 黄斑浮腫の原因となることが知られている全身性の薬剤（フィンゴリモド、タモキシフェン等）
- 対象眼への抗 VEGF 薬（ファリシマブ以外）の硝子体内投与

– 対象眼へのステロイドの硝子体内、眼周囲（テノン嚢下）、又は習慣的な眼への局所投与
– 対象眼へのベルテポルフィン（ビスダイン®）治療
– 対象眼へのマイクロパルス及び局所又は格子状光凝固術
– その他の試験的な治療（ビタミン及びミネラルによるものを除く）

※設定根拠：
①～⑩、⑫　安全性の懸念がある又は安全性評価に影響を与えるため
⑪　適切な評価が出来ない又は有効性評価に影響を与えるため

2) 対象眼に適用される除外基準

① スクリーニングの 4 カ月以上前に診断された CRVO 又は HRVO に伴う黄斑浮腫又は CRVO 又は HRVO に伴う持続性黄斑浮腫の既往を有する患者
② 網膜剥離又は黄斑円孔（Stage 3 又は 4）の既往を有する患者
③ 研究責任医師又は研究分担医師により、現在の眼の病態が CRVO 又は HRVO に伴う黄斑浮腫以外の原因で対象眼に不可逆的な視力障害をもたらしつつある又はもたらすことが予想されると判断された患者（例：虚血性黄斑症、アーヴァイン・ガス症候群、中心窩萎縮、中心窩線維症、色素異常、密な中心窩下硬性白斑又はその他の網膜以外の状態）
④ 対象眼の牽引性網膜剥離、全層黄斑円孔、硝子体黄斑牽引症又は中心窩癒着若しくは黄斑構造破壊を伴う網膜上膜を認める患者
⑤ 研究責任医師又は研究分担医師により、中等度の非増殖糖尿病網膜症、より不良の増殖糖尿病網膜症、糖尿病黄斑浮腫（DME）、新生血管を伴う加齢黄斑変性（nAMD）患者及び地図状萎縮又は近視性脈絡膜新生血管と診断された患者
⑥ 活動性ルベオーシス、隅角新生血管、血管新生緑内障を有する患者
⑦ Day 1 前 3 カ月以内に白内障手術を受けたか、イットリウム・アルミニウム・ガーネット（YAG）レーザー後嚢切開による白内障手術合併症の治療を受けた患者
⑧ その他の眼内手術（例：経毛様体扁平部硝子体切除術、強膜バックル、緑内障手術、角膜移植、又は放射線療法）を受けた患者
⑨ Day 1 より前に対象眼に対して黄斑レーザー（局所／格子状）又は汎網膜光凝固術を受けたか、Day 1 の治療開始から 3 カ月以内に汎網膜光凝固術を受ける予定がある患者
⑩ 過去に光線力学的療法（PDT）、レーザー、経瞳孔的温熱療法又は sheathotomy 等の網膜硝子体手術による介入を受けた患者
⑪ 黄斑浮腫、糖尿病黄斑浮腫及び新生血管を伴う加齢黄斑変性を含む黄斑部新生血管又は硝子体黄斑界面異常に対する治療として、硝子体内投与（例：抗 VEGF 薬、ステロイド、組織プラスミノゲン活性化因子、C_3F_8、SF_6、空気）、眼周囲注射等の治療を過去に受けたか、現在受けている患者
⑫ 他の網膜疾患に対する眼周囲への薬物療法又は硝子体内注射（抗 VEGF 薬を含む）の治療歴がある患者

※設定根拠：
①～⑫　有効性・安全性の適切な評価が出来ないため

3) 両眼に適用される除外基準

① いずれかの眼に特発性又は免疫介在性ぶどう膜炎の既往のある患者
② Day 1 にいずれかの眼に活動性眼炎症、又は眼及び眼周辺部での感染の疑い又は活動性の感染がある患者

※設定根拠：
①　有効性・安全性の適切な評価が出来ないため
②　安全性の懸念あるいは安全性評価に影響を与えるため

V　研究の終了と研究期間

本研究は、総括報告書の概要を臨床研究等提出・公開システム（jRCT）に登録し、公表した日をもって終了日とする。

VI　研究薬

一般名：ファリシマブ（遺伝子組換え）
販売名：バビースモ硝子体内注射液 120 mg/mL

VII　観察・評価項目及び観察スケジュール

Appendix 1 参照。

VIII 統計手法

(1) 主要解析

①目標症例数の設定

目標症例数　：72例（HRVO 患者の登録はこのうち14例以下とする）
主要評価項目：W72における BCVA のベースラインからの変化量（logMAR）

ファリシマブの CRVO 又は HRVO に伴う黄斑浮腫を対象とした臨床試験（COMINO 試験）の結果に基づいて、主要評価項目である W72 における BCVA のベースラインからの変化量の真値を 16.9 文字、SD を 16.45 と仮定した場合、主要評価項目の点推定値が真値より 3 文字以上下回らない確率が 90%以上となる症例数は 50 例である。COMINO 試験での脱落率は 10%であったが、本研究では薬剤費が患者負担であり、通院にかかる負担も大きいことから、W72 までの脱落率 30%と見込み、症例数を 72 例と設定した。

②主要評価項目の解析

本研究の有効性の主要評価項目は、W72 における BCVA のベースラインからの変化量である。BCVA はランドルト環を用いて 5 m の距離で測定した小数視力を logMAR に換算した値とする。

BCVA は、経時測定データに対する混合効果モデル（MMRM）を用いて解析する。モデルには、固定効果として来院（カテゴリー変数）及びベースラインの BCVA（連続変数）を含め、患者内誤差のモデリングには無構造の共分散構造を仮定する。モデルの収束に問題がある場合は、Compound Symmetry 又は AutoRegressive(1)共分散構造を用いる。欠測のメカニズムはランダムな欠測を仮定する。データの研究利用の拒否を含む全同意撤回症例については、該当症例を評価対象から除外する。

(2) 中間解析

全例で投与開始後 24 週時点における BCVA のデータが得られた時点で、研究結果の学会等での公表を目的に中間解析を実施する。その他、必要に応じて追加の中間解析を行うことがある。詳細は SAP に記載する。

IX　問合せ先

研究代表医師　: 三重大学大学院医学系研究科　臨床医学系講座　眼科学
教授　近藤　峰生

研究事務局　: 三重大学大学院医学系研究科　臨床医学系講座　眼科学
教授　近藤　峰生
〒514-8507　三重県津市江戸橋二丁目174
Tel. 059-232-1111（代表）

共同研究機関　: 中外製薬株式会社
〒103-8324　東京都中央区日本橋室町2-1-1
Tel. 03-3281-6611

支援事務局　: IQVIA サービシーズ ジャパン合同会社
〒108-0074　東京都港区高輪4-10-18 京急第1ビル
Tel. 03-6859-9500

略語及び用語の定義

略語	英語名	和名
Ang-1	angiopoietin-1	アンジオポエチン-1
Ang-2	angiopoietin-2	アンジオポエチン-2
AUC	area under the curve	曲線下面積
BCVA	best corrected visual acuity	最高矯正視力
BRVO	branch retinal vein occlusion	網膜静脈分枝閉塞症
CFP	color fundus photograph	カラー眼底写真
CRB	certified review board	認定臨床研究審査委員会
CRVO	central retinal vein occlusion	網膜中心静脈閉塞症
CST	central subfield thickness	中心領域網膜厚
eCRF	electronic case report form	電子症例報告書
DME	diabetic macular edema	糖尿病黄斑浮腫
EDC	electronic data capture	電子データ収集
ETDRS	Early Treatment Diabetic Retinopathy Study	—
FA	fluorescein angiography	フルオレセイン蛍光眼底造影
Fab	fragment antigen-binding	抗原結合フラグメント
FAS	full analysis set	最大の解析対象集団
Fc	fragment crystallizable	フラグメント結晶化可能
FcRn	Fc receptor	胎児性 Fc 受容体
HBV	hepatitis B virus	B 型肝炎ウイルス
HCV	hepatitis C virus	C 型肝炎ウイルス
HIV	human immunodeficiency virus	ヒト免疫不全ウイルス
HRVO	hemispherical retinal vein occlusion	半側網膜静脈閉塞症
IgG	immunoglobulin G	免疫グロブリン G
IRF	intraretinal fluid	網膜内滲出液
jRCT	Japan Registry of Clinical Trials	臨床研究等提出・公開システム
logMAR	logarithm of the minimum angle of resolution	—
LPS	layperson Summary	—
MMRM	mixed effect model for repeated measure	経時測定データに対する混合効果モデル
mTAE	modified Treat and Extend	—
nAMD	neovascular age-related macular degeneration	新生血管を伴う加齢黄斑変性
OCT	optical coherence tomography	光干渉断層撮影
OCT-A	optical coherence tomography-angiography	OCT アンギオグラフィー
PDT	photodynamic therapy	光線力学的療法
PPS	Per-protocol set	Per-protocol 集団
PRN	*pro re nata*	必要時投与
PRP	panretinal photocoagulation	汎網膜光凝固術
RVO	retinal vein occlusion	網膜静脈閉塞症
SAP	statistical analysis plan	統計解析計画書
SD	standard deviation	標準偏差

略語	英語名	和名
SD-OCT	spectral-domain optical coherence tomography	スペクトルドメイン型光干渉断層撮影
SRF	subretinal fluid	網膜下液
SS-OCT	swept source optical coherence tomography	波長掃引型光干渉断層撮影
TAE	treat-and-extend	—
Tie-2	tyrosine kinase with immunoglobulin and epidermal growth factor homology domains-2	—
VEGF	vascular endothelial growth factor	血管内皮増殖因子
VEGF-A	vascular endothelial growth factor A	血管内皮増殖因子 A
YAG	Yttrium Aluminum Garnet	イットリウム・アルミニウム・ガーネット

用語	定義
黄斑浮腫の消失	Spectralis スペクトルドメイン型光干渉断層撮影（SD-OCT）で測定した CST が 325 μm 未満、又は Cirrus SD-OCT、Topcon SD-OCT 若しくはその他これらに準じる OCT で測定した CST が 315 μm 未満（これを基準として研究責任医師又は研究分担医師が判断する）
疾患活動性	reference CST に対し CST が 20%以上増加（これを基準として研究責任医師又は研究分担医師が判断する）
reference CST	ファリシマブ投与開始後、最初に黄斑浮腫の消失を認めたときの CST 値、又はファリシマブの 6 回連続投与で黄斑浮腫の消失を認めなかった場合はそれまでの CST の最低値
導入期	ファリシマブ投与開始日から、最初に黄斑浮腫の消失を認めた日又は黄斑浮腫の消失が認められない場合は Week 20まで
観察期	ファリシマブ投与開始後、最初に黄斑浮腫の消失を認めた日から疾患活動性を認めた日までの期間
維持期	導入期／観察期終了後のファリシマブ維持投与期間

1. 背景

1.1 対象疾患の背景

RVO は、網膜内や視神経内の静脈が閉塞し、網膜の正常な血流が制限され、VEGF を中心としたサイトカインの発現亢進と分泌が誘導され、眼底出血や黄斑浮腫、視力低下等が起こる疾患である[1]。

RVO は閉塞部位を基に、CRVO、HRVO 又は網膜静脈分枝閉塞症（BRVO）に分類され[2]、一般的には、閉塞部位が網膜の一部（約1/4）である BRVO よりも網膜全体に及ぶ CRVO でその影響範囲が大きく、重症度も高い[3, 4]。

RVO は、世界で2008年時点に1640万人が罹患した疾患であり、視力を脅かす網膜血管障害としては、糖尿病性網膜症に次いで2番目に多い疾患である[5]。日本における RVO の有病率に関しては、1998年時点の40歳以上を対象にした調査で、2.1%で、うち2.0%が BRVO、0.2%は CRVO であったことが報告された[6]。

RVO に伴う黄斑浮腫は、視力喪失の原因にもなっている。RVO に伴う黄斑浮腫の治療を巡っては、現在、抗 VEGF 薬の硝子体内注射が第一選択となっており[2]、一部の患者では黄斑レーザー[7]やステロイドの硝子体内注射[8]が使用されている。第一選択となっている抗 VEGF 薬の硝子体内注射に関しては、VEGF のみならず他の疾患経路を標的とすることで、治療結果やその持続性が向上する可能性があるとされている[9]。アンジオポエチン-2（Ang-2）は、tyrosine kinase with immunoglobulin and epidermal growth factor homology domains-2（Tie-2）との結合をめぐってアンジオポエチン-1（Ang-1）と競合し、Ang-1及び Tie シグナル伝達の血管安定性促進効果を阻害する[10-12]。また、Ang-2は網膜疾患において、サイトカイン誘発性血管漏出に重要な役割を果たすことが前臨床試験の知見から示されている[13]。Ang-2と VEGF は、血管の不安定性を促進し、血管漏出、炎症及び血管新生の増加といった疾患の進行につながり[12]、RVO に伴う黄斑浮腫の病態生理に関与する重要な因子とされている。これらを二重阻害することで、血管内皮増殖因子 A（VEGF-A）阻害剤のみの場合と比較して、より持続的に網膜血管を安定させることができると考えられる[14-16]。

1.2 ファリシマブの背景

ファリシマブ（遺伝子組換え）（以降、ファリシマブ）は、F. Hoffmann-La Roche, Ltd.（以降、ロシュ社）で創製された、VEGF-A 及び Ang-2に選択的に結合するヒト化二重特異性免疫グロブリン G（IgG）1抗体である。VEGF-A に結合する抗原結合フラグメント（Fab）ドメインは高い親和性で VEGF-A に結合し、Ang-2に結合する Fab ドメインは高い親和性及び選択性で Ang-2に結合する。フラグメント結晶化可能（Fc）ドメインはエフェクター細胞上の Fcγ 受容体及び胎児性 Fc 受容体（FcRn）へ結合しないよう改変されているため、硝子体内注射後の全身曝露量が減少している可能性がある。ファリシマブの有効性及び安全性は、nAMD[17]及び DME[18]患者を対象とした臨床試験で確認され、これらの臨床試験成績に基づき、日本では2022年3月に中心窩下脈絡膜新生血管を伴う加齢黄斑変性及び糖尿病黄斑浮腫を適応症として承認されている。また、2024年3月に、BRVO、CRVO 及び HRVO 患者を対象とした臨床試験の成績に基づき、網膜静脈閉塞症に伴う黄斑浮腫の適応追加についても承認されている[19]。

1.3 研究計画設定の根拠

RVO の黄斑浮腫に対する抗 VEGF 薬の国際共同第 III 相試験[20-26]では、6 回の毎月投与により著明な視力改善効果が示され、抗 VEGF 薬の硝子体内注射が第一選択となっている。抗 VEGF 薬は通常長期の投与が必要とされ、その最適な治療間隔を決定するために、これまで固定投与、必要時投与（PRN）レジメン及び treat-and-extend（TAE）レジメンなどのさまざまな投与方法が検討されてきた。しかし、実臨床では、長期の治療期間中に治療から脱落する患者の割合は高く、治療成績が臨床試験での結果より悪いことが明らかになっている[27]。抗 VEGF 薬のアドヒアラ

ンス低下の要因として、頻回の治療による患者の経済的及び心理的負担が大きいことが挙げられている[28]。一方で、6 回投与後の維持期は PRN 法で実施されており、BRVO では 1 年以上は視力維持できているが[21, 26]、CRVO では改善した視力が低下傾向であることが課題となっている[23-26]。このため、より患者負担の少ない新しい投与レジメンやその効果に関連する因子の検討が必要となっている。

本研究では、modified treat-and-extend（mTAE）レジメンによるファリシマブの投与レジメンを検討する。mTAE は従来の TAE 法の前に観察期を設定することで、患者毎の再発間隔を見極めた上で TAE を開始するというものである。本投与レジメンにより、改善された視力を維持しながら投与間隔の延長することができれば、現在の clinical gap の解消につながることが期待される。

ファリシマブの BRVO、CRVO 及び HRVO に伴う黄斑浮腫を対象とした臨床試験（BALATON 試験及び COMINO 試験）[29]では、RVO の黄斑浮腫に対する他の抗 VEGF 薬を用いた試験[20-26]と同様、4 週ごとに 6 回投与を行い、主要評価項目である W24 時の視力改善において、対照群で用いたアフリベルセプトに対する非劣性が示された。また、24 週以降の維持期は、TAE レジメンに従い、BRVO のみならず、CRVO 又は HRVO においても 72 週まで視力改善効果が維持された[30]。

2. 目的及び評価項目

2.1 目的

未治療の CRVO 又は HRVO に伴う黄斑浮腫患者を対象とし、実臨床に即した投与レジメン（mTAE）を用いたファリシマブの有効性及び投与間隔の延長を評価するとともに、有効性及び投与間隔に関係する因子を探索的に評価する。

2.2 評価項目

有効性の評価については、以下を参照。

「2.2.1 主要評価項目」「2.2.2 副次的評価項目」「2.2.3 探索的評価項目」

安全性の評価については、「2.2.4 安全性評価項目」を参照。

本研究の「導入期」、「観察期」及び「維持期」の定義、並びに「黄斑浮腫の消失」、「疾患活動性」及び「reference CST」の定義については略語及び用語の定義を参照。

2.2.1 主要評価項目

- W72 での BCVA のベースラインからの変化量
  各計測時点での BCVA は小数視力で測定し、BCVA の変化量は logMAR に換算して計算する。

2.2.2 副次的評価項目

評価項目ごとに規定された W72 までの各時点（SAP に規定）における以下の項目を評価する。

- BCVA 及び BCVA のベースラインからの変化量（logMAR）
- ベースラインから logMAR で 0.3 以上の改善がみられた患者の割合
- ベースラインから logMAR で 0.3 以上の悪化がみられなかった患者の割合
- CST 及び CST のベースラインからの変化量
- ファリシマブの投与間隔ごとの患者の割合
- ファリシマブの平均投与回数

また、W72 時点の以下の項目を評価する。

- ファリシマブ導入後の観察期の日数
- ファリシマブ導入後、観察期にファリシマブの追加投与がなかった患者の割合

評価項目ごとに規定された W72 までの各時点（SAP に規定）における以下の項目を、ベースライン時の網膜虚血の有無によりそれぞれ評価する。ベースライン時の網膜虚血は ETDRS の 7 方向眼底写真（又はそれに準じた範囲）による 10 乳頭面積以上の毛細血管閉塞と定義し、それ以外を非網膜虚血と定義する。

- BCVA 及び BCVA のベースラインからの変化量
- CST 及び CST のベースラインからの変化量
- ファリシマブの投与間隔ごとの患者の割合
- ファリシマブの平均投与回数

2.2.3 探索的評価項目

評価項目ごとに規定された W72 までの各時点における以下の項目を評価する。

- 黄斑浮腫の消失が認められた患者の割合
- IRF を認めない患者の割合、SRF を認めない患者の割合、及びその両方を認めない患者の割合
- Fluid volume（IRF、SRF）及びそのベースラインからの変化量
- 網膜虚血を認めない患者の割合（FA による評価）
- 黄斑及び網膜全域の虚血無灌流（毛細血管の喪失）領域、及びそのベースラインからの変化（FA による評価）
- 非虚血型 CRVO から虚血型 CRVO へ移行した患者の割合（FA による評価）
- 黄斑及び網膜全域の血管漏出面積並びにそのベースラインからの変化量（FA による評価）
- 血管漏出を認めない患者の割合（FA による評価）
- 網膜浅層血管網、網膜深層血管網及び網膜全層血管網における血管密度のベースラインからの変化（OCT-A による評価）
- ベースラインのパラメータと BCVA のベースラインからの変化量、CST のベースラインからの変化量、投与回数、投与間隔、及びその他の有効性パラメータとの関係※
- W72 の投与間隔に影響を与える因子の探索※
- W24 までの CST の変動（W4 から W24 の CST 値に基づく患者ごとの SD）と、BCVA、投与間隔、及びその他の有効性パラメータとの関係※
- 導入期でファリシマブを複数回投与した患者と 1 回のみ投与した患者におけるベースライン時及び W72 時のパラメータの比較※
- ファリシマブ投与開始後の Best BCVA（小数視力の最高値）が得られる日までの日数
- ファリシマブ投与開始後の Best CST（CST の最低値）が得られるまでの日数
- Day 1 から W24 までの BCVA による AUC
- Loss of peak vision（Best BCVA から logMAR で 0.1、0.2、及び 0.3 以上の悪化）の割合とその要因となる背景因子の探索※
- ベースライン時に黄斑上膜がない患者での新規黄斑上膜発生数

※解析する因子及び解析方法は SAP に規定する。

2.2.4 安全性評価項目

- 眼の有害事象の発現率及び重症度
- 眼以外の有害事象の発現率及び重症度

3. 本研究の概要

3.1 本研究の種類

本研究は、研究の目的で検査、投薬その他の診断又は治療のための医療行為の有無及び程度を制御する、介入を伴う、非遮蔽かつ単群の多施設共同臨床研究である。なお、本研究は臨床研究法対象の特定臨床研究である。

本研究は「ヘルシンキ宣言」（日本医師会訳）*、「臨床研究法（平成 29 年法律第 16 号）」**、「臨床研究法施行規則（平成 30 年厚生労働省第 17 号）」**、及び本研究実施計画書を遵守して実施する。

* https://dl.med.or.jp/dl-med/wma/helsinki2013j.pdf

** https://www.mhlw.go.jp/stf/seisakunitsuite/bunya/0000163417.html

3.2 研究のデザイン

本研究は、未治療の CRVO 又は HRVO に伴う黄斑浮腫患者を対象とし、ファリシマブの投与レジメン（mTAE レジメン）の有効性及び安全性を検討する、非遮蔽、単群の多施設共同前向き介入研究である。本研究は臨床研究法対象の特定臨床研究である。

本研究に参加する患者はファリシマブ 6.0 mg の硝子体内投与を受ける。参加期間は 72 週間で、全患者を対象に Day 1 にてファリシマブを投与し、以降、導入期、観察期、及び維持期の各基準に従ってファリシマブの投与又は観察を行う。各期の手順については「5.2 プロトコール治療」に記載した。来院間隔については、Day 1 から W24 までは 4 週ごとに、W24 以降は W36（観察期の患者のみ）、W52、W72 を規定来院とした上で、mTAE レジメンに従って決定した投与間隔に応じた頻度で来院する。

研究スケジュール概略図を図 3.2-1 に示す。

図 3.2-1 研究スケジュール概略図

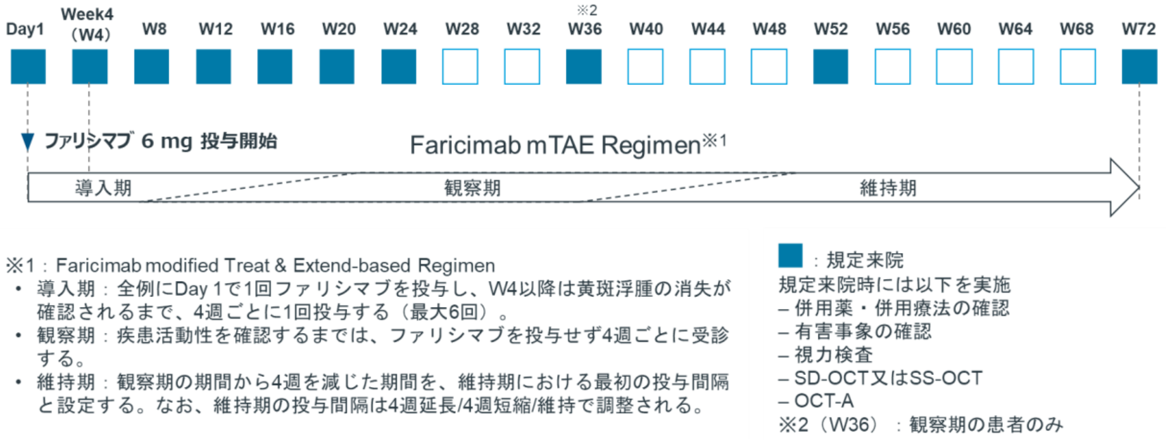

3.3 研究の終了と研究期間

本研究は、総括報告書の概要を jRCT に登録し、公表した日をもって終了日とする。

予定登録期間・予定追跡期間は、認定臨床研究審査委員会（CRB）への手続きに係る期間等を見込んで以下のように設定した。

- 予定登録期間：jRCT の公表日後、最初の症例登録日から 1 年 6 カ月
- 予定追跡期間：jRCT の公表日後、最後の症例登録日から 72 週間
- 報告書作成期間：追跡期間終了から 1 年
- 予定研究期間：jRCT 公表日から 4 年

3.4 研究デザインの設定根拠

RVO の黄斑浮腫に対する抗 VEGF 薬の国際共同第 III 相試験[21, 24-26]では、4 週ごとに連続 6 回投与を行い、著明な視力改善効果が示され、標準治療となっている。一方で、これまでの国際共同第 III 相試験では、6 回投与後の維持期は PRN レジメンで実施されており、BRVO では 1 年以上は視力維持できている[21, 26]が、CRVO では改善した視力が低下傾向[23-26]であり、課題となっている。

ファリシマブの BRVO、CRVO 及び HRVO に伴う黄斑浮腫を対象とした臨床試験（BALATON 試験及び COMINO 試験）[29]では、RVO の黄斑浮腫に対する他の抗 VEGF 薬を用いた試験[20-26]と同様、4 週ごとに 6 回投与を行い、主要評価項目である 24 週時の視力改善において、対照群のアフリベルセプトへの非劣性が証明された。また、24 週以降の維持期は、TAE レジメンに従い、BRVO のみならず、CRVO 又は HRVO においても 72 週まで視力改善効果が維持された[29]。

一方で、COMINO 試験によるファリシマブの平均投与回数は 72 週間で 11 回となっており、実臨床での実態[31]と比べると 2 倍以上である。また、投与間隔においても、ファリシマブの他疾患（nAMD、DME）での成績[17, 18]と比べると、COMINO 試験では投与間隔が 12 週以上の患者の割合が少なかった。これらの理由の一つとして、他疾患での試験と比べて厳しいレジメン（投与間隔をいったん短縮すると再延長ができない）であったことが考えられる。治療負担（薬剤費、通院回数等）は治療のアドヒアランス低下の大きな要因であるが[32]、実臨床における視力改善効果が十分でない点も課題であり、CRVO 又は HRVO を対象とし、国際共同第 III 相試験と同程度の視力改善効果を維持しつつ、実臨床で使用可能かつ、投与回数を減らせるファリシマブのレジメンの検討が求められている。

本研究で実施する mTAE レジメンでは、導入期の規定投与回数を設けず、黄斑浮腫の消失が認められるまで最大 6 回ファリシマブを投与し、黄斑浮腫の消失が認められた場合は観察期に移行し、経過観察する。その後、再投与基準を満たした場合は直ちに投与を再開し、観察期の期間と同じ期間を最初の投与間隔とする維持期に移行する。この mTAE レジメンは、導入期には患者の病勢に応じた投与回数、維持期には患者の病勢に応じた投与間隔で投与できることから、抗 VEGF 薬の過剰投与を防ぎ患者負担の軽減につながり、かつ、長期の視力維持に貢献すると考えられる。

3.5 目標症例数の設定根拠

目標症例数：72例（HRVO 患者の登録はこのうち14例以下とする）

CRVO 又は HRVO に伴う黄斑浮腫を対象としたファリシマブの臨床試験（COMINO 試験）の結果に基づいて、主要評価項目である W72における BCVA のベースラインからの変化量の真値を16.9文字、SD を16.45と仮定した場合、主要評価項目の点推定値が真値より3文字以上下回らない確率が90%以上となる症例数は50例である。COMINO 試験での脱落率は10%であったが[29]、本研究では薬剤費が患者負担であり、通院にかかる負担も大きいことから、W72までの脱落率を30%と見込み、症例数を72例と設定した。また、患者背景を COMINO 試験と揃えるため、HRVO 症例の登録数に上限を設けた。COMINO 試験での HRVO 症例の割合（ITT 集団中17.5%、日本部分集団中16.3%）に基づき、本研究では HRVO 症例割合が全体の20%を超えないように HRVO 症例数の上限を14例とした。

3.6 本研究の意義

抗 VEGF 薬の登場は、RVO の治療を大きく進歩させた。その一方、抗 VEGF 薬の頻回の硝子体内投与は患者負担が大きいため、実臨床では、病態の悪化時に再投与を行う PRN 治療が主流である。しかしながら、十分な治療を行わないことにより網膜の障害が徐々に進行し、不可逆的な視力障害に至る可能性がある。本研究で用いるファリシマブの mTAE レジメンによる治療の有

効性及び安全性が確認されれば、より患者負担が少なく視機能維持が可能な新たな治療オプションを提供することとなり、現在の clinical gap の解消につながることが期待される。

3.7 研究参加に伴い予想される利益と不利益

本研究では全例が 72 週間にわたってファリシマブによる治療を受けるため、CRVO 又は HRVO に伴う黄斑浮腫の改善が期待されるが、通常の診療を上回る利益はない。また、本研究の結果は、学術的成果として将来的に CRVO 又は HRVO に伴う黄斑浮腫の治療法の進歩につながる可能性がある。

一方、本研究に参加することにより、通常の診療より来院回数及び検査回数が増え、検査費用の負担が増えることがあるため、投与が予定されていない観察・検査目的での来院に対しては来院ごとに負担軽減費を支払う（「10.10.1 治療に関する費用」参照）。また、通常診療でファリシマブの投与を受ける場合と同様に、ファリシマブの添付文書に記載された副作用が発現する可能性がある。ファリシマブとの関連性が確認された副作用の一覧を表 3.7-1 に、また、本研究で実施する検査や処置により起こりうるリスクを表 3.7-2 に示す。

表 3.7-1　ファリシマブの副作用（2024年3月時点）[19]

**重大な副作用**	
眼障害<br>• 眼内炎症（ぶどう膜炎、硝子体炎等）（1.2%）<br>• 網膜色素上皮裂孔（0.2%）<br>• 眼内炎（頻度不明）<br>• 裂孔原性網膜剥離（頻度不明）<br>• 網膜裂孔（頻度不明）<br>脳卒中（0.3%）	
**その他の副作用**	
眼障害（1%未満）<br>• 眼圧上昇<br>• 硝子体浮遊物<br>• 高眼圧症<br>• 角膜擦過傷<br>• 眼痛<br>• 眼部不快感	• 結膜出血<br>• 白内障<br>• 硝子体剥離<br>• 眼充血<br>• 霧視<br>• 視力低下

表 3.7-2　検査や処置により起こりうるリスク

検査・処置	起こりうるリスク
硝子体内注射	• 注射部位からの細菌感染の可能性がある。感染予防のために使用する抗菌点眼薬が原因で、眼刺激、かゆみ、腫れ、又は充血が起こることがある。<br>• 抗凝固薬（例：アスピリンや類似薬［ワルファリン等］）を使用している場合、ファリシマブの注射に関連した眼球内出血を生じやすくする可能性がある。
眼圧測定（眼圧測定器を角膜に接触させて測定する場合）	• 点眼麻酔をする場合がある。

検査・処置	起こりうるリスク
FA	• 撮像影前に、フルオレセインを腕に注射する。穿刺による不快感が生じる可能性がある。<br>• 色素の注射により、静脈に炎症が起きたり、注射部位に発赤や腫れが生じたりすることがある。<br>• フルオレセインでみられる主な副作用は吐き気及び嘔吐であるが、ときにアレルギー反応、失神、呼吸困難又はショックが起こることがある。フルオレセインより、皮膚や尿が黄色になることがあるが、これは1日ほどで解消される。
ポピドンヨードの使用	• ファリシマブの注射を行う研究責任医師又は研究分担医師が感染予防のために使用するポピドンヨードにより、短時間、眼が焼け付いてひりひりする感じや刺激感が生じる可能性がある。
散瞳薬の使用	• 散瞳薬で眼がしみる場合がある。<br>• 散瞳薬を使用した後に、しばらく視界がぼやけることがある。この症状が消失するまでは、自動車の運転や機械の操作を行わないよう患者に指示する。
抗菌点眼薬の使用	• 研究責任医師又は研究分担医師の判断で、ファリシマブの注射の前後に感染予防のために抗菌点眼薬を使用する場合がある。<br>• 抗菌点眼薬の使用により、目の刺激、かゆみ、腫れ、赤みが生じる可能性がある。

有害事象については、「4.1.1 選択基準」、「4.1.2 除外基準」、「5 治療計画と治療変更基準」を設け、リスクを最小限にするとともに、重篤な有害事象や予期されない有害事象が生じた場合には、必要な対策が講じられる体制が取られることになっている。

4. 対象と方法

4.1 対象

下記のすべての選択基準に該当し、すべての除外基準に該当しない患者を本研究の対象とする。

4.1.1 選択基準

1) 一般的な選択基準

① 署名した同意文書を提出する意思と能力のある患者
② 同意文書への署名時点で 18 歳以上の患者
③ 予定されるすべての来院と検査を受けることが可能であり、その意思がある患者

2) 対象眼の選択基準

本研究では、片眼のみを対象眼に指定する。両眼が対象となりうる場合は、研究責任医師または研究分担医師の判断で対象眼を選択する。

① スクリーニング来院前 4 カ月以内に、SD-OCT 又は SS-OCT の画像に基づき中心窩を侵している CRVO 又は HRVO に伴う黄斑浮腫と認められた患者
なお、罹患静脈より漏出した網膜出血や拡張した毛細血管床、拡張した静脈系、又は、その他の RVO の生物顕微鏡的所見（新生血管又は硝子体出血）が網膜の全体に認められる場合には CRVO、2 分の 1 の範囲に認められる場合には HRVO とする。
※設定根拠：COMINO 試験に準じて設定した。
② CRVO 又は HRVO に伴う黄斑浮腫の治療（抗 VEGF 硝子体内注射、ステロイドを含む）を受けたことがない患者
③ Day 1 の投与前に実施する視力検査で、小数視力 0.5～0.05 の患者
※設定根拠：COMINO 試験の設定（ETDRS 視力検査に基づき 73～19 文字）に準じ、小数視力として 0.5～0.05 が適切と判断した。
④ スクリーニング来院時の CST が以下のいずれかに該当する患者
- Spectralis SD-OCT で 325 μm 以上
- Cirrus SD-OCT 又は Topcon SD-OCT、その他これらに準じる OCT で 315 μm 以上

※設定根拠：適切に評価を行うため。
⑤ 中間透光体が十分にクリアで瞳孔は十分に散大し、確定診断のための良質な網膜画像の撮影が可能な患者
※設定根拠：適切に評価を行うため。

4.1.2 除外基準

以下のいずれかに該当する患者は本研究の対象から除外する。

1) 一般的な除外基準

① Day 1 で全身性感染の疑い又は活動性の全身性感染に対して全身治療を受けている患者
② Day 1 前 6 カ月以内に脳卒中（脳血管発作）又は心筋梗塞を発症した患者
③ Day 1 にコントロール不良の高血圧である患者（安静時の収縮期血圧 180 mmHg 超及び／又は拡張期血圧 110 mmHg 超と定義）。スクリーニング期間中の最初の測定でこれらの値を超えた場合、同日又はスクリーニング期間中の別の日に再測定してもよい。
④ スクリーニング前 12 カ月以内の活動性の癌を有する患者。ただし適切に治療された子宮頚部上皮内癌、非黒色腫皮膚癌、グリーソンスコアが 6 以下で前立腺特異抗原が 12 カ月を超えて安定している前立腺癌を除く。
⑤ スクリーニング前 1 カ月以内に何らかの重大な疾患又は重大な外科的処置を経験した患者
⑥ スクリーニング前 1 カ月以内にステロイドの全身投与（例：経口投与又は注射）を受けた患者

⑦ ファリシマブの使用が禁忌となる、研究結果の解釈に影響を及ぼす可能性がある、治療合併症のリスクが高まることが十分に疑われると研究責任医師又は研究分担医師が判断したその他の疾患、代謝機能不全、理学的検査所見又は臨床検査所見の既往又は合併を有する患者
⑧ 妊娠中若しくは授乳中の女性
⑨ 妊娠可能な女性*の場合、研究治療期間中及び研究薬の最終投与日から少なくとも 3 カ月間にわたって、禁欲（異性との性交を行わない）の継続又は 1 年間の失敗率が 1%未満**の許容される避妊法の使用に同意できない患者。
  * 初経後で、閉経後状態（連続12カ月以上にわたり無月経で、閉経以外の原因が認められない状態）に達しておらず、手術（卵巣、卵管及び／又は子宮の摘出）又は研究責任医師又は研究分担医師が判断した他の原因（例：ミュラー管形成不全）により永久的な不妊になっていない女性を妊娠可能とみなす。この規定に従い、片側卵管結紮の女性は妊娠可能とみなす。
  ** 年間避妊失敗率が1%未満の避妊法の例としては、両側卵管結紮術、男性不妊手術、排卵を阻害するホルモン避妊薬、ホルモン放出子宮内器具、銅付加子宮内器具等が挙げられる。性的禁欲の信頼性については、臨床試験の期間及び各患者の好みや通常のライフスタイルに関して評価すべきである。周期的禁欲（カレンダー法、排卵日法、徴候体温法、排卵後法等）及び膣外射精は十分な避妊法ではない。
⑩ 生物学的製剤に高度のアレルギー反応又はアナフィラキシー反応の既往がある患者、又はファリシマブの成分、研究手順に用いる薬剤（フルオレセインを含む）、散瞳点眼薬、研究期間中に使用する麻酔薬及び抗菌薬に対して既知の過敏症がある患者
⑪ Day 1 前 3 カ月以内に薬剤（ビタミン及びミネラルを除く）又は眼科領域の機器を用いて治療を行う臨床試験に参加していた患者
⑫ 以下に示す併用禁止薬剤又は治療を継続的に使用する必要のある患者
  – 抗 VEGF 薬の全身投与
  – 黄斑浮腫の原因となることが知られている全身性の薬剤（フィンゴリモド、タモキシフェン等）
  – 対象眼への抗 VEGF 薬（ファリシマブ以外）の硝子体内投与
  – 対象眼へのステロイドの硝子体内、眼周囲（テノン嚢下）、又は習慣的な眼への局所投与
  – 対象眼へのベルテポルフィン（ビスダイン®）治療
  – 対象眼へのマイクロパルス及び局所又は格子状光凝固術
  – その他の試験的な治療（ビタミン及びミネラルによるものを除く）

※設定根拠：
①～⑩、⑫　安全性の懸念がある又は安全性評価に影響を与えるため
⑪　適切な評価が出来ない又は有効性評価に影響を与えるため

2) 対象眼に適用される除外基準
① スクリーニングの 4 カ月以上前に診断された CRVO 又は HRVO に伴う黄斑浮腫又は CRVO 又は HRVO に伴う持続性黄斑浮腫の既往を有する患者
② 網膜剥離又は黄斑円孔（Stage 3 又は 4）の既往を有する患者
③ 研究責任医師又は研究分担医師により、現在の眼の病態が CRVO 又は HRVO に伴う黄斑浮腫以外の原因で対象眼に不可逆的な視力障害をもたらしつつある又はもたらすことが予想されると判断された患者（例：虚血性黄斑症、アーヴァイン・ガス症候群、中心窩萎縮、中心窩線維症、色素異常、密な中心窩下硬性白斑又はその他の網膜以外の状態）
④ 対象眼の牽引性網膜剥離、全層黄斑円孔、硝子体黄斑牽引症又は中心窩癒着若しくは黄斑構造破壊を伴う網膜上膜を認める患者

⑤ 研究責任医師又は研究分担医師により、中等度の非増殖糖尿病網膜症、より不良の増殖糖尿病網膜症、DME、nAMD 及び地図状萎縮又は近視性脈絡膜新生血管と診断された患者
⑥ 活動性ルベオーシス、隅角新生血管、血管新生緑内障を有する患者
⑦ Day 1 前 3 カ月以内に白内障手術を受けたか、YAG レーザー後嚢切開による白内障手術合併症の治療を受けた患者
⑧ その他の眼内手術（例：経毛様体扁平部硝子体切除術、強膜バックル、緑内障手術、角膜移植、又は放射線療法）を受けた患者
⑨ Day 1 より前に対象眼に対して黄斑レーザー（局所／格子状）又は汎網膜光凝固術を受けたか、Day 1 の治療開始から 3 カ月以内に汎網膜光凝固術を受ける予定がある患者
⑩ 過去に PDT、レーザー、経瞳孔的温熱療法又は sheathotomy 等の網膜硝子体手術による介入を受けた患者
⑪ 黄斑浮腫、糖尿病黄斑浮腫及び新生血管を伴う加齢黄斑変性を含む黄斑部新生血管又は硝子体黄斑界面異常に対する治療として、硝子体内投与（例：抗 VEGF 薬、ステロイド、組織プラスミノゲン活性化因子、C_3F_8、SF_6、空気）、眼周囲注射等の治療を過去に受けたか、現在受けている患者
⑫ 他の網膜疾患に対する眼周囲への薬物療法又は硝子体内注射（抗 VEGF 薬を含む）の治療歴がある患者

※設定根拠：

①～⑫　有効性・安全性の適切な評価が出来ないため

3)　両眼に適用される除外基準

① いずれかの眼に特発性又は免疫介在性ぶどう膜炎の既往のある患者
② Day 1 にいずれかの眼に活動性眼炎症、又は眼及び眼周辺部での感染の疑い又は活動性の感染がある患者

※設定根拠：

①　有効性・安全性の適切な評価が出来ないため

②　安全性の懸念あるいは安全性評価に影響を与えるため

4.2 登録

4.2.1 登録の手順

本研究での患者登録は、電子データ収集（electronic data capture：EDC）を用いた登録とする。

研究責任医師、研究分担医師は、CRB の承認を受けることを必須とする。承認後に支援事務局から登録を行うために EDC システムにログインするためのユーザーID とパスワードを入手し、登録を行う。

研究責任医師又は研究分担医師が、対象者となる患者に対して本研究の説明を行い、患者本人から文書による同意が得られた場合、患者が登録基準をすべて満たし、除外基準に該当しないことを確認した上で、インターネット経由で EDC システムにアクセスする。

登録は、システムの点検やトラブル対処のメンテナンスを除いて、24 時間可能である。EDC システムの指示に従って、必要項目を入力し、患者を登録する。

EDC システム名　：Zelta
URL　：https://zeltatrials.com/ng/login/credential
問合せ先　：zelta.support@merative.com

4.2.2 登録結果の発行と通知

登録画面の入力内容から適格性が確認された症例のみ電子症例報告書（eCRF）の入力を可能とする。

4.2.3　複数研究登録について

他研究の実施計画書を確認し、研究責任医師又は研究分担医師が重複登録することに問題がないと判断した場合は、本研究との重複登録は許容する。ただし、治験との重複登録は不可とする。

4.2.4　登録に際しての注意事項

治療開始後の登録は例外なく許容されない。登録は「4.2.1 登録の手順」の EDC システムの URL へアクセスして行う。適格性の確認は EDC システムの画面上で行われる

登録時に必要とされるデータはすべて必須であり、真正でなければならない。登録後、虚偽登録が判明した場合には重大違反として対処する。

入力データが不十分なときは、すべて満たされるまで登録は受け付けられない。

データの研究利用の拒否を含む全同意撤回があった場合を含み、一度登録された患者の取り消し（データベースからの抹消）はなされない（データの研究利用の拒否を含む全同意撤回があった場合は、データの取り扱いによって解析対象から除くが、申し出の時点で解析報告として固定されている場合は撤回できないものとする）。

誤登録・重複登録が判明した際には、速やかに支援事務局に連絡する。重複登録の場合には、いかなる場合も初回の登録情報（登録番号）を採用する。

4.2.5　登録終了の手続き

本研究で予定した登録数の達成が見込まれる場合、支援事務局は、登録状況を知らせるメールを研究責任医師に配信する。登録終了日について支援事務局は、登録数が達成される旨と、今後の患者登録の注意点（予定登録数に達した以降は、実施医療機関では新規患者への説明は行わない）について実施医療機関に通知する。

4.3　観察・検査項目スケジュール

本研究の観察・検査のスケジュールを Appendix 1 に示す。研究責任医師又は研究分担医師は、研究実施スケジュールに従って観察・検査等を実施する。同日又は 2 営業日以内に全検査を完了して評価する場合は、スクリーニング来院と Day 1 の来院を併せて 1 回の来院として実施してもよい。スクリーニング来院と Day 1 の来院が同じ日に完了する場合は、両方の来院で行う評価は 1 回だけ実施する。

4.3.1　同意取得及びスクリーニング記録

研究責任医師又は研究分担医師は、対象者となる患者が本研究に参加する前に、説明文書を用いて本研究について十分に説明し、対象者となる患者の自由意思に基づく同意を文書により取得しなければならない。対象者となる患者が疾病等何らかの理由により文書による同意を与えることができない場合（視力障害等で文書を読むことはできないが口頭の説明によりその内容を理解することができる場合や、四肢障害等で署名することはできないが文書を読みその内容を理解することができる場合）は、説明及び同意には立会人を立ち合わせた上で行う。立会人は、同意文書に署名と日付を記載し、本研究の対象者となるべき者が本研究を理解し自由意思により同意をしたものであることを証する。なお、立会人は本研究に従事する者であってはならない。登録の有無にかかわらず対象者の同意文書は実施医療機関にて保管し、同意文書の写しを対象者に渡す。

研究責任医師又は研究分担医師は、研究登録前までに対象者となる患者の適格性を確認する。同意取得前であっても、Day 1 前 14 日以内に通常診療の中で得られている検査・評価結果を用いることが可能であり、スクリーニングのために再度検査・評価を行う必要はない。また、同意取得後、研究薬の投与は 28 日以内に実施する。

4.3.2 病歴・手術歴及び患者背景情報

スクリーニング期間に臨床的に重要な疾患の既往歴、手術歴及び薬剤歴（治療開始 7 日前までに服用したすべての医療用医薬品）について、eCRF に記録する。

同意取得後、研究薬の初回投与前に発現した有害事象は、既往歴又は合併症として eCRF に記録する。既往歴には、本剤の治療開始 6 カ月以内に治癒した臨床的に重要な疾患を記録し、合併症には、本剤の治療開始時点で治癒していない疾患を記録する。

患者の背景情報として性別、年齢及び人種を eCRF に記録する。

4.3.3 血圧

研究実施スケジュール（Appendix 1）に従い、座位での収縮期及び拡張期血圧を測定する。

4.3.4 眼科検査

研究実施スケジュール（Appendix 1）に従い、以下の眼科検査を実施する。

4.3.4.1 眼圧測定

眼科検査のための散瞳前に眼圧を測定し、eCRF に記録する。散瞳前の眼圧が 30 mmHg 以上であれば散瞳薬及び研究薬の投与を中止する。持続的な眼圧上昇が認められた場合には、有害事象として eCRF に記録する。

眼圧は、同じ患者では研究期間を通して同じ方法で測定する。

4.3.4.2 屈折検査

対象眼の屈折検査を実施する。屈折検査は散瞳前に実施する。スクリーニング時の球面度数、円柱度数及び円柱軸を eCRF に記録する。臨床的に問題となる屈折異常（近視、遠視、乱視又は不同視）は、合併症として eCRF に記録する。

4.3.4.3 眼軸長検査

眼科検査のための散瞳前に対象眼の眼軸長を測定し、eCRF に記録する。

4.3.4.4 視力検査

視力検査は散瞳前に実施する。ランドルト環視力表を用いて遠距離（5 m）矯正視力を測定し、小数視力を eCRF に記録する。

BCVA は、logMAR で表し、小数視力から以下の式により換算する。

logMAR = log(1/d)　（d = 小数視力）

なお、小数視力 0.02 未満の場合は、指数弁、手動弁又は光覚弁により評価する。

4.3.4.5 眼底検査

<u>FA</u>

別途定める手順書に従い、原則、散瞳下で対象眼の蛍光眼底造影を実施する。画像解析（毛細血管瘤、虚血無灌流領域、血管漏出面積の算出等）は画像解析機関にて実施する。撮影手順、画像データの授受及び画像解析については手順書に記載する。

<u>CFP</u>

別途定める手順書に従い、原則、散瞳下で対象眼のカラー眼底写真を撮影する。画像解析は画像解析機関にて実施する。撮影手順、画像データの授受及び画像解析については手順書に記載する。

4.3.5 光干渉断層撮影（OCT）

SD-OCT 又は SS-OCT

別途定める手順書に従い、原則、散瞳下で対象眼の OCT スキャンを実施し CST を測定するとともに IRF、SRF 及び黄斑上膜の有無を確認し、結果を eCRF に記録する。画像解析（IRF 及び SRF の fluid volume）は画像解析機関にて実施する。実施手順、画像データの授受、画像解析及び解析結果の報告については手順書に記載する。

OCT-A

OCT-A は来院ごとに実施する。原則、散瞳下で対象眼の OCT-A をスキャン範囲 6 mm × 6 mm にて実施し、網膜浅層血管網、網膜深層血管網及び網膜全層血管網における血管密度の測定値を eCRF に記録する。

4.3.6 規定外来院

患者が規定の来院日（±来院の許容範囲）より前に来院することになった場合（規定外来院）、すべての規定来院日に共通して実施する検査（併用薬・併用療法の調査、有害事象、視力検査、眼圧測定、SD-OCT 又は SS-OCT、OCT-A）は必須とし、研究責任医師又は研究分担医師の判断により、その他必要な検査を実施する。

規定外来院で疾患活動性（reference CST に対し CST が 20%以上増加）が確認された場合は、それまでの投与状況に応じて当日の投与の要否及び次回以降の投与間隔を判断する（「5.2 プロトコール治療」参照）。

4.3.7 患者の研究の中止

患者は、いつでも、いかなる理由でも、自らの意思で研究への参加を中止することができる。また、研究責任医師又は研究分担医師は、いつでも患者の研究参加中止を決定することができる。研究中止理由の一部を以下に示す。

- 患者が同意を撤回した場合
- 患者が除外基準に抵触した場合
- 患者が何らかの医学的状態を有しており、安全性の理由により、研究の継続が困難であると研究責任医師又は研究分担医師が判断した場合
- ファリシマブの投与中止又は研究の中止が、患者にとって最も利益になると研究責任医師又は研究分担医師が判断した場合

患者の意思により研究への参加を中止した場合、研究責任医師若しくは研究分担医師又は研究協力者は、各研究対象者の中止日及び中止理由を記録する。また、研究対象者の協力が得られる限り、本研究実施計画書に規定する調査、観察及び検査項目を実施して研究対象者の安全確保に努める。なお、中止時に行う検査は全て実施する必要はなく、研究対象者の意思を確認のうえ実施する。

研究責任医師又は研究分担医師が、有害事象等の発現のため研究の継続が困難と判断した場合は、研究を中止しその後の経過を観察する。なお、研究終了・中止時に疾病等、感染症が未回復の場合は、回復又は軽快するまで可能な限り観察を継続する。

4.3.8 実施医療機関における研究参加中止

研究責任医師が研究を中止又は中断した場合、研究責任医師は実施医療機関の管理者へ速やかにその旨を連絡するとともに、詳細を文書にて説明する。実施医療機関の管理者は研究代表医師に対し、速やかにその旨を文書で連絡するとともに、詳細を文書にて説明する。

研究代表医師は、CRB の継続審査等において、医療機関の研究参加の中止について報告し、厚生労働省が整備するデータベース（jRCT*）の登録情報の変更を行う。

* 臨床研究法施行規則第 24 条第 1 項に規定する厚生労働省が整備するデータベース（Japan Registry of Clinical Trials: 臨床研究等提出・公開システム）

URL: https://jrct.niph.go.jp/

5. 治療計画と治療変更基準

5.1 研究薬

一般名	ファリシマブ（遺伝子組換え）
販売名	バビースモ硝子体内注射液120 mg/mL
投与経路	硝子体内投与
添付文書に記載の用法・用量[19]<br>（網膜静脈閉塞症に伴う黄斑浮腫）	ファリシマブ（遺伝子組換え）として1回あたり6.0 mg（0.05 mL）を硝子体内投与する。投与間隔は、4週以上あけること。
製造販売元	中外製薬株式会社

5.2 プロトコール治療

本研究に参加する患者はファリシマブ 6.0 mg の硝子体内投与を受ける。参加期間は 72 週間で、全患者を対象に Day 1 にてファリシマブを投与し、以降、導入期、観察期、及び維持期の各基準に従って投与を行う。来院間隔については、Day 1 から W24 までは 4 週ごと、W24 以降は W36（観察期の患者のみ）、W52、W72 を規定来院とした上で、mTAE レジメンに従って決定した投与間隔に応じた頻度で来院する。

なお、本研究では、「黄斑浮腫の消失」、「疾患活動性」、及び「reference CST」を以下のとおり定義する。

黄斑浮腫の消失	Spectralis スペクトルドメイン型光干渉断層撮影（SD-OCT）で測定した CST が325 µm 未満、又は Cirrus SD-OCT、Topcon SD-OCT 若しくはその他これらに準じる OCT で測定した CST が315 µm 未満（これを基準として研究責任医師又は研究分担医師が判断する）
疾患活動性	reference CST に対し CST が20%以上増加
reference CST	ファリシマブ投与開始後、最初に黄斑浮腫の消失を認めたときの CST 値、又はファリシマブの6回連続投与で黄斑浮腫の消失を認めなかった場合はそれまでの CST の最低値

1) 導入期

ファリシマブ投与開始から最初に黄斑浮腫の消失が確認されるまでの期間（黄斑浮腫の消失が確認されない場合は W20 までの期間）を導入期とする。

全患者を対象に Day 1 で 1 回ファリシマブを投与し、以降は 4 週ごとに 1 回投与する。W4 以降、投与前に黄斑浮腫の消失が確認された場合、ファリシマブを投与せず、観察期に移行する。なお、当該来院日の CST 値を、reference CST とする。

導入期のファリシマブ投与回数は最大 6 回までとする。W20 まで 6 回連続投与しても黄斑浮腫の消失が確認できない場合は、次回投与日を W24 として観察期を経ずに維持期に移行する。この場合、Day 1 から W20 までに得られた CST の最低値を reference CST とする。

なお、黄斑浮腫の消失は、本研究実施計画書で定める基準に基づいて研究責任医師又は研究分担医師が判断するが、基準を満たしていない場合であっても、判断理由を記録した上で黄斑浮腫の消失とみなすことができる。

2) 観察期

黄斑浮腫の消失が確認された来院日から再び疾患活動性が確認される日までを観察期とする。

観察期には、ファリシマブを投与せず、W24 までは 4 週ごとに、W24 以降は疾患活動性が認められない限り W36、W52 及び W72 の規定来院日にモニタリングを行う。来院時に疾患活動性が確認された場合は、同日又は当該来院の許容範囲（規定来院日の-7 日～+14 日）内にファリシマブを投与し、維持期に移行する。

疾患活動性の基準を満たしていない場合であっても、前回の来院と比し大幅な視力低下（例：小数視力 0.6→0.3）を伴い、かつ、治療が必要な浮腫があると研究責任医師又は研究分担医師が判断した場合は「疾患活動性あり」と判断し、ファリシマブの投与を許容する。また、疾患活動性の基準を満たしている場合でも、自覚症状を認めず臨床的に意味のある変動ではないと研究責任医師又は研究分担医師が判断した場合は「疾患活動性なし」と判断することを許容する。研究責任医師又は研究分担医師はこれらの判断理由を記録する。

また、研究責任医師又は研究分担医師が必要と判断した場合は、規定来院の間でも規定外来院にてモニタリングを行う。規定外来院時に疾患活動性が確認された場合はファリシマブを投与し、維持期に移行する。

3) 維持期

観察期の期間と同じ期間（観察期を経ず維持期に移行した場合は 4 週）を、維持期における最初の投与間隔と設定する。維持期の投与間隔は以下の調整基準に沿って 4 週ごとに調整する。投与間隔の調整幅は±4 週であり、最短の投与間隔は 4 週とするが、最長については設定しない。この調整基準とは異なる調整が必要であると研究責任医師又は研究分担医師が判断した場合は、判断理由を記録した上で投与間隔を調整する。

Reference CST からの CST の変化率（%）	投与間隔の調整
+10%未満	延長（+4週）
+10%以上+ 20%未満	維持（±0週）
+20%以上	短縮（-4週）

疾患活動性の基準を満たしていない場合であっても、前回の来院と比し大幅な視力低下（例：小数視力 0.6→0.3）を伴い、かつ、治療が必要な浮腫があると研究責任医師又は研究分担医師が判断した場合は「疾患活動性あり」と判断し、投与間隔を短縮する。また、疾患活動性の基準を満たしている場合でも、自覚症状を認めず臨床的に意味のある変動ではないと研究責任医師又は研究分担医師が判断した場合は、判断理由を記録した上で「疾患活動性なし」とし投与間隔を延長又は維持できる。

ファリシマブの硝子体内投与にあたっては、必ず最新の添付文書[19]及び適正使用ガイド[33]を確認し、適正に使用する。

5.3 プロトコール治療中断／中止基準

以下のいずれかの基準に該当する場合、研究薬による治療を中断又は中止する。治療の中断／中止理由を該当する eCRF のページに記録し、有害事象発現に伴う中断／中止の場合は eCRF の有害事象のページに記録する。

表 5.3-1　　投与中断及び治療中止の基準

事象	基準
眼内炎症	• 対象眼の眼内炎症（虹彩炎、虹彩毛様体炎、硝子体炎等）が2+以上の場合は、研究薬の投与を中断する。<br>• 研究責任医師又は研究分担医師の判断により、後に投与を再開してもよい。
対象眼の白内障手術	• 対象眼の白内障手術後には研究薬の投与を中断する。<br>• 合併症のない白内障手術から28日以降には、その時点で術後の炎症が認められない場合、研究薬の投与を再開してもよい。白内障手術の合併症を認める場合は、研究責任医師又は研究分担医師の判断により研究薬の投与が許可される場合がある。
BCVA 低下	• 対象眼で直近の治療前最後の BCVA 評価と比較して、研究薬の投与に関連する logMAR で0.6以上の BCVA 低下が認められる場合は、研究薬の投与を中断する。<br>• 研究責任医師又は研究分担医師の判断により、その後研究薬の投与が許可される場合がある。
眼圧上昇	• 対象眼の投与前の眼圧が30 mmHg 以上の場合は、研究薬の投与を中断する。<br>• 自然に又は治療により眼圧が30 mmHg 未満に低下した場合は、研究責任医師又は研究分担医師の判断により、研究薬の投与が許可される場合がある。
裂孔原性網膜裂孔	• 対象眼に網膜裂孔が認められる場合は、研究薬の投与を中断する。<br>• レーザーによる網膜復位に成功した場合、研究責任医師又は研究分担医師の判断により、術後28日以降に研究薬の投与を再開してもよい。
裂孔原性網膜剥離又は黄斑円孔	• 対象眼に裂孔原性網膜剥離又は Stage 3又は4の黄斑円孔が発現した場合は、研究薬の投与を中断する。<br>• 研究責任医師又は研究分担医師の判断により、研究薬の投与が許可される場合がある。
活動性の感染又は感染の疑い	• 眼及び眼周辺での活動性の感染又は感染の疑い（例：感染性結膜炎、感染性角膜炎、感染性強膜炎若しくは眼内炎）が認められるか、又は活動性の全身性感染症に対して治療が必要な場合は、研究薬の投与を中断する。<br>• 研究責任医師又は研究分担医師の判断により、研究薬の投与が許可される場合がある。
併用禁止薬剤又は療法の使用	• 「5.4.4 併用療法・支持療法」に記載の併用禁止薬・併用禁止療法を使用した場合、研究を中止する。

何らかの理由で投与を中断した場合に、投与を再開する場合は、以下の手順に従う。

- 再開時に投与を行い、中断前時点での投与間隔に応じてその後の投与スケジュールを決定する。

5.4 併用療法・支持療法

5.4.1 規定とする併用療法・支持療法

該当なし。

5.4.2　推奨される／推奨されない併用療法・支持療法

該当なし。

5.4.3　許容される併用療法・支持療法

本研究において、ファリシマブとの併用が許容される一般的な治療法の一部を以下に示す。

- 研究参加中に対象眼に発症した高眼圧症又は緑内障は、臨床的に必要であれば治療する。
- 研究参加中にいずれかの眼に発症した白内障又は後嚢混濁は、臨床的に必要であれば治療してもよい。白内障手術には投与中断基準（「5.3 プロトコール治療中断／中止基準」、表 5.3-1 参照）が適用される。
- 白内障手術、YAG レーザー後嚢切開術、周辺虹彩切開術、アルゴン／選択的レーザー線維柱帯形成術、又は眼アレルギーに続く眼に対する局所ステロイドの短期使用
- 対象眼に対する完全、部分又は局所汎網膜光凝固術は、虚血性の RVO 又は周辺部の新たな新生血管の治療のために必要と研究責任医師又は研究分担医師が判断した場合は実施可能。実施した場合は、併用療法として記録し、実施理由も記録する。
- 対象眼に視力を脅かす硝子体出血又は網膜剥離が生じた場合は硝子体切除術を実施してもよい。これらの病態は、有害事象として記録し、併用療法としても記録する。治療を中断し、患者の状態に基づいて再開してもよい。患者は、来院を計画通り完了すること。
- 非対象眼への抗 VEGF 薬投与（研究責任医師又は研究分担医師の判断で、非対象眼に対する使用が認可された抗 VEGF 薬を投与してもよい。ただし、初回治療における両眼同日投与は避け、片眼での安全性を十分に評価した上で対側眼の治療を行うこと。）

5.4.4　併用禁止薬剤及び療法

患者の研究参加中、以下の薬剤及び治療の使用は禁止される。使用する際は、研究を中止する。

- 抗 VEGF 薬の全身投与
- 黄斑浮腫を引き起こすことが知られている全身性薬物（フィンゴリモド、タモキシフェン等）
- 対象眼への抗 VEGF 薬（ファリシマブ以外）の硝子体内投与
- 対象眼へのステロイドの硝子体内、眼周囲（テノン嚢下）、眼内インプラント、又は習慣的な眼への局所投与
- 対象眼へのベルテポルフィン（ビスダイン®）治療
- 対象眼へのマイクロパルス及び局所又は格子状光凝固術
- その他の試験的な治療（ビタミン及びミネラルによるものを除く）

5.5　後治療

後治療については規定を設けない。

6. 安全性評価

6.1　個々の薬剤で予期される薬物有害事象

本研究に用いる個々の薬剤の詳細については、最新の添付文書を参照すること。（参考：独立行政法人医薬品医療機器総合機構 https://www.pmda.go.jp/PmdaSearch/iyakuSearch/）

6.2　有害事象の定義

6.2.1　有害事象の定義

有害事象とは実施された研究との因果関係の有無を問わず、患者に生じたすべての好ましくない意図しない傷病若しくはその徴候（臨床検査値の異常を含む）と定義する。本研究ではファリ

シマブの治療開始以降、最後の研究来院日までに発現したすべての有害事象を評価対象とする。有害事象は、消失、軽快又は安定するまで転帰を追跡する。

6.2.2　疾病等の定義

有害事象のうち、本研究の実施に起因するものと疑われる疾病、障害若しくは死亡又は感染症に加え、臨床検査値の異常や諸症状を疾病等とする。本研究においては、有害事象が研究薬に関連しているという合理的な可能性がある場合、研究実施計画書で定める検査の実施に関連しているという合理的な可能性がある場合を指す。

「感染症」とは、生物由来製品において、生物由来の原料又は材料から、当該医薬品等への病原体の混入が疑われる場合等をいう。また、B 型肝炎ウイルス（HBV）、C 型肝炎ウイルス（HCV）、ヒト免疫不全ウイルス（HIV）等のウイルスマーカーの陽性化についても、感染症報告の対象となる。

6.2.3　有害事象の程度

有害事象の重篤度は 0.非重篤、1.重篤の 2 段階で有害事象ごとに判定する。

なお、本研究では、医薬品、医療機器等の品質、有効性及び安全性の確保等に関する法律に準じて、下記に該当するものを重篤な有害事象として扱う。

【重篤な有害事象の定義】

1)　死に至るもの
2)　生命を脅かすもの
3)　治療のための入院又は入院期間の延長が必要となるもの
4)　永続的又は顕著な障害・機能不全に陥るもの
5)　先天異常・先天性欠損を来すもの
6)　その他の医学的に重要な状態と判断される事象又は反応

6.2.4　有害事象の重症度判定

有害事象の重症度の判定基準を以下に示す。

表 6.2-1　有害事象重症度分類スケール

重症度	定義
軽度	不快感の自覚はあるが、通常の日常活動に支障はない。
中等度	日常活動量を減少させる、又は日常活動に影響を及ぼす程度の不快感がある。
高度	働くことができない、又は通常の日常活動を行うことができなくなるような障害。

※重症度に関わらず、重篤度の基準に適合する場合もある。重篤な有害事象については「6.2.3 有害事象の程度」を参照。

6.2.5　因果性分類判定基準

有害事象の因果性判定の際の基準は以下のとおりとする。

表 6.2-2　有害事象と治療との因果関係の判定基準

因果性分類	判定基準
あり	有害事象がファリシマブに関連しているという合理的な可能性がある場合<br>以下のいずれかに該当する場合。<br>• 投与再開による事象再発<br>• 薬理作用から十分予測可能である／同じ薬効群の薬も含め因果関係が確立されている<br>• ファリシマブ投与と発現した事象とが時間的に説明できる<br>• ファリシマブのみ投与中止による事象消失<br>• 原疾患、合併症、既往症、併用治療等では説明できない
なし	有害事象とファリシマブの因果関係を否定できる場合<br>以下のいずれかに該当する場合。<br>• ファリシマブとの時間的関連性がみられない<br>• 他の要因（原疾患、合併症、既往症、併用治療等）で説明するのが妥当である

7.　有害事象又は健康被害の恐れのある機器の不具合の報告

7.1　有害事象又は健康被害の恐れのある機器の不具合の報告期間

本研究におけるファリシマブの初回投与開始後は、研究薬との関連の有無を問わず、最終来院日、同意を撤回した日又は追跡不能になった日までに発生したすべての有害事象又は健康被害の恐れのある機器の不具合が報告対象となり、eCRF に記録する。

7.2　すべての有害事象又は健康被害の恐れのある機器の不具合発生時の対応

研究責任医師又は研究分担医師は、患者と接するときはその都度有害事象に関する情報の収集に努め、eCRF の情報更新を行う。有害事象又は健康被害の恐れのある機器の不具合は、患者により報告されたもの、又は実施医療機関の研究関係者によって発見されたものを区別せず、すべて患者の診療録に記録する。

支援事務局は、実施医療機関より有害事象又は健康被害の恐れのある機器の不具合に関する情報を知り得た場合、予め定めた手順に従って中外製薬株式会社へ報告する。

中外製薬株式会社は入手した有害事象又は健康被害の恐れのある機器の不具合情報の評価を行い、必要に応じて独立行政法人医薬品医療機器総合機構及び安全性情報授受の契約を結んだ関係会社に有害事象又は健康被害の恐れのある機器の不具合情報の報告を行う。また、必要に応じて実施医療機関への周知事項の連絡等の対策を講じる。また、研究責任医師又は研究分担医師は、可能な限り中外製薬株式会社からの照会事項に関する追加調査へ協力する。

研究代表医師は、実施医療機関より入手した有害事象又は健康被害の恐れのある機器の不具合に対して、報告内容の緊急性、重要性、影響を考慮の上、必要に応じて研究実行委員に意見を求めて対応を判断する。

7.3　疾病等、感染症への対応

研究代表医師及び研究責任医師は、疾病等、感染症の情報を入手した場合、以下の 1)～6)の手順で報告を行う。

情報を入手した者が研究分担医師の場合は、速やかに研究責任医師に伝え、同様の対応を取るが、研究責任医師に連絡が取れない場合は、研究分担医師が、研究責任医師の責務を代行しなければならない。

1) 研究責任医師は、有害事象のうち重篤な疾病等、重篤な感染症、非重篤の未知の感染症に関する情報を入手した場合は、速やかに実施医療機関の管理者に報告した上で、10 日以内に「統一書式 8 医薬品疾病等報告書」（以下、「書式 8」）に所定事項を可能な範囲で記入し、研究代表医師、研究事務局、支援事務局の安全性管理業務担当者に電子メールにて連絡する。第一報報告後、新たな情報が得られた場合は、「書式 8」に情報を追記し、随時研究代表医師／研究事務局／支援事務局に報告する。
2) 研究代表医師／研究事務局／支援事務局は、「書式 8」を入手後、以下に定める報告期限に従い、研究代表医師の所属する実施医療機関の管理責任者及び CRB へ「書式 8」を提出する。ただし、未知の死亡に関する情報を入手した場合等、緊急を要する場合、速やかに研究代表医師の所属する施設の管理責任者へ報告する。報告方法は電話、口頭を問わない。

	感染症以外		感染症	
	未知*	既知**	未知*	既知**
死亡	15日	15日	15日	15日
重篤	15日	30日	15日	15日
非重篤	定期報告	定期報告	15日	定期報告

* 研究責任医師又は研究分担医師は、発生した疾病等が次に掲げるいずれの文書にも記載されておらず、予測することができない場合は「未知」のものとして扱う：①研究実施計画書又は説明文書・同意文書、②本研究に用いる医薬品等の添付文書の重大な副作用及びその他の副作用、③本研究に用いる医薬品等の適正使用ガイド [33]。

** 発生した疾病等が上記①、②又は③の文書に記載されている場合は、既知のものとして扱う。

3) 研究代表医師／研究事務局／支援事務局は、2)の研究事務局に報告後、すべての実施医療機関の研究責任医師へ、CRB に報告を行ったことを報告するとともに、当該情報を提供する。また、提出した「書式 8」の写しを CRB 報告後に遅延なく共同研究機関に提供する。

4) すべての実施医療機関の研究責任医師は、当該情報提供の内容を自らが所属する実施医療機関の管理者に報告を行う。
5) 研究代表医師／研究事務局／支援事務局は、CRB から当該情報についての審査結果を入手した場合、実施医療機関の管理者に報告を行うとともに、すべての実施医療機関の研究責任医師へ審査結果を報告する。
6) すべての実施医療機関の研究責任医師は、自らが所属する実施医療機関の管理者へ CRB からの審査結果の報告を行う。

※「書式8」は、厚生労働省ウェブサイトにて最新版を入手できるため、報告に際しては最新版を用いること。（厚生労働省ウェブサイト：http://www.mhlw.go.jp/stf/seisakunitsuite/bunya/0000163417.html）

CRB は、疾病等報告を受けた場合において、必要があると認めるときは、研究責任医師又は研究代表医師に対し、当該報告に係る疾病等の原因の究明又は再発防止のために講ずべき措置について意見を述べなければならない。研究代表医師又は研究責任医師は、当該疾病等について報告を受けた CRB が研究代表医師又は研究責任医師に対して意見を述べたときは、当該意見を尊重して必要な措置をとらなければならない。

7.4 有害事象発現後の患者フォローアップ

研究責任医師又は研究分担医師は本研究におけるファリシマブ投与開始後、有害事象（臨床検査値に関する有害事象を含む）が認められた場合は、最善の処置・治療を行う。有害事象等の発現のため、研究の継続が困難と判断した場合は、研究を中止し、その後の経過を観察する。

なお、研究終了・中止時に疾病等、感染症が未回復の場合は、回復又は軽快するまで可能な限り観察を継続する。

各研究対象者の研究中に転帰の変更が生じた場合は、その旨を日付とともに、原資料及び eCRF に記録する。

7.5 女性患者の妊娠

研究中又はファリシマブの最終投与後 3 カ月以内に女性患者に妊娠が確認された場合、直ちに研究責任医師又は研究分担医師に報告するよう同意文書を通じて指示する。研究責任医師又は研究分担医師は、妊娠の事実を知りえた後「妊娠症例に関する調査報告書」（別紙 3）を介して研究事務局に報告し適切に対応する。なお、女性患者の妊娠は eCRF に記録しない。

また、出生児の健康状態を「妊娠症例に関する調査報告書」（別紙 3）に記録し、研究事務局に提出する。これに加え、出生後 6 カ月時点、12 カ月時点での出生児の健康状態に関する情報を「出生児に関する調査報告書」（別紙 4）に記録し、研究事務局／支援事務局に提出する。

7.6 過量投与／投薬過誤／薬物乱用／薬物誤用に関する報告

研究責任医師又は研究分担医師はファリシマブの過量投与若しくは投薬過誤にあたる事象が確認された場合は、「過量投与／投薬過誤／薬物乱用／薬物誤用に関する調査報告書」（別紙 2）を介して研究事務局／支援事務局に報告する。

8. 統計学的考察及び解析計画

統計解析の詳細はデータ固定前までに別途作成する SAP に規定する。SAP の内容に変更が発生する場合には、必要に応じて、「10.8 変更申請時の手続き」に沿って改訂する。

8.1 予定登録数・予定総研究期間

「3.5 目標症例数の設定根拠」で示した根拠に基づき、72 例と設定した。

予定登録期間・予定追跡期間は、CRB への手続きに係る期間等を見込んで以下のように設定し

た。
- 予定登録数　　：72 例
- 予定登録期間　：最初の症例登録日から 1 年 6 カ月
- 予定追跡期間　：最後の症例登録日から 72 週間
- 報告書作成期間：追跡期間終了から 1 年
- 予定研究期間　：jRCT の公表日から 4 年

8.2 解析対象集団

解析対象集団は以下のように定義する。

- 最大の解析対象集団（FAS）：少なくとも1回ファリシマブの投与を受け、投与後の有効性データを有する全例
- Per-protocol 集団（PPS）：FAS のうち、重大な研究実施計画書違反のない全例
- 安全性解析対象集団：少なくとも1回ファリシマブの投与を受けた全例

8.3 主要評価項目の解析

本研究の有効性の主要評価項目は、W72 における BCVA のベースラインからの変化量である。

COMINO 試験で得られた W72 における BCVA のベースラインからの変化量との一貫性の評価を探索的に行う。一貫性評価において、統計的仮説は設定されず、検定は実施しない。

BCVA はランドルト環を用いて 5 m の距離で測定した小数視力を logMAR に換算した値とする。

logMAR = log(1/d)　（d = 小数視力）

なお、小数視力 0.02 未満の場合は、指数弁、手動弁又は光覚弁により評価し、以下の表により小数視力及び logMAR に換算する。

	小数視力	logMAR
指数弁（50 cm／指数弁）	0.01	2.0
手動弁	0.005	2.3
光覚弁	0.002	2.7
光覚なし	0.001	3.0

BCVA は、MMRM を用いて解析する。モデルには、固定効果として来院（カテゴリー変数）及びベースラインの BCVA（連続変数）を含め、患者内誤差のモデリングには無構造の共分散構造を仮定する。モデルの収束に問題がある場合は、Compound Symmetry 又は AutoRegressive(1) 共分散構造を用いる。欠測のメカニズムはランダムな欠測を仮定する。データの研究利用の拒否を含む全同意撤回症例については、該当症例を評価対象から除外する。

また、副解析として、logMAR から以下の式[34]を用いて換算した ETDRS 文字数の近似値についても、主要評価項目と同様の解析を行う。

ETDRS 文字数 = 85 – 50 x logMAR = 85 – 50 x log(1/d)　（d = 小数視力）

なお、小数視力 0.02 未満（指数弁、手動弁又は光覚弁による評価）の場合、ETDRS 文字数は 0 文字として計算する。

8.4　副次的評価項目の解析

連続変数については、平均値、中央値、範囲、分位範囲及び SD を算出する。
カテゴリー変数は絶対度数及び相対度数とする。
部分集団（SAP に規定）については主要解析を反復する。
副次的評価項目の解析計画の詳細は、SAP に記載する。

8.5　探索的評価項目の解析

連続変数については、平均値、中央値、範囲、分位範囲及び SD を算出する。
カテゴリー変数は絶対度数及び相対度数とする。
各パラメータ同士の散布図等を作成する。
探索的評価項目の解析計画の詳細は、SAP に記載する。

8.6　安全性評価項目の解析

例数と割合を集計する。
安全性評価項目の解析計画の詳細は、SAP に記載する。

8.7　中間解析

全例で投与開始後24週時点における BCVA のデータが得られた時点で、主要解析を含む一部の解析を実施する。

8.8　研究の終了

研究代表医師は、主要評価項目報告書並びに総括報告書及びその概要を作成し、CRB の意見を聴いた上で、研究が終了したことを本研究の関係者に報告するとともに、研究代表医師が所属する医療機関の管理者に対して、その旨及び結果の概要を文書で報告する。各概要は厚生労働大臣に提出し、厚生労働省が整備する jRCT にて公表する。

本研究は、総括報告書の概要を jRCT に登録し、公表した日をもって終了日とする。

9.　データの収集と管理

9.1　データの品質保証

支援事務局のデータマネジメント担当者は、データの品質確認を含む本研究のデータマネジメントの責任を負う。入力されるデータは、EDC を介して eCRF にて収集される。EDC へのデータ入力は実施医療機関の責任において行う。データの矛盾が確認された場合は、データマネジメント担当者は実施医療機関に対し正確なデータの入力を依頼し、実施医療機関は EDC 上で電子的にデータの矛盾を解消する。

eCRF とその修正履歴は、EDC に監査証跡として保持される。支援事務局が保存するデータのシステムバックアップと研究データの保管については同機関の標準手順に従う。

9.2　電子症例報告書（eCRF）

登録された全例の eCRF を作成及び提出する。

eCRF への入力は「4.2 登録」で使用する EDC システムを用いて行う。実施医療機関の研究責任医師又は研究分担医師、研究協力者に対して eCRF へのアカウントが発行される。eCRF は電子的にデータベースに送信、保存され、別途定める手順書に従って取り扱われる。

eCRF への入力は、アカウントが発行された実施医療機関の担当者が行う。研究責任医師は、eCRF の内容を確認し、電子的に署名する。

9.2.1 eCRF の項目

Appendix 2 参照

9.3 原データの特定

9.3.1 eCRF のみに入力されているデータ

eCRF のみに入力されている以下の項目については、eCRF を原データとする。

1) 投与状況：投与中断／中止理由、投与スケジュールの変更理由
2) 有害事象：有害事象の重症度、重篤度、因果関係の判定、転帰及び転帰日
3) 併用薬、併用療法：併用薬、併用療法の目的
4) 研究の中止：中止理由

9.3.2 原資料

原資料とは、患者データが記録された資料（紙又は電子資料）を指す。入院・通院記録、診療録、臨床検査記録、メモ、薬剤処方記録、自動測定機器からの記録データ、検証により正確性と完全性が確認された複写の写し、マイクロフィッシュ、写真ネガ、マイクロフィルム又は磁気メディア、X 線写真、患者ファイル、及び臨床研究に係る薬剤部、臨床検査室又は医療技術部門に保管されている各種記録等が含まれるが、これらに限定されるものではない。

eCRF に入力されたデータの妥当性と完全性を検証するために必要となる原資料は、削除及び破棄を禁止とし、記録保管に関する方針に従って保管しなければならない。

研究責任（代表）医師及び実施医療機関は、本研究に関連するモニタリング（監査）並びに CRB 及び規制当局の調査の際に、原資料等の全ての臨床関連記録を直接閲覧に供することとする。

9.3.3 コンピューター化システムの使用

臨床所見を実施医療機関の電子カルテシステムに原本であるハードコピー記録の代わりとして直接入力する場合は、その電子記録を原資料として扱うことができる。

用いられるコンピューター化データ収集システムでは、最初に入力されたデータ（元データ）が保存されている必要がある。すなわち、元データが変更された場合も、システム上には元データとその変更理由、変更者、変更日時の確認が可能な監査証跡が記録されていなければならない。

9.4 記録・情報・試料の取扱い及び保存

9.4.1 実施医療機関

患者の同意に関する記録、報告書（eCRF）作成のための基礎データ（検査データ等）、CRB の承認書、医療機関において作成された記録文書については、研究責任医師又は研究分担医師が実施医療機関の標準業務手順書に従い適切に保管する。保管期間は、研究全体の中止若しくは終了後 5 年を経過した日、又は各研究実施医療機関に定める期間を経過した日のいずれか遅い日までとする。保存期間終了後、実施医療機関の規定に従い適切に廃棄する。

9.4.2 共同研究機関

共同研究機関は、中外製薬株式会社が作成・管理する、臨床研究に関する記録の保存、情報の保管及び廃棄に関する「標準業務手順書」に従い、共同研究機関が保存すべき研究に係る文書又は記録を保存する。保管期間は、研究全体の中止又は終了後 5 年を経過した日までとする。保存期間終了後、中外製薬株式会社が作成・管理する「標準業務手順書」に従い適切に廃棄する。

9.4.3 支援事務局

本研究中に eCRF に入力された情報は EDC システムを管理する支援事務局のサーバー上に保管される。EDC システムに入力されたデータは固定後、解析が行われる。

支援事務局は､本研究に係る文書又は記録を､支援事務局の標準手順書に従い適切に保管する。保管期間は、研究全体の中止又は終了後 5 年を経過した日までとする。

9.4.4　　画像解析機関

本研究に係る文書又は記録を､画像解析機関の標準手順書に従い適切に保管する。保管期間は、研究全体の中止又は終了後 5 年を経過した日までとする。

9.5　情報提供の記録の保存及び廃棄

本研究においては本研究組織以外の他の研究機関への情報の提供は行わないが、研究に関する業務の委託として、支援事務局が情報を収集する。このため、トレーサビリティの観点から、支援事務局が情報提供に関する以下の記録を保存する。これらの記録は､「9.4.3 支援事務局」の記載に従って廃棄する。

提供先の機関名	「15 研究実施体制 臨床研究業務受託機関」参照
提供先の機関の研究責任医師名	「15 研究実施体制 臨床研究業務受託機関」参照
提供元の名称	「別紙 1：実施医療機関及び研究責任医師一覧」参照
提供元の研究責任医師名	「別紙 1：実施医療機関及び研究責任医師一覧」参照
情報の項目	「9.2.1 eCRF の項目」参照
情報の取得経緯	実施医療機関にて研究実施計画書に従い取得した。
患者の氏名等	患者の氏名等は｢同意書｣を適切に実施医療機関で保管することで代用する。

10. 倫理的事項

10.1　患者の保護

本研究に関係するすべての研究者は「ヘルシンキ宣言」（日本医師会訳）*､「臨床研究法（平成 29 年法律第 16 号）」**､「臨床研究法施行規則（平成 30 年厚生労働省第 17 号）」**、及び関連通知並びに本研究実施計画書に従って研究を実施する。

*　https://dl.med.or.jp/dl-med/wma/helsinki2013j.pdf

**　https://www.mhlw.go.jp/stf/seisakunitsuite/bunya/0000163417.html

10.2　インフォームドコンセント

10.2.1　　説明と同意

研究責任医師又は研究分担医師は、対象者となる患者が本研究に参加する前に、説明文書を用いて以下の事項について説明を行う。対象者となる患者には質問や相談に対応する機会や十分に考える時間を確保し、対象者となる患者が研究内容をよく理解したことを確認した上で研究への参加を依頼する。対象者となる患者が研究参加に同意した場合、同意書に患者本人による署名を得る。登録の有無にかかわらず患者の同意文書は実施医療機関にて保管し、同意文書の写しを患者に渡す。視力障害等で文書を読むことはできないが口頭の説明によりその内容を理解することができる者や、四肢障害等で署名することはできないが文書を読みその内容を理解することができる者に対する説明及び同意には、立会人を立ち合わせた上で行う。その場合、立会人は、同意文書に署名と日付を記載し、本研究の対象者となるべき者が本研究を理解し自由意思により同意をしたものであることを証する。なお、立会人は本研究に従事する者であってはならない。

1) 実施する特定臨床研究の名称及び当該特定臨床研究の実施について実施医療機関の管理者の承認を受けている旨及び厚生労働大臣に実施計画を提出している旨

2) 実施医療機関の名称及び研究責任医師の氏名、職名及び連絡先(特定臨床研究を多施設共同研究として実施する場合にあっては、研究代表医師の氏名及び職名並びに他の実施医療機関の名称並びに当該実施医療機関の研究責任医師の氏名及び職名を含む。)
3) 特定臨床研究の対象者として選定された理由
4) 特定臨床研究の実施により予期される利益及び不利益
5) 特定臨床研究への参加を拒否することは任意である旨
6) 同意の撤回に関する事項
7) 特定臨床研究への参加を拒否すること又は同意を撤回することにより不利益な取扱いを受けない旨
8) 特定臨床研究の情報公開の方法
9) 特定臨床研究の対象者の求めに応じて、研究実施計画書その他当該特定臨床研究の実施に関する資料を入手又は閲覧できる旨及びその入手又は閲覧の方法
10) 特定臨床研究の対象者の個人情報の保護に関する事項
11) 試料等の保管及び廃棄の方法
12) 研究の資金源等、研究機関の研究に係る利益相反及び個人の収益等、研究者等の研究に係る利益相反に関する状況
13) 苦情及び問合せへの対応に関する体制
14) 特定臨床研究の実施に係る費用に関する事項
15) 他の治療法の有無及び内容並びに他の治療法により予期される利益及び不利益との比較
16) 特定臨床研究の実施による健康被害に対する補償及び医療の提供に関する事項
17) 特定臨床研究の審査意見業務を行う CRB における審査事項その他当該特定臨床研究に係る CRB に関する事項
18) その他特定臨床研究の実施に関し必要な事項（研究の目的、意義、方法、期間、データの二次利用について等）

10.2.2 同意撤回

同意撤回とは、研究参加への同意の撤回を意味し、治療継続の拒否（下記 1)）とは区別する。同意の撤回が表明された場合には、下記 2)又は 3)のいずれであるかを明確にし、eCRF の「中止／終了報告」に入力する。

同意撤回（下記 2)）の場合は、以降の実施計画書に従ったフォローアップの依頼を中止する。全同意撤回（下記 3)）の場合は、全同意撤回であることが確認された時点で、当該患者のデータをデータ閲覧、修正、出力、及び解析ができないようにする。

当該患者のフォローアップの依頼の中止及び患者データの解析対象からの削除の手順は別途、定めることとする。

1) 治療継続の拒否：以降の治療継続の拒否（フォローアップは継続する）。
2) 同意撤回：研究参加への同意を撤回し、以降の研究実施計画書に従った治療、フォローアップのすべてを不可とすること。同意撤回以前のデータの研究利用は可とする。
3) 全同意撤回：研究参加への同意を撤回し、登録時の情報を含む研究参加時点からのすべてのデータの研究利用を不可とすること。ただし、すでに解析報告として固定されている場合は撤回できないものとする。

10.3 患者から相談等への対応

研究責任医師又は研究分担医師は、患者及びその関係者からの本研究に係る相談窓口を設置し、連絡方法を説明文書に記載する。登録後に患者やその家族から本研究に関する相談があった場合には、原則として当該患者の実施医療機関の研究者（研究責任医師又は研究分担医師）が対応にあたる。対応の方法が不明な場合には、必要に応じて支援事務局を通じて研究代表医師及び研究実行委員と協議の上で対応する。

10.4　個人情報の保護と患者の識別

患者の氏名は実施医療機関からモニタリング担当者を除く支援事務局の担当者へ知らされることはない。

患者の同定や照会は、登録時に発行される登録番号、患者識別コードを用いて行われる。患者名等、第三者が当該施設の職員やデータベースへの不正アクセスを介さずに直接患者を識別できる情報が、本研究のデータベースに登録されることはない。なお、本研究結果が公表される際には、患者の個人を特定する情報は一切公表されない。

実施医療機関での個人情報の利用にあたっては情報流出のリスクを最小化すべく、患者情報の機密保護に十分配慮し、実施医療機関における規定に従って適切に管理する。

10.4.1　個人情報の利用目的と利用する項目及び利用方法

1) 利用目的

「最善の治療法をより多くの患者へ提供すること」に従い、「臨床研究の正しい結果を得るために、治療中だけでなく治療終了後も長期間にわたり特定の患者の調査を行うこと、及び取得した情報を適切に管理する事」を目的として、患者の個人情報を利用する。

また、本研究の情報は本研究薬を創製したロシュ社（スイス）及びその関連会社、協力者、並びに提携業者への提供を予定しており、提供の際には提供先の国の法規制*に従った適正な対応をする旨を患者に対し説明し、同意を取得する

* （参考情報）個人情報保護委員会 HP　国外の法令
https://www.ppc.go.jp/enforcement/infoprovision/laws/

2) 利用項目

患者の同定や照会のために最低限必要と考え、利用する項目は下記のとおりとする。

- 患者識別番号（実施医療機関にて付与）、生年月

すなわち、患者氏名等、上記以外の個人情報が実施医療機関からデータセンターへ知らされることはなく、もし誤って知らされた場合には、記録媒体によらず破棄するか、若しくはマスキング等判読不能とする適切な処理を行った上で保管する。

3) 利用方法

患者の個人情報及び診療情報は、eCRF に実施医療機関の研究責任医師、研究分担医師又は研究協力者が入力することにより収集する。なお、電子メールによる個人情報のやりとりは行わない。eCRF の記入データに基づいた中央モニタリングと施設訪問により直接カルテ・診療記録等のデータを閲覧する場合があるが、これらモニタリングや監査等の目的で個人情報に触れたとしても、契約書や守秘義務によってそれらの情報が外部に流出する恐れはない。

10.4.2　データの2次利用について

本研究で得られた試料・情報については、研究代表者及び共同研究機関の協議並びに CRB の審査及び承認を得て承認された場合に、国内や海外で試料・データを 2 次利用することがあり得る。その際には、国内外の関連法規制に従った適正な対応をする。データの 2 次利用の可能性については、説明文書に記載し、あらかじめ研究対象者の同意を得る。一般データ保護規則及び各国のデータプライバシー要件を満たすため、研究代表者や研究共同機関が外国を含む第三者（他の医療機関や製薬企業等）へ臨床研究データを提供する場合には、個人を特定できないよう臨床研究データを管理し、データの 2 次利用者には匿名化された患者データへのアクセス権のみが付与される。

CONFIDENTIAL

10.4.3　情報開示等に対する対応

本研究に参加した患者から、本人が識別される保有個人情報の開示を求められたときは、原則として当該保有個人情報を開示しなければならない。ただし、開示することにより、患者又は第三者の生命、身体、財産その他の権利利益を害する恐れがある場合や、本研究に係る研究者等の業務の適正な実施に著しい支障を及ぼす恐れがある場合は、当該保有個人情報の全部又は一部を開示しないことができる。開示を求められた個人情報の全部又は一部について開示しない旨を決定したときは、患者にその旨を通知し、理由を説明しなければならない。

10.4.4　情報管理体制

個人情報等の利用に際しての情報流出のリスクを最小化すべく安全管理対策を講じる。

10.5　遺伝子カウンセリングの必要性及びその体制

本研究では遺伝情報を取り扱わないため、遺伝カウンセリングは実施しない。

10.6　研究実施計画書の遵守

本研究に参加する研究者は、患者の安全と人権を損なわない限り、本研究実施計画書を遵守する。

10.7　CRB の承認及び厚生労働省への届け出

本研究の実施に際しては、本研究の研究実施計画書及び患者への説明文書を用いて研究を実施することについて、CRB の承認、及び実施医療機関の管理者の研究実施許可を得なければならない。また、研究の開始に先立って、厚生労働大臣への実施計画*の提出、jRCT への研究情報の公表を行わなければならない。研究代表医師は、CRB への申請、実施計画の厚生労働大臣への提出、jRCT への登録に対して責任を負い、支援事務局はこれらの各申請手続きを支援する。

* 臨床研究法施行規則第 39 条第 1 項に規定する省令様式第一

10.7.1　新規申請手続き

＜初回申請から研究開始までの手続き＞

臨床研究法及び関連規則に則り手続きを行う。

「15 研究実施体制」の変更（実施医療機関の追加、入れ替え）は、研究実施計画書の内容の変更に該当するため、「10.8 変更申請時の手続き」に従って変更手続きを行う。

10.7.2　実施医療機関の研究責任医師が行う手続き

実施医療機関の研究責任医師は、CRB の承認が得られた後、所属する実施医療機関の管理者の承認を得る。研究責任医師は、管理者の承認が得られた後、速やかにその旨を支援事務局へ報告する。

10.7.3　実施医療機関での研究実施許可について

所属する実施医療機関の管理者の研究実施許可を得る手順については、実施医療機関の規定に従う。

なお、CRB にて承認された研究実施計画書、患者への説明文書等は実施医療機関の連絡先やあらかじめ指定された選択項目以外の変更は認められない。研究実施計画書や説明文書等の内容の変更が必要であり、実施医療機関の管理者からの修正依頼があった場合は、研究代表医師、支援事務局に相談すること。

10.8　変更申請時の手続き

10.8.1　研究代表医師が行う手続き

研究代表医師は、CRB へ新規申請時に提出した資料の内容変更の必要が生じた場合は、CRB へ変更に関する審議依頼を行う。

なお、当該変更の内容が実施計画の変更を伴う場合は変更前に研究代表医師が CRB の承認を得た後に、jRCT への登録及び厚生労働大臣へ届け出を行い、その旨を速やかに CRB、実施医療機関の管理者、及び各研究責任医師に通知する。

CRB への変更申請手続きは、「10.7.1 新規申請手続き」の＜初回申請から研究開始までの手続き＞に準じる。

省令で定める軽微な変更は、変更の日から 10 日以内にその内容を研究代表医師が CRB に通知するとともに、jRCT へ登録し、厚生労働大臣へ届け出る。また、一部の実施医療機関において研究を継続しなくなった場合は、当該実施医療機関における患者に対する観察期間が終了した後に実施計画の変更を提出する。

10.8.2　実施医療機関の研究責任医師が行う手続き

実施医療機関の研究責任医師は、研究代表医師から CRB の承認に関する情報を受けた場合は、所属する実施医療機関の管理者の承認を得る。実施医療機関の研究責任医師は、管理者の承認が得られた後、速やかにその旨を支援事務局へ報告する。

なお、実施計画の変更が生じない変更においては、実施医療機関の研究責任医師は、実施医療機関の管理者へ報告を行う。

その他、実施医療機関の研究責任医師は、自身が所属する実施医療機関の体制に変更が生じる場合、必要に応じて利益相反に関する書類、研究分担医師リスト等を作成の上、研究代表医師及び支援事務局へ連絡する。

10.8.3　研究の進捗状況や研究継続に関する審査・承認（定期報告）

研究代表医師は、以下 1)～5)の実施状況について実施医療機関の管理者に報告の上、CRB へ定期報告を行い、研究継続の適否について審査を受ける。CRB が意見を述べた日から起算して 1 カ月以内かつ、初回の実施計画を提出した日から起算して 1 年ごとに当該期間満了後 2 カ月以内に厚生労働大臣へ報告する。また、研究代表医師は CRB に報告を行ったときは、その旨を速やかに実施医療機関の研究責任医師へ情報提供を行い、実施医療機関の研究責任医師は、各自の実施医療機関の管理者に対して報告を行う。

1) 本研究の対象患者数（実施予定症例数、同意取得症例数、実施症例数、完了症例数、中止症例数及び補償を行った件数）
2) 本研究に係る疾病等の発生状況及びその後の経過
3) 本研究に係る省令又は研究実施計画書に対する不適合の発生状況及びその後の対応
4) 本研究の安全性及び科学的妥当性についての評価
5) 本研究の利益相反管理基準及び利益相反管理計画に変更があった場合は該当する事項

10.8.4　臨床研究の進捗状況等報告

支援事務局は適宜、本研究の進捗並びに疾病等及び不具合等の発生状況に関する情報を管理する。定期報告の手続きに際しては、研究代表医師へ必要な情報を提供する。

10.9 利益相反

10.9.1 本研究に係る利益相反管理について

本研究に係る利益相反は、「臨床研究法における利益相反管理ガイダンス」（平成 30 年 11 月 30 日医政研発 1130 第 17 号厚生労働省医政局研究開発振興課長通知）*（以下、ガイダンス）に従う。

10.9.2 臨床研究の資金源／資金提供及び財政上の関係

本研究は、研究代表医師が所属している三重大学大学院医学系研究科　臨床医学系講座、業務の一部を委託されている IQVIA サービシーズ ジャパン合同会社、及び中外製薬株式会社との契約に基づき、中外製薬株式会社から本研究に関する資金提供が行われる。実施医療機関は、IQVIA サービシーズ ジャパン合同会社と委受託契約を締結し、登録数等、臨床研究の業務に応じた研究費が支給される。登録推進のために支給されるものではなく、あくまで臨床研究に係る業務負担に対する対価である。なお、本研究は中外製薬株式会社が製造・販売する製剤を用いて行なわれるが、本研究のモニタリング、データマネジメント、統計解析、監査の実施へは直接関与せず、研究結果に影響を与える状況にはない。

10.9.3 情報公開

本研究に関連する研究資金等の提供に関する情報について、公開の対象とする。また、該当者の所属する機関に対する提供も公開対象とする。これらの情報は、中外製薬株式会社ホームページに毎事業年度終了後に公開する。

なお、本研究に参加する医師の中には、資金提供者である中外製薬株式会社から利益を受領している者がいるが、中外製薬株式会社と医師との間に存在する利益相反は、三重大学　臨床研究審査委員会にて審議され、利益相反状態が適切に管理されていることを確認することによって、研究に参加する患者に不利益が及ぶ恐れはないと判断されている。

10.10 研究の費用

10.10.1 治療に関する費用

本研究は通常の健康保険の範囲で行われる。研究期間中の観察・検査、使用薬剤等は患者の健康保険が適用され、患者が保険診療内で負担する（通院のための交通費等も患者が負担する）。ただし、本研究に参加することにより、通常の診療より来院回数が増えることがある。そのため、ファリシマブの投与がない観察・検査のための来院時の交通費に対する負担軽減費として、来院ごとに 3,000 円（交通費が 3,000 円を上回る場合は、実施医療機関の了承の上最大 7,000 円まで実費）を支払う。

10.10.2 健康被害に関する補償

本研究中に有害事象が発現した場合、研究責任医師は速やかに必要な対応（検査、治療あるいは研究中止等）を行い、患者の安全確保に努める。その際、保険診療の範囲内で最善の医療を提供するものとする。研究代表医師及び共同研究者は、臨床研究に起因する健康被害に対して、法律上の賠償責任及び健康被害の補償に備え、本研究開始前に臨床研究補償保険に加入する等の必要な措置を講じるものとする。

また、研究責任医師は、本研究の実施に伴い生じた健康被害に対して、医療を提供する体制の確保とその他の必要な措置を講じておく。

11. 品質管理及び品質保証

11.1　研究の記録

研究責任医師は、研究の実施状況を完全に記録するため、研究実施計画書、説明文書・同意文書､実施医療機関の管理者による承認文書を含む十分かつ正確な記録を保管しなければならない。

11.2　モニタリング

研究が安全に、かつ研究実施計画書に従って実施されているか、データが正確に収集されているかを確認する目的で、研究代表医師が指名した支援事務局の担当者によりモニタリングが実施される。モニタリングでは、データセンターに集積される eCRF の入力データに基づいて行われる中央モニタリングを実施する。また、実施にあたっては、別途定める「モニタリング手順書」に従うものとする。

11.2.1　施設訪問モニタリング

施設訪問モニタリングの頻度及び手順の詳細については、別途定める「モニタリング手順書」に従う。なお、共同研究機関は、施設訪問による患者の個人情報が含まれる原資料の直接閲覧は行わない。

11.2.2　監査

研究の科学的・倫理的な質の向上を目的に、研究代表医師が指名した支援事務局の担当者による施設訪問監査を行うことを検討する。施設訪問監査における確認事項や頻度含めた手順については別途定める。監査に従事する支援事務局の監査担当者は、当該監査の結果を研究責任医師に報告する。研究代表医師の指示により必要に応じてその内容を他の研究責任医師に情報提供を行う。

11.3　研究実施計画書からの逸脱・違反

本研究では、研究実施計画書の規定に従って行われなかったものを逸脱・違反とし、臨床研究が臨床研究法施行規則又は研究実施計画書に適合していない状態（不適合）であるとみなす。研究責任医師又は研究分担医師等は研究実施計画書の規定に従って行われなかった事実や情報を認めた場合は、医療機関の管理者に報告する。特に重大なものが判明した場合においては、速やかに CRB の意見を聴く。

1)　違反（violation）

原則として以下の複数項目に該当する研究実施計画書の規定からの逸脱を「違反」とする。

①　研究の評価項目に影響を及ぼす
②　研究責任医師又は研究分担医師／実施医療機関に原因がある
③　故意又は系統的
④　危険又は逸脱の程度が著しい

2)　逸脱（deviation）

「違反」及び「許容範囲」にも該当しない逸脱。特定の逸脱が多く見られた場合は、論文公表の際に記載することが望ましい。

逸脱　：望ましくないもので減らすべきもの
逸脱（やむを得ない）：積極的に減らすほどでないもの
逸脱（臨床的に妥当）：研究責任医師又は研究分担医師／施設判断を積極的に肯定するもの

3)　許容範囲（acceptable deviation）

研究代表者で事前に又は事後的に設定された許容範囲内の研究実施計画書からの逸脱。

12. データの公表

12.1　公開データベースへの記録

1) 実施計画の公表

厚生労働省が整備するデータベース（jRCT）に、あらかじめ本研究の情報を記録することにより、公表する。実施計画を変更した場合も同様に、変更後の情報を記録することにより、公表する。

2) 主要評価項目報告書の公表

研究実施計画書に記載した主たる評価項目に係るデータの収集を行うための期間が、全ての実施医療機関において終了したときは、研究責任（代表）医師は、原則としてその日から 1 年以内に主要評価項目報告書（主要評価項目に関するデータ収集の結果等を取りまとめた概要）を作成するとともに、実施計画の変更を行う。主要評価項目報告書及び変更後の実施計画についてあらかじめ CRB の意見を聴き、遅延なく医療機関の管理者に提出するとともに、CRB が意見を述べた日から 1 カ月以内に jRCT に記録することにより、公表する。

3) 総括報告書の概要の公表

研究実施計画書に記載した全ての評価項目に係るデータの収集を行うための期間が、全ての実施医療機関において終了したときは、研究責任（代表）医師は、原則としてその日から 1 年以内に総括報告書（本研究の結果等を取りまとめた文書）及びその概要（jRCT における研究結果の概要を登録したものでも可）を作成する。総括報告書には、少なくとも以下の①～④の事項を含める。

① 臨床研究の対象者の背景情報（年齢、性別等）
② 臨床研究のデザインに応じた進行状況に関する情報（対象者の推移等）
③ 疾病等の発生状況のまとめ
④ 主要評価項目及び副次評価項目のデータ解析及び結果

作成した総括報告書及びその概要について、あらかじめ CRB の意見を聴き、遅延なく実施医療機関の管理者に提出する。総括報告書の概要は、CRB が意見を述べた日から 1 カ月以内に jRCT に記録することにより、公表する。また、総括報告書の概要に添えて、研究実施計画書、説明文書、SAP を厚生労働大臣に提出する。なお、主要評価項目報告書及び統括報告書を作成しなければならない時期が同時期の場合は、総括報告書の作成により主要評価項目報告書を作成したものとみなす。

12.2　データの公表

本研究で得られた成果を論文又は学会にて発表する場合は、研究代表医師、研究責任医師及び共同研究機関が協議の上取り決めを行う。

12.3　Layperson Summary（LPS）

主論文公表後、論文にて公表された研究結果・データ等に基づき、研究に参加した患者及びその家族等（患者等）へ研究結果を説明する資料である Layperson Summary（LPS）を作成する。LPS は、研究者が患者等から研究結果の説明を求められた場合に限り、研究者から患者等へ研究結果を説明する際の資料として使用する。

13. 研究全体の中止・中断・終了

13.1　研究全体の終了

実施責任組織により研究全体の終了又は中止を決定し、総括報告書が実施責任組織の承認を得た時点をもって、実施責任組織は「研究終了」を研究代表医師に通知する。

13.2　研究全体の中止・中断

実施責任組織は、研究薬の製造販売承認の取り消しや、予期せぬ重篤な有害事象が認められ緊急安全性情報（イエローレター）の配布等、緊急に安全対策を取る必要がある場合、研究実行委員に医学的・倫理的意見を確認した上、研究全体の中止・中断を判断する。研究の進行中に研究全体を中止又は中断せざるを得ないと判断した場合、実施責任組織は研究の中止・中断及びその理由の詳細を実施医療機関の管理者に速やかに文書にて通知する。実施医療機関の管理者は、研究責任医師及び CRB にその旨を通知し、文書で詳細を説明する。研究責任医師又は研究分担医師は患者にその旨を通知し、適切な治療及び事後処理を保証する。

13.3　総括報告書

支援事務局は、本研究の終了又は中止の連絡を受けたときは、本研究の結果等を取りまとめた総括報告書を作成し、遅滞なく、実施責任組織に対して、総括報告書（案）の提出を行う。実施責任組織により結果の解釈、最終化を行う。

13.4　研究終了時の手続き

研究終了を確認した研究代表医師は、結果の概要とともに研究が終了したことの報告を実施医療機関の研究責任医師に対して行う。研究結果の概要（研究終了報告書）の配布のタイミングは、主たる結果の公表時期等を考慮して実施責任組織及び研究代表医師が決定し、支援事務局を通じて研究終了報告書を実施医療機関の研究責任医師に配布する。研究終了の報告を受けた実施医療機関の研究責任医師は、実施医療機関の規定に従い、速やかに終了の手続きを行う。なお、患者登録がなかった実施医療機関においては、登録終了日をもって当該実施医療機関の研究終了日としてもよい。

14. 研究成果の帰属

本研究により生じた成果は、国立大学法人三重大学及び共同研究機関である中外製薬株式会社に帰属する。得られたデータを用いた研究の成果としての発明及びそれに派生する特許、改良、ノウハウ等の知的財産権の帰属及び管理は、国立大学法人三重大学及び共同研究機関である中外製薬株式会社にて協議の上決定する。中外製薬株式会社が製造・販売する製剤に関する知的財産権は中外製薬株式会社に帰属する。

15. 研究実施体制

15.1　実施責任組織

三重大学大学院医学系研究科　臨床医学系講座

〒514-8507　三重県津市江戸橋二丁目 174
Tel. 059-232-1111（代表）

15.1.1　研究代表医師

三重大学大学院医学系研究科　臨床医学系講座
眼科学　教授　近藤　峰生
〒514-8507　三重県津市江戸橋二丁目 174

Tel. 059-232-1111（代表）

＜研究代表医師の役割及び責務＞

本研究のすべての実施医療機関を含む研究組織全体を取りまとめる役割を担い、臨床研究法を遵守した研究を実施する責任を負う。

15.2　共同研究機関

中外製薬株式会社
〒103-8324　東京都中央区日本橋室町 2-1-1
Tel. 03-3281-6611

15.2.1　　共同研究機関の長

中外製薬株式会社　代表取締役社長　奥田　修

＜共同研究機関の長の役割及び責務＞

本研究が共同研究機関において「臨床研究法」及び研究実施計画書に従って、適正に実施されている事を監督する。共同機関社内規程及び研究実施計画書に反した場合には適切な措置を行う。

15.2.2　　共同研究機関統括責任者

中外製薬株式会社　メディカルアフェアーズ本部　本部長　西　和彦
〒103-8324　東京都中央区日本橋室町 2-1-1
Tel. 03-3281-6611

＜共同研究機関統括責任者の役割及び責務＞

1) 本研究に対する資金提供に関する契約を締結する
2) 研究に対する総括的な監督
   ① 本研究において、共同研究機関における業務が適正に実施されるよう、必要な監督を行うことについての責任を負うものとする。
   ② 本研究において、共同研究機関における業務が前述の指針及び研究実施計画書に従い、適正に実施されていることを必要に応じて確認するとともに、研究の適正な実施を確保するために必要な措置を行う。
   ③ 共同研究機関において、実施を許可した研究の実施に携わる関係者に、患者等及びその関係者の生命、健康及び人権を尊重して研究を実施すること及び共同研究機関社内規程、研究実施計画書に反した場合には懲戒処分等の不利益処分がなされ得ることを周知徹底しなければならない。
   ④ 業務上知り得た情報を正当な理由なく漏らしてはならない。業務に従事しなくなった後も同様とする。
   ⑤ 共同研究機関において、実施を許可した研究に関する業務の一部を委託する場合には、委託を受けた者が遵守すべき事項について、文書による契約を締結するとともに、委託を受けた者に対する必要かつ適切な監督を行わなければならない。
3) 本研究に対する業務実施のための体制・規程の整備
   ① 共同研究機関において、実施を許可した研究に関連して患者に健康被害が生じた場合、これに対する補償その他の必要な措置が適切に講じられることを確保しなければならない。

② 患者等及びその関係者の人権又は研究者等及びその関係者の権利利益の保護のために必要な措置を講じた上で、研究結果等、研究に関する情報が適切に公表されることを確保しなければならない。
③ 共同研究機関において、実施を許可した研究が共同研究機関社内規程に適合していることについて、必要に応じ、自ら点検及び評価を行い、その結果に基づき適切な対応をとらなければならない。
④ 共同研究機関において、実施を許可した研究の研究者等が研究に関する倫理並びに研究の実施に必要な知識及び技術に関する教育・研修を受けることを確保するための措置を講じなければならない。また、自らもこれらの教育・研修を受けなければならない。

15.2.3 共同研究機関責任者

共同研究機関責任者
中外製薬株式会社　スペシャリティメディカルサイエンス部　メディカルマネージャー
辻村　淳
〒103-8324　東京都中央区日本橋室町 2-1-1
Tel. 03-3281-6611

＜共同研究機関責任者の役割及び責務＞

- 研究代表医師と協力し実施計画を策定する。
- 共同研究責任者は本研究において共同研究機関の分担している業務を研究機関社内規程及び研究実施計画書に従い、適正に実施する責務を負う。
- 本研究の結果の公表内容の研究参加施設等への周知及び説明を行う。

15.3 資金提供者

中外製薬株式会社　メディカルアフェアーズ本部　本部長　西　和彦
〒103-8324　東京都中央区日本橋室町 2-1-1
Tel. 03-3281-6611

＜資金提供者の役割及び責務＞

本研究に関する資金提供及び適正に情報等の公開を行い、本研究について共同研究者としての責任を負う。

15.4 研究事務局

三重大学大学院医学系研究科　臨床医学系講座
眼科学　教授　近藤　峰生
〒514-8507　三重県津市江戸橋二丁目 174
Tel. 059-232-1111（代表）

＜研究事務局の役割＞

医学的判断を伴う、研究実施計画書に関する対応を行う。

15.5 実施医療機関

選定された施設は、研究実施可否について検討を行う。決定した実施医療機関は別紙 1 参照（後日他の医療機関も参加する予定である）。

15.6　統計解析責任者
IQVIA サービシーズ ジャパン合同会社
リアルワールド&アナリティックスソリューションズ（RWAS）
リアルワールド　エビデンスサービシーズ
データセンター／生物統計　高田　桂佑
〒108-0074　東京都港区高輪 4-10-18　京急第 1 ビル
Tel. 03-6859-9500

＜統計解析責任者の役割＞
- 統計解析業務の管理監督
- 解析結果の妥当性の証明

15.7　支援事務局
IQVIA サービシーズ ジャパン合同会社
〒108-0074　東京都港区高輪 4-10-18　京急第 1 ビル
Tel. 03-6859-9500

＜支援事務局の役割＞
研究の窓口として研究運営に関する支援を行う。

15.8　モニタリング担当機関
IQVIA サービシーズ ジャパン合同会社
責任者　井手　智仁、渡辺　達也
〒108-0074　東京都港区高輪 4-10-18　京急第 1 ビル
Tel. 03-6859-9500

＜モニタリング担当機関の役割及び責務＞
モニタリング業務の実務を行う。

15.9　データマネジメント担当機関
IQVIA サービシーズ ジャパン合同会社
責任者　光宗　礼佳
〒108-0074　東京都港区高輪 4-10-18　京急第 1 ビル
Tel. 03-6859-9500

＜データマネジメント担当機関の役割及び責務＞
登録及びデータマネジメント業務の実務を行い、データの品質保証の責任を負う。

15.10 監査業務
IQVIA サービシーズ ジャパン合同会社
責任者　中村　桂
〒108-0074　東京都港区高輪 4-10-18　京急第 1 ビル
Tel. 03-6859-9500

＜監査担当機関の役割及び責務＞
監査業務の実務及び監査内容に対する責任を負う。

15.11 調整管理実務担当者

IQVIA サービシーズ ジャパン合同会社
責任者　荒田　美佐
〒108-0074　東京都港区高輪 4-10-18　京急第 1 ビル
Tel. 03-6859-9500

＜調整管理実務担当者の役割及び責務＞

- 臨床研究の進捗及び予算の管理
- 臨床研究に必要な手続の実施、文書の適切な管理及び収集データの信頼性確保
- 臨床研究に関与する関係者との連絡調整及び情報交換

15.12 画像解析機関

株式会社マイクロン
〒108-0073　東京都港区三田三丁目 13 番 16 号

＜画像解析機関の役割＞
画像解析業務の実務を行う。

15.13 研究実行委員

学校法人愛知医科大学　眼科学講座　教授　瓶井　資弘
東京医科大学八王子医療センター　眼科　教授　志村　雅彦
国立大学法人京都大学大学院　医学研究科　医学専攻感覚運動系　外科学講座　眼科学　教授　辻川　明孝

＜研究実行委員の役割及び責務＞

- 研究の実施に関する医学的判断の決定
- 研究実施計画書の作成・変更に関する助言・提言
- 患者の説明文書・同意文書（見本）の作成／改訂に関する助言・提言
- eCRF の設計・作成に関する助言・提言
- 研究参加候補施設の決定
- 研究の進捗状況の確認
- eCRF 入力推進レターの作成と発信
- パブリケーションプランの検討
- 統計解析計画書の協議
- 総括報告書の合意
- 各実施医療機関の研究責任医師に対する情報共有
- その他、実施責任組織と協議した業務

15.14 医学専門アドバイザー（画像解析）

京都大学医学部附属病院　眼科　特定講師　村岡　勇貴

＜医学専門アドバイザーの役割及び責務＞
画像解析に関する助言、結果の解釈に関する助言を行う。

16. 参考文献

1. Song P, Xu Y, Zha M,et al. Global epidemiology of retinal vein occlusion: a systematic review and meta-analysis of prevalence, incidence, and risk factors. J Glob Health. 2019 Jun;9(1):010427. doi: 10.7189/jogh.09.010427. PMID: 31131101; PMCID: PMC6513508.
2. Tadayoni R, Paris LP, Danzig CJ, et al. and COMINO Investigators. Efficacy and Safety of Faricimab for Macular Edema due to Retinal Vein Occlusion: 24-Week Results from the BALATON and COMINO Trials. Ophthalmology. 2024 Aug;131(8):950-960. doi: 10.1016/j.ophtha.2024.01.029. Epub 2024 Jan 26. PMID: 38280653.
3. Hayreh SS, Zimmerman MB, Podhajsky P. Incidence of various types of retinal vein occlusion and their recurrence and demographic characteristics. Am J Ophthalmol. 1994 Apr 15;117(4):429-41. doi: 10.1016/s0002-9394(14)70001-7. PMID: 8154523.
4. Boyd SR, Zachary I, Chakravarthy U, et al. Correlation of increased vascular endothelial growth factor with neovascularization and permeability in ischemic central vein occlusion. Arch Ophthalmol. 2002 Dec;120(12):1644-50. doi: 10.1001/archopht.120.12.1644. PMID: 12470137.
5. Laouri M, Chen E, Looman M,et al. The burden of disease of retinal vein occlusion: review of the literature. Eye (Lond). 2011 Aug;25(8):981-8. doi: 10.1038/eye.2011.92. Epub 2011 May 6. PMID: 21546916; PMCID: PMC3178209.
6. Yasuda M, Kiyohara Y, Arakawa S,et al. Prevalence and systemic risk factors for retinal vein occlusion in a general Japanese population: the Hisayama study. Invest Ophthalmol Vis Sci. 2010 Jun;51(6):3205-9. doi: 10.1167/iovs.09-4453. Epub 2010 Jan 13. PMID: 20071683.
7. A randomized clinical trial of early panretinal photocoagulation for ischemic central vein occlusion. The Central Vein Occlusion Study Group N report. Ophthalmology. 1995 Oct;102(10):1434-44. PMID: 9097789.
8. Ip MS, Scott IU, VanVeldhuisen PC,et al.; SCORE Study Research Group. A randomized trial comparing the efficacy and safety of intravitreal triamcinolone with observation to treat vision loss associated with macular edema secondary to central retinal vein occlusion: the Standard Care vs Corticosteroid for Retinal Vein Occlusion (SCORE) study report 5. Arch Ophthalmol. 2009 Sep;127(9):1101-14. doi: 10.1001/archophthalmol.2009.234. Erratum in: Arch Ophthalmol. 2009 Dec;127(12):1648. PMID: 19752419; PMCID: PMC2872173.
9. Campochiaro PA, Hafiz G, Mir TA, Scott AW, Solomon S, Zimmer-Galler I, Sodhi A, Duh E, Ying H, Wenick A, Shah SM, Do DV, Nguyen QD, Kherani S, Sophie R. Scatter Photocoagulation Does Not Reduce Macular Edema or Treatment Burden in Patients with Retinal Vein Occlusion: The RELATE Trial. Ophthalmology. 2015 Jul;122(7):1426-37. doi: 10.1016/j.ophtha.2015.04.006. Epub 2015 May 9. PMID: 25972260; PMCID: PMC10020833.
10. Heier JS, Singh RP, Wykoff CCet al. THE ANGIOPOIETIN/TIE PATHWAY IN RETINAL VASCULAR DISEASES: A Review. Retina. 2021 Jan 1;41(1):1-19. doi: 10.1097/IAE.0000000000003003. PMID: 33136975.
11. Joussen AM, Ricci F, Paris LP,et al. Angiopoietin/Tie2 signalling and its role in retinal and choroidal vascular diseases: a review of preclinical data. Eye (Lond). 2021 May;35(5):1305-1316. doi: 10.1038/s41433-020-01377-x. Epub 2021 Feb 9. PMID: 33564135; PMCID: PMC8182896.
12. Saharinen P, Eklund L, Alitalo K. Therapeutic targeting of the angiopoietin-TIE pathway. Nat Rev Drug Discov. 2017 Sep;16(9):635-661. doi: 10.1038/nrd.2016.278. Epub 2017 May 19. PMID: 28529319.
13. Benest AV, Kruse K, Savant S, et al. Angiopoietin-2 is critical for cytokine-induced vascular

leakage. PLoS One. 2013 Aug 5;8(8):e70459. doi: 10.1371/journal.pone.0070459. PMID: 23940579; PMCID: PMC3734283.

14. Regula JT, Lundh von Leithner P, Foxton R, et al. Targeting key angiogenic pathways with a bispecific crossMAb optimized for neovascular eye diseases. EMBO Mol Med. 2016 Nov 2;8(11):1265-1288. doi: 10.15252/emmm.201505889. Erratum in: EMBO Mol Med. 2019 May;11(5):e10666. doi: 10.15252/emmm.201910666. PMID: 27742718; PMCID: PMC5090659.
15. Foxton RH, Uhles S, Grüner S, et al. Efficacy of simultaneous VEGF-A/ANG-2 neutralization in suppressing spontaneous choroidal neovascularization. EMBO Mol Med. 2019 May;11(5):e10204. doi: 10.15252/emmm.201810204. PMID: 31040126; PMCID: PMC6505683.
16. Canonica J, Foxton R, Garrido MG, et al. Delineating effects of angiopoietin-2 inhibition on vascular permeability and inflammation in models of retinal neovascularization and ischemia/reperfusion. Front Cell Neurosci. 2023 Jun 12;17:1192464. doi: 10.3389/fncel.2023.1192464. PMID: 37377777; PMCID: PMC10291265.
17. Heier JS, Khanani AM, Quezada Ruiz C, et al.; TENAYA and LUCERNE Investigators. Efficacy, durability, and safety of intravitreal faricimab up to every 16 weeks for neovascular age-related macular degeneration (TENAYA and LUCERNE): two randomised, double-masked, phase 3, non-inferiority trials. Lancet. 2022 Feb 19;399(10326):729-740. doi: 10.1016/S0140-6736(22)00010-1. Epub 2022 Jan 24. PMID: 35085502.
18. Wykoff CC, Abreu F, Adamis AP, et al.; YOSEMITE and RHINE Investigators. Efficacy, durability, and safety of intravitreal faricimab with extended dosing up to every 16 weeks in patients with diabetic macular oedema (YOSEMITE and RHINE): two randomised, double-masked, phase 3 trials. Lancet. 2022 Feb 19;399(10326):741-755. doi: 10.1016/S0140-6736(22)00018-6. Epub 2022 Jan 24. PMID: 35085503.
19. バビースモ添付文書．2024 年 3 月改訂（第 3 版）．中外製薬株式会社
https://www.pmda.go.jp/PmdaSearch/iyakuDetail/ResultDataSetPDF/450045_1319408A1020_1_03
最終アクセス日：2024年9月20日
20. Campochiaro PA, Heier JS, Feiner L, et al.; BRAVO Investigators. Ranibizumab for macular edema following branch retinal vein occlusion: six-month primary end point results of a phase III study. Ophthalmology. 2010 Jun;117(6):1102-1112.e1. doi: 10.1016/j.ophtha.2010.02.021. Epub 2010 Apr 15. PMID: 20398941.
21. Tadayoni R, Waldstein SM, Boscia F, et al.; BRIGHTER Study Group. Sustained Benefits of Ranibizumab with or without Laser in Branch Retinal Vein Occlusion: 24-Month Results of the BRIGHTER Study. Ophthalmology. 2017 Dec;124(12):1778-1787. doi: 10.1016/j.ophtha.2017.06.027. Epub 2017 Aug 12. Erratum in: Ophthalmology. 2018 Mar;125(3):463. doi: 10.1016/j.ophtha.2017.12.012. PMID: 28807635.
22. Campochiaro PA, Clark WL, Boyer DS, et al. Intravitreal aflibercept for macular edema following branch retinal vein occlusion: the 24-week results of the VIBRANT study. Ophthalmology. 2015 Mar;122(3):538-44. doi: 10.1016/j.ophtha.2014.08.031. Epub 2014 Oct 12. PMID: 25315663.
23. Brown DM, Campochiaro PA, Singh RP, et al.; CRUISE Investigators. Ranibizumab for macular edema following central retinal vein occlusion: six-month primary end point results of a phase III study. Ophthalmology. 2010 Jun;117(6):1124-1133.e1. doi: 10.1016/j.ophtha.2010.02.022. Epub 2010 Apr 9. PMID: 20381871.
24. Ogura Y, Roider J, Korobelnik JF, et al.; GALILEO Study Group. Intravitreal aflibercept for

macular edema secondary to central retinal vein occlusion: 18-month results of the phase 3 GALILEO study. Am J Ophthalmol. 2014 Nov;158(5):1032-8. doi: 10.1016/j.ajo.2014.07.027. Epub 2014 Jul 25. PMID: 25068637.

25. Heier JS, Clark WL, Boyer DS, et al.. Intravitreal aflibercept injection for macular edema due to central retinal vein occlusion: two-year results from the COPERNICUS study. Ophthalmology. 2014 Jul;121(7):1414-1420.e1. doi: 10.1016/j.ophtha.2014.01.027. Epub 2014 Mar 27. Erratum in: Ophthalmology. 2014 Nov;121(11):2293. PMID: 24679444.
26. Heier JS, Campochiaro PA, Yau L, Li Z, et al. Ranibizumab for macular edema due to retinal vein occlusions: long-term follow-up in the HORIZON trial. Ophthalmology. 2012 Apr;119(4):802-9. doi: 10.1016/j.ophtha.2011.12.005. Epub 2012 Feb 1. PMID: 22301066.
27. Shimura M, Fukumatsu M, Tsujimura J,et al.; Participating Investigators. Real-World Data on Intravitreal Aflibercept for Macular Edema Secondary to Central Retinal Vein Occlusion: 24-Month Outcomes. Clin Ophthalmol. 2022 Mar 1;16:579-592. doi: 10.2147/OPTH.S344194. PMID: 35256840; PMCID: PMC8898177.
28. Shimura M, Kitano S, Muramatsu D, et al.; Japan Clinical Retina Study (J-CREST) group. Real-world management of treatment-naïve diabetic macular oedema in Japan: two-year visual outcomes with and without anti-VEGF therapy in the STREAT-DME study. Br J Ophthalmol. 2020 Sep;104(9):1209-1215. doi: 10.1136/bjophthalmol-2019-315199. Epub 2019 Nov 29. PMID: 31784500; PMCID: PMC7577088.
29. Hattenbach LO, Abreu F, Arrisi P, et al. BALATON and COMINO: Phase III Randomized Clinical Trials of Faricimab for Retinal Vein Occlusion: Study Design and Rationale. Ophthalmol Sci. 2023 Mar 27;3(3):100302. doi: 10.1016/j.xops.2023.100302. PMID: 37810589; PMCID: PMC10556281.
30. Sheryl Stevenson. Angiogenesis 2024: Highlighting 72-week results from the BALATON and COMINO Phase 3 studies of faricimab in RVO. 2024 Feb. https://www.modernretina.com/view/angiogenesis-2024-highlighting-72-week-results-from-the-balaton-and-comino-phase-3-studies-of-faricimab-in-rvo
31. Shimura M, Fukumatsu M, Tsujimura J,, et al.; Participating Investigators. Real-world data on intravitreal aflibercept for macular edema secondary to central retinal vein occlusion: 24-month outcomes. Clin Ophthalmol. 2022;16:579–592.
32. Ozdemir S, Finkelstein E, Lee JJ, et al. Understanding patient preferences in anti-VEGF treatment options for age-related macular degeneration. PLoS One. 2022 Aug 11;17(8):e0272301. doi: 10.1371/journal.pone.0272301. PMID: 35951503; PMCID: PMC9371344.
33. バビースモ 硝子体内注射液 120mg/mL 適正使用ガイド．2024 年 3 月．中外製薬株式会社 https://www.pmda.go.jp/RMP/www/450045/2e72f5fb-b81f-41ce-b2c7-d662f67b5d94/450045_1319408A1020_01_003RMPm.pdf
最終アクセス日：2024 年 9 月 20 日
34. Gregori NZ, Feuer W, Rosenfeld PJ. Novel method for analyzing snellen visual acuity measurements. Retina. 2010 Jul-Aug;30(7):1046-50. doi: 10.1097/IAE.0b013e3181d87e04. PMID: 20559157.

Appendix 1　研究実施スケジュール

	スクリーニング	W0	W4	W8	W12	W16	W20	W24	W28	W32	W36	W40	W44	W48	W52	W56	W60	W64	W68	W72	規定外
来院の許容範囲（日）	D-14～D1	D1	(-7, +14)	(-7, +14)	(-7, +14)	(-7, +14)	(-7, +14)	(-7, +14)	(-7, +14)	(-7, +14)	(-7, +14)	(-7, +14)	(-7, +14)	(-7, +14)	(-7, +14)	(-7, +14)	(-7, +14)	(-7, +14)	(-7, +14)	(-7, +14)	
規定来院	○	○	○	○	○	○	○	○			○[a]				○					○	
同意取得	○[b]																				
選択・除外基準の確認	○																				
病歴及び手術歴	○																				
患者背景	○																				
血圧（収縮期、拡張期）	○	○[c]													○					○	
屈折検査[d]	○	○[c]																			
眼軸長検査[d]	●	●[c]																			
研究薬投与[e]		←																		→	●
併用薬・併用療法		○	○	○	○	○	○	○	○[f]	○[f]	○[f]	○[f]	○[f]	○[f]	○	○[f]	○[f]	○[f]	○[f]	○	○
有害事象		←																		→	○
視力検査[d, g]	○	○[c]	○	○	○	○	○	○	○[f]	○[f]	○[f]	○[f]	○[f]	○[f]	○	○[f]	○[f]	○[f]	○[f]	○	○
眼圧検査[d, h]	○	○[c]	○	○	○	○	○	○	○[f]	○[f]	○[f]	○[f]	○[f]	○[f]	○	○[f]	○[f]	○[f]	○[f]	○	○
SD-OCT 又は SS-OCT[d, i]	○	○[c]	○	○	○	○	○	○	○[f]	○[f]	○[f]	○[f]	○[f]	○[f]	○	○[f]	○[f]	○[f]	○[f]	○	○
OCT-A[d, i]	○	○[c]	○	○	○	○	○	○	○[f]	○[f]	○[f]	○[f]	○[f]	○[f]	○	○[f]	○[f]	○[f]	○[f]	○	○
FA[d, j]	○	○[c]													○						
CFP[d, j]	○	○[c]													○						

CFP = カラー眼底写真、FA = フルオレセイン蛍光眼底造影、OCT-A = OCT アンギオグラフィー、SD-OCT = スペクトルドメイン型光干渉断層計、SS-OCT = 波長掃引型光干渉断層撮影、W = Week、D = Day、○：必須項目、●：任意項目

a. 観察期の患者のみ規定来院とする。

b. 同意取得前であっても、Day 1前14日以内に通常診療の中で得られている検査・評価結果を用いることが可能であり、スクリーニングのために再度検査・評価を行う必要はない。

c. スクリーニング時に実施していれば、Day 1は不要とする。

d. 眼科検査は対象眼では必須とする。

e. Day 1の研究薬の投与は同意取得後28日以内に実施し、以降の研究薬の投与は少なくとも21日あけること。研究薬の投与後15分以内に指数弁を評価し、視機能に問題がないことを確認する。

f. 来院した際に測定する。

g. ランドルト環視力表を用い、5 m の距離で実施する。

h. 眼科検査のための散瞳前に対象眼の眼圧を測定し、30 mmHg 以上であれば散瞳薬及び研究薬の投与を中止する。また、可能な限り研究薬の投与30分後にも対象眼の眼圧を測定する。

i. 原則、散瞳下で実施する。

j. 蛍光眼底造影及びカラー眼底撮影手順書に従い実施する。

Appendix 2　eCRF の入力項目

(1) 登録票

調査項目	収集項目
患者背景	患者識別番号<br>性別<br>初回同意取得時の年齢<br>生年月<br>人種
投与開始予定日	投与開始予定日
対象眼	対象眼（右眼又は左眼）
同意取得	文書同意取得の有無<br>（有の場合）文書同意取得日
選択・除外基準	選択基準の適否<br>除外基準の適否

(2) ベースライン（登録時の情報）

調査項目	収集項目
既往歴・合併症	既往歴／合併症の有無（有の場合、既往歴／合併症の別、疾患名）
手術歴	手術歴の有無（有の場合、疾患名、術式名、手術日）
手術歴（眼疾患）	手術歴の有無（有の場合、疾患名、対象眼、術式名、手術日）
前治療薬・併用薬剤	薬剤名、投与経路、1日投与量、投与開始日／終了日又は継続中、投与理由
前治療薬・併用薬剤（眼疾患）	薬剤名、対象眼、投与経路、1 日投与量、投与開始日／終了日又は継続中、投与理由
血圧	収縮期／拡張期血圧<br>降圧剤使用の有無
眼圧	眼圧（対象眼）
屈折検査	球面度数、円柱度数、円柱軸（対象眼）<br>異常の有無（対象眼）
眼軸長	眼軸長（対象眼）
視力検査	小数視力（対象眼）<br>（小数視力0.02未満の場合）指数弁、手動弁又は光覚弁
SD-OCT 又は SS-OCT	CST（対象眼）<br>IRF、SRF 及び黄斑上膜の有無（対象眼）
FA	撮像日
CFP	撮像日
OCT-A	網膜浅層血管網、網膜深層血管網及び網膜全層血管網における血管密度

	撮像日

(3) 調査票

調査項目	収集項目
来院	来院日
血圧<br>（W52、W72のみ）	収縮期／拡張期血圧<br>降圧剤使用の有無
眼圧	投与前眼圧（対象眼）
視力検査	小数視力（対象眼）<br>（小数視力0.02未満の場合）指数弁、手動弁又は光覚弁<br>指数弁（投与終了後15分以内）
SD-OCT 又は SS-OCT	CST（対象眼）<br>IRF、SRF 及び黄斑上膜の有無（対象眼）
FA<br>（W52のみ）	撮像日
CFP<br>（W52のみ）	撮像日
OCT-A	網膜浅層血管網、網膜深層血管網及び網膜全層血管網における血管密度<br>撮像日
研究薬の硝子体内投与	投与の有無（有の場合、投与日、投与量）<br>（用量変更の場合その理由）
併用薬剤	併用薬の有無（有の場合、薬剤名、投与経路、1日投与量、投与開始日、投与終了日又は継続中、投与理由）
併用療法	併用療法の有無（有の場合、療法名、治療開始日、治療終了日又は継続中、治療理由）
有害事象	有害事象（有の場合、有害事象名、発現日、重篤度［重篤な場合その理由］、重症度、転帰及び転帰確認日、研究薬との関連性、有害事象に対する研究薬の処置）
投与状況	確認日<br>継続／終了／中止（中止の場合、中止日、中止理由）
